# Dipolar Molecular Motor Candidates with Planar Chirality

**DOI:** 10.1002/chem.202502277

**Published:** 2025-09-29

**Authors:** Thomas A. Hector, Shohei Katao, Nathalie Saffon‐Merceron, Valentin Magné, Claire Kammerer, Gwénaël Rapenne

**Affiliations:** ^1^ CEMES Université de Toulouse CNRS 29, rue Marvig Toulouse 31055 France; ^2^ Division of Materials Science Nara Institute of Science and Technology 8916‐5 Takayama Ikoma 630‐0192 Japan; ^3^ Université de Toulouse Institut de Chimie de Toulouse ICT UAR 2599 118 route de Narbonne Toulouse 31062 France

**Keywords:** chirality, cyclopentadienyl ligand, dipolar molecule, molecular motor, ruthenium

## Abstract

Two molecular motor candidates built around ruthenium(II) centers featuring both planar chirality and a permanent dipole have been synthesized in seven steps. These motors incorporate desymmetrized cyclopentadienyl ligands bearing electron‐donating (4‐methoxyphenyl) and electron‐withdrawing (3,5‐bis(trifluoromethyl)phenyl) groups in 1,2 or 1,3 relative positions, to induce strong dipolar moments and asymmetry helpful for unidirectional rotation. Two dipolar pentaarylcyclopentadienyl ligands were synthesized and coordinated to a Ru(II) trisindazolylborate complex functionalized with thioether moieties for surface anchoring. Structural analysis and DFT calculations revealed that dipole moments increase upon complexation, reaching 5.60 D and 4.58 D for the **1,2‐Cp^Ar5^[Ru]Tp** and **1,3‐Cp^Ar5^[Ru]Tp** complexes, respectively. Key synthetic challenges, such as low yields in Knoevenagel condensations and side reactions, were addressed through careful modulation of precursors, reaction conditions and cation selection. This study demonstrates the feasibility of integrating permanent dipoles and chirality into molecular motor design, which could offer an enhanced control over motion under STM‐induced electric fields.

## Graphical abstract

Two prototypes of molecular motors with simultaneous implementation of planar chirality and a permanent dipole have been designed and synthesized in 7 steps with overall yields of 2.8 to 5.5%.

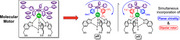

## 1 Introduction

Synthetic molecular machines are nanoscale devices engineered to perform specific mechanical tasks at the molecular level, mimicking the functions of natural molecular machines found in biological systems, such as motor proteins.^[^ ^1^ ^]^ The design and synthesis of these machines involve intricate control of molecular architecture to achieve specific, predictable movements.^[^ ^2^ ^]^ The 2016 Nobel Prize in Chemistry, awarded to Jean‐Pierre Sauvage, Sir Fraser Stoddart, and Bernard Feringa, highlighted the significance of this field,^[^ ^3^ ^]^ marking it as a cornerstone of future molecular nanotechnology advancements. Within this class of molecules, rotary motors that convert energy input into unidirectional rotational motions at the molecular level play a major role.^[^ ^4^ ^]^ Inspired by biological motors such as ATP synthase, many different chemical architectures have been designed to undergo a sequence of conformational changes, driven by external stimuli such as light, chemical reactions, or electrical energy. The key challenge in designing these motors lies in achieving controlled, directional rotation rather than random, Brownian motion. This is accomplished by introducing structural asymmetry and creating energy barriers that guide the rotation in a specific direction. The ability to control molecular motion at such a fine scale opens up exciting possibilities for applications in various fields such as the development of smart materials, targeted drug delivery systems, and molecular computing.^[^ ^5^ ^]^

**Figure 1:**
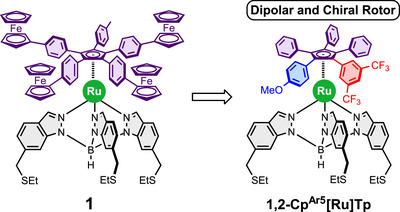
First generation of Ru(II) molecular motor (left) and its chemical modification to integrate planar chirality and a permanent dipole (right).

In this context, we reported the design and synthesis of an electron‐driven molecular motor (**1**) featuring a dissymmetrically substituted cyclopentadienyl (Cp) rotor, a tripodal ligand, serving as the stator, functionalized with thioether groups to ensure strong anchoring to a gold surface via chemisorption, and a ruthenium(II) center acting as a ball‐bearing to facilitate rotary motion between the upper and lower subunits (Figure 1, left).^[^ ^6^ ^]^ This molecular motor was investigated at the single molecular level under Ultra‐High Vacuum (UHV) conditions at low temperature (5 K) using Scanning Tunneling Microscopy (STM).^[^ ^7^ ^]^ The latter enabled not only high‐resolution imaging of the molecular motor but also the induction of rotation through the injection of tunneling electrons.

The rotor's dissymmetry, comprising four phenyleneferrocenyl groups and a truncated tolyl arm, was critical for its function. This specific design allowed direct monitoring of the rotational motion and its direction by tracking the position of the shorter arm, which serves as a marker. Furthermore, such ruthenium complexes are not chiral in solution but become chiral once anchored to a surface due to the twisting of the tripodal ligand.^[^ ^7^ ^]^ Thus, the combination of rotor dissymmetry and the helical twisting of the tripodal ligand induced a reversible rotational behavior: the direction of rotation was determined by the STM tip's location during the pulse, either above one of the longer ferrocenyl‐appended arms or above the shorter truncated arm.

To further enhance our design, we envisioned the preparation of a permanently chiral ruthenium complex, instead of relying on the twisting of the tripodal ligand on the surface to induce asymmetry. Chirality alone is not sufficient to achieve unidirectional rotation in motion driven solely by thermal energy,^[^ ^8^ ^]^ as this would violate the second law of thermodynamics. However, chirality is a critical feature in synthetic molecular motors, as it introduces additional dissymmetry responsible for the modulation of energy barriers governing the directionality of the rotation.^[^ ^7^ ^]^ One strategy to introduce intrinsic chirality into the motor architecture is to modify the pentaarylcyclopentadienyl ligand by attaching different chemical groups, giving rise to planar chirality. Although synthetically challenging, this approach also allows to incorporate a permanent dipole in the rotating subunit, thus adding another layer of functionality and control. This is an already established and efficient method for controlling molecular motions not only through inelastic electron tunneling, but also via the application of an electric field.^[^ ^9^ ^]^ Indeed, a dipolar molecule features a separation of electric charges, which can be exploited to interact with an external electric field, providing an additional mechanism to control both the orientation and rotation of the motor. In this article, we present the design and synthesis of two new molecular motor candidates that simultaneously display intrinsic chirality and a permanent dipole.

## 2 Results and Discussion

### 2.1 Design of the Prochiral Pentaarylcyclopentadienyl Ligands

**Figure 2:**
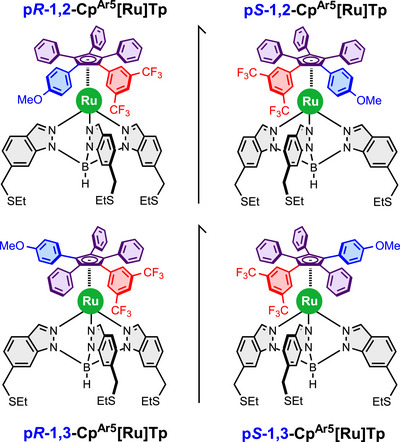
Molecular structure of the target chiral ruthenium complexes with dipolar cyclopentadienyl rotors functionalized in 1,2 and 1,3 positions, depicted with their respective enantiomers.

From the parent pentaphenylcyclopentadienyl ligand, planar prochirality can be achieved by substitution of two phenyl groups with two distinct aromatic rings. By introducing one aromatic ring bearing an electron‐donating group and another with an electron‐accepting group, the cyclopentadienyl ligand can simultaneously exhibit planar prochirality and a significant dipole. As donor, we selected a 4‐methoxyphenyl ring (shown in blue) and as acceptor, a 3,5‐bis(trifluoromethyl)phenyl subunit (shown in red). These substituents are well known for inducing a strong disparity of charge, resulting in a significant dipolar moment. Importantly, both chemical groups are compatible with the conditions subsequently used to prepare the ruthenium complexes. For instance, the use of coordinative groups such as nitrile or aniline is prohibited due to competitive coordination. Depending on the relative positions of the donor and acceptor aryl subunits on the cyclopentadienyl ring, two regioisomers can be envisioned: the **1,2‐Cp^Ar5^** ligand in which the two polar groups occupy adjacent carbons of the cyclopentadienyl core, and the **1,3‐Cp^Ar5^** ligand in which they are separated by one carbon atom. Both ligands will be coordinated to a Ru(II) trisindazolylborate complex bearing three thioether moieties, which serve as anchoring groups for attachment to metal surfaces. Once coordinated, the prochiral Cp ligand will give rise to chiral piano‐stool ruthenium complexes. The structures of the target complexes are shown in Figure 2.

It is worth noting that, unlike the original molecular motor (**1**) shown in Figure 1, our new target structures do not include ferrocene moieties. This is due to our recent findings,^[^ ^10^ ^]^ which revealed that the presence of ferrocenes is not essential for achieving unidirectional rotation; rather, a desymmetrized cyclopentadienyl rotor ligand alone is sufficient to induce such motion.

### 2.2 Calculation of Dipole Moment in 1,2‐ and 1,3‐functionalized Ligands

**Figure 3:**
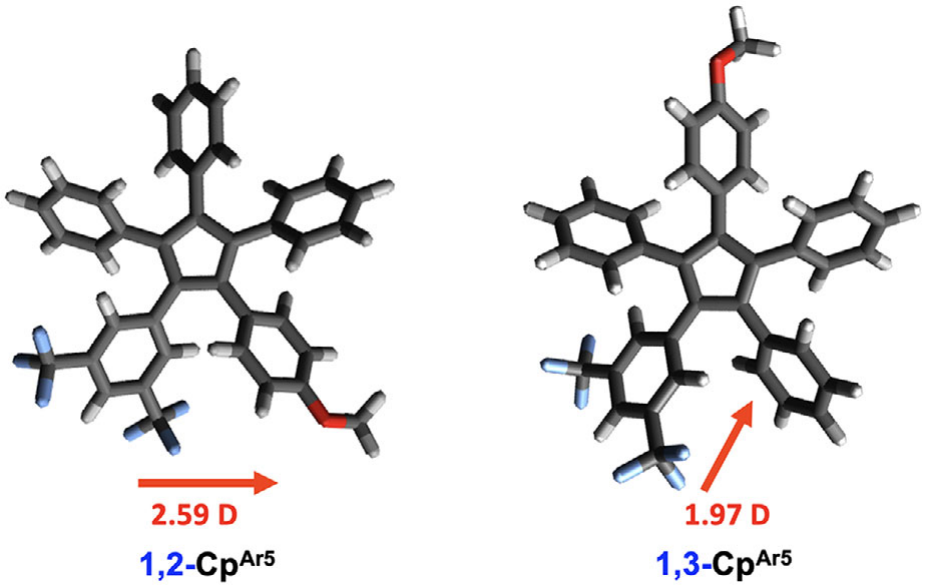
DFT‐optimized geometries of the cyclopentadienyl anionic targets **1,2‐Cp^Ar5^** and **1,3‐Cp^Ar5^** with their associated dipolar moment (F in blue and O in red). BP86 functional was used (base def2/J) with ORCA 5.0.4.

In order to assess the dipole moment of the motor depending on the 1,2‐ or 1,3‐substitution pattern on the pentaarylcyclopentadienyl ring, we investigated how the dipole evolves in the free pentaarylcyclopentadienide ligands. The geometries of the two substituted anionic ligands were optimized using DFT calculations with Orca^[^ ^11^ ^]^ with the BP86 functional (base def2/J), and their dipole moments were subsequently estimated (Figure 3 and SI).

The magnitude of a dipole moment is proportional to both the charge separation and the distance between the charges. Assuming the same charges are involved, as observed in the electron density map of both isomers (SI Figures S50 and S51), increasing the distance between the donor and acceptor groups should lead to a higher dipole moment for the 1,3 isomer. However, calculations revealed that increasing the distance between the 4‐methoxyphenyl and the 3,5‐bis(trifluoromethyl)phenyl groups by moving from the 1,2‐ to the 1,3‐functionalized Cp ligand, actually results in a decrease of the dipole moment from 2.59 to 1.97 D. This counterintuitive result may stem from differences in electronic density distribution between the two anionic systems. In the 1,2‐functionalized cyclopentadienide ligand, the donor and acceptor groups are positioned closely with a very low electron density contribution to the dipole arising from the anionic Cp. In contrast, the 1,3 isomer has a strong electron density from the anionic Cp ring in between the donor and acceptor. This large electron density likely exerts a significant shielding. As a result, the effective charge separation is reduced, and this shielding more than compensates for the increase in distance, leading to an overall decrease in dipole moment.

### 2.3 Synthesis of 1‐(4′‐methoxyphenyl)‐2‐(3′,5′‐bis(trifluoro‐methyl)phenyl)‐3,4,5‐triphenylcyclopentadienyl ligand (1,2‐Cp^Ar5^) and its Ru(II) complex [1,2‐Cp^Ar5^[Ru]Tp]

As mentioned above, planar chirality in the Ru(II) molecular motor is implemented through the incorporation of different aromatic groups in the pentaarylcyclopentadienyl ligand. To simultaneously achieve a strong dipole moment, we designed a cyclopentadienyl ligand bearing one 4‐methoxyphenyl electron‐donating group and one 3,5‐bis(trifluoromethyl)phenyl electron‐withdrawing group.

**Scheme 1:**
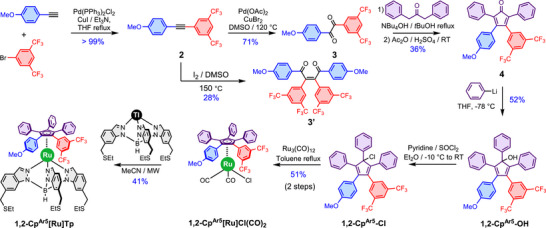
Synthesis of the **1,2‐Cp^Ar5^** ligand and its Ru(II) tripodal complex **1,2‐Cp^Ar5^[Ru]Tp**. Additional details are provided in the experimental part and the SI.

**Figure 4:**
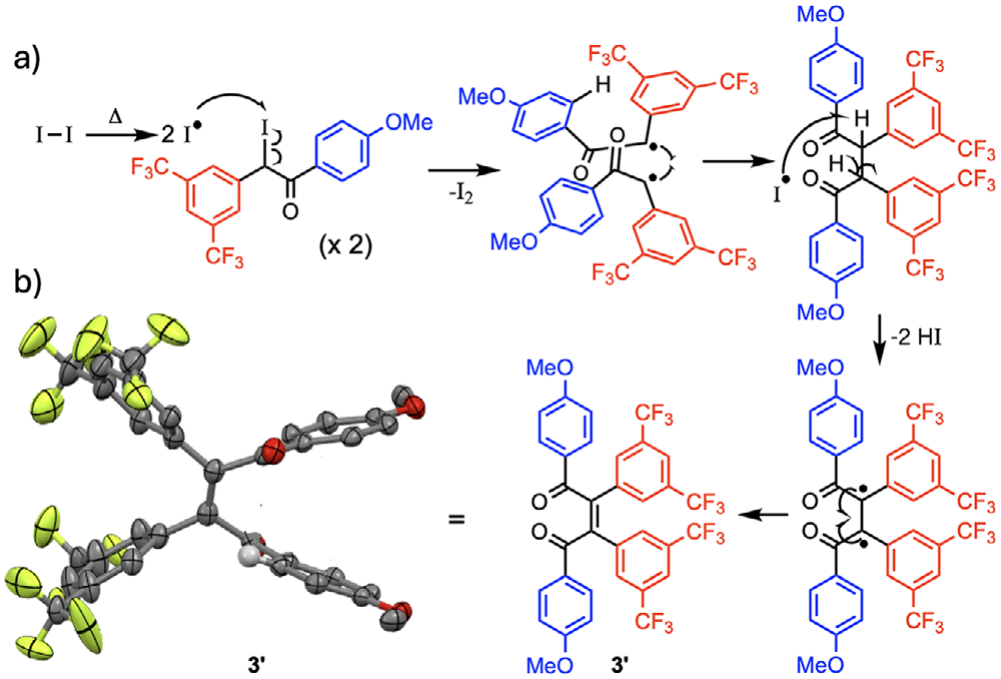
a) Proposed mechanism for the formation of the undesired dimeric compound **3′** and b) the crystal structure of compound **3′** which reveals its *Z*‐configuration.

The synthetic sequence is outlined in Scheme 1. In a first step, the dissymmetric alkyne **2** was obtained quantitatively via a Sonogashira coupling^[^ ^12^ ^]^ between (4‐methoxyphenyl)acetylene and 1‐bromo‐3,5‐bis(trifluoromethyl)benzene. To generate the α‐diketone **3**, required for the subsequent double Knoevenagel condensation with 1,3‐diphenylpropan‐2‐one, the oxidation of the alkyne was attempted using I_2_ in DMSO.^[^ ^13^ ^]^ Unfortunately, the dipolar nature of the alkyne hindered the formation of the desired α‐diketone **3**. Although the desired compound was obtained in a 13% yield, the major product (28%) was isolated and characterized. NMR and X‐ray analysis (Figures S5‐S7 and S47) revealed this compound to be a dimer centered around a *Z*‐configured alkene (compound **3′** in Figure 4).

A plausible mechanism based on radical coupling is illustrated in Figure 4. Because the reaction was conducted under thermodynamic conditions (DMSO at 150 °C for 17 hours), the thermodynamic product was obtained. DFT calculations were performed on both *Z*‐ and *E*‐configuration dimers and it appeared that the *Z*‐configuration is more stable by 14.5 kJ.mol^−1^ (SI page S61). It must be noted that strong stabilizing interactions can be envisioned in both products (*Z*‐ and *E*‐isomer) such as orbital interactions between the carbonyl group and the anisyl ring, a hydrogen bond between the carbonyl group and proton of aryl rings and π‐stacking interactions between the two anisyl groups or donor‐acceptor interactions between one electron rich anisyl and one electron poor 3,5‐bis(trifluoromethyl)phenyl group.

Alternatively, the oxidation of the dissymmetric alkyne **2** with Pd(OAc)_2_ and CuBr_2_, in DMSO^[^ ^14^ ^]^ yielded the desired α‐diketone **3** bearing both an anisyl and a 3,5‐bis(trifluoromethyl)phenyl group in a satisfactory 71% yield. Then, a double Knoevenagel condensation of **3** with the 1,3‐diphenylpropan‐2‐one using the classical conditions^[^ ^15^ ^]^ was unsuccessful with only a few percent detected. After reviewing the literature, it appeared that tetraarylcyclopentadienone formation via a Knoevenagel reaction typically gives very low yields when the α‐diketone is dissymmetric, and very few tetraarylcyclopentadienones have been described. However, in front of similar issues, Jaung et al.^[^ ^16^ ^]^ hypothesized that potassium hydroxide, used as base under classical conditions, was inducing many side reactions by reacting too quickly. They suggested that increasing the steric hindrance of the cation was the key to slowing down these side reactions. To test this, potassium was replaced with benzyltrimethylammonium, a more sterically hindered cation. However, such modification of the cation yielded the same result. We hypothesized that the electronic difference between the two electrophilic carbonyl centers in **3** led to strong competition between inter‐ and intramolecular reactions, resulting in numerous side‐reactions. In our case, the benzyltrimethylammonium hydroxide still reacted too rapidly, prompting us to replace it with an even bulkier cation. We selected the tetrabutylammonium cation, as its use has been reported in the literature for the synthesis of dissymmetric cyclopentadienones.^[^ ^17^ ^]^ Despite this modification, only small amounts of the desired cyclopentadienone **4** were obtained, and TLC analysis revealed spots corresponding to the hydroxylated cyclopentenone intermediate. Previous work by Yang et al.^[^ ^18^ ^]^ described the rapid conversion of such intermediate into the corresponding cyclopentadienone in the presence of acetic anhydride and concentrated sulfuric acid.

Therefore, in the optimized conditions, compound **3** was refluxed with 1,3‐diphenylpropan‐2‐one in *tert*‐butanol using tetrabutylammonium hydroxide as base, followed by treatment of the crude reaction mixture with acetic anhydride and a catalytic amount of concentrated sulfuric acid. This afforded the dissymmetric tetraarylcyclopentadienone **4** as a dark red oil in 36% yield after column chromatography. An additional phenyl substituent was then introduced via nucleophilic addition of phenyllithium to a solution of **4** in THF at ‐78 °C. The target cyclopentadienol **1,2‐Cp^Ar5^‐OH** was obtained as a single regioisomer in a 52% yield.

In the pentaarylcyclopentadiene family, steric hindrance prevents metal coordination through the formation of the pentaarylcyclopentadienide anion generated under basic conditions.^[^ ^19^ ^]^ Ruthenium coordination to this type of ligand can efficiently take place if a halogen atom is present on the sp^3^ carbon of the Cp ring, following the Manner's methodology^[^ ^20^ ^]^ which involves the oxidative addition of the carbon‐bromine bond to the ruthenium carbonyl cluster. As we established that chlorine centers are also effective for oxidative addition^[^ ^21^ ^]^ and enhance the yield by preventing radical formation leading to numerous side products, the hydroxy group in **1,2‐Cp^Ar5^‐OH** was replaced by a chlorine atom. After reaction with SOCl_2_ in pyridine, the chlorinated **1,2‐Cp^Ar5^‐Cl** was reacted with Ru_3_(CO)_12_ in refluxing toluene, yielding **1,2‐Cp^Ar5^[Ru]Cl(CO)_2_** after purification by column chromatography on silica, in 51% yield over two steps. It is important to note that the chlorinated Cp was obtained as a mixture of regioisomers due to the S_N_1 mechanism of this reaction, but the same complex was obtained for all regioisomers by coordination of the Cp ring to the ruthenium center. In a final step, the two carbonyl groups and the chloride ligand were substituted by the tripodal ligand (thallium salt of hydrotris{6‐[(ethylsulfanyl)methyl]indazolyl}borate)^[^ ^22^ ^]^ by heating under microwave irradiation in a sealed tube at 150 °C for 3 × 10 min. The 1,2‐functionalized dipolar motor **1,2‐Cp^Ar5^[Ru]Tp** was obtained in 41% yield and its structure was confirmed by HR‐MS, NMR and X‐ray analysis.

### 2.4 Synthesis of 1‐(4′‐methoxyphenyl)‐3‐(3′,5′‐bis(trifluoro‐methyl)phenyl)‐2,4,5‐triphenylcyclopentadienyl ligand (1,3‐Cp^Ar5^) and its Ru(II) complex [1,3‐Cp^Ar5^[Ru]Tp)

**Scheme 2:**
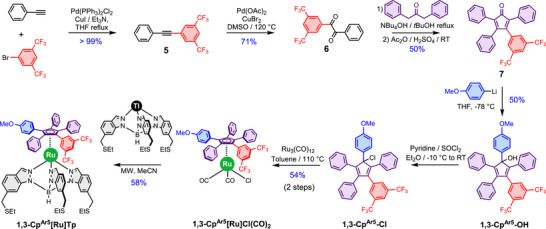
Synthesis of the **1,3‐Cp^Ar5^** ligand and its Ru(II) tripodal complex **1,3‐Cp^Ar5^[Ru]Tp**. Additional details are provided in the experimental part and the SI.

The 1,3‐functionalized Cp ligand and its corresponding Ru(II) complex **1,3‐Cp^Ar5^[Ru]Tp** were synthesized by following a similar synthetic strategy as illustrated in Scheme 2. Our initial goal was to obtain a tetraarylcyclopentadienone bearing three phenyl groups and one anisyl group. To this end, we synthesized 1‐anisyl‐2‐phenylethanedione and reacted it with 1,3‐diphenylpropan‐2‐one in the presence of tetrabutylammonium hydroxide. Despite performing the elimination step using acetic anhydride and sulfuric acid, only low yields of the desired tetraarylcyclopentadienone were obtained. Moreover, these yields were inconsistent across multiple attempts, prompting us to revise our approach. We thus shifted our focus toward a tetraarylcyclopentadienone incorporating a 3,5‐bis(trifluoromethyl)phenyl substituent instead.

Starting from compound **5**,^[^ ^23^ ^]^ we carried out an oxidation of the alkyne to yield the α‐diketone **6**. Condensation of the latter with diphenylacetone using the optimized method to prepare cyclopentadienone **4**, afforded compound **7** as a dark pink solid in a 50% yield.

The successful formation of this cyclopentadienone in good yield contrasts sharply with the poor yield observed for the analogue bearing only electron‐donating groups. This result supports our hypothesis that the electronic difference between the carbonyl centers in the α‐diketone precursor plays a crucial role in the synthesis of these dissymmetric tetraarylcyclopentadienones. Introduction of the anisyl group was achieved via nucleophilic addition of the corresponding lithium reagent at ‐78 °C. The lithium reagent was generated in situ from 4‐bromoanisole via a metal‐halogen exchange using *n*‐butyllithium. This step afforded **1,3‐Cp^Ar5^‐OH** as a yellow solid in 50% yield. Following a strategy analogous to that used for the **1,2‐Cp^Ar5^[Ru]Cl(CO)_2_** complex, **1,3‐Cp^Ar5^[Ru]Cl(CO)_2_** was synthesized from the parent **1,3‐Cp^Ar5^‐OH** in two steps upon chlorination and oxidative addition with an overall yield of 54%. This complex was then converted into the target 1,3‐dipolar motor, **1,3‐Cp^Ar5^[Ru]Tp**, in a final ligand exchange step with a yield of 58%.

The dissymmetry of the complex has been exploited to study the rotation in the ruthenium complexes at room temperature. Since the tripodal ligand bears a C_3_ axis and the coordinated penta‐substituted Cp is of C_1_ symmetry, chemical equivalence in the ^1^H‐NMR spectrum is a proof of free rotation of one ligand with respect to the other. In the ^1^H‐NMR spectrum of both ruthenium complexes (Figures S17 and S33), the rotational freedom of the Cp ring has been evidenced since the three indazolyl groups are equivalent.

### 2.5 X‐ray Structures of Chiral Dipolar Motors

**Figure 5:**
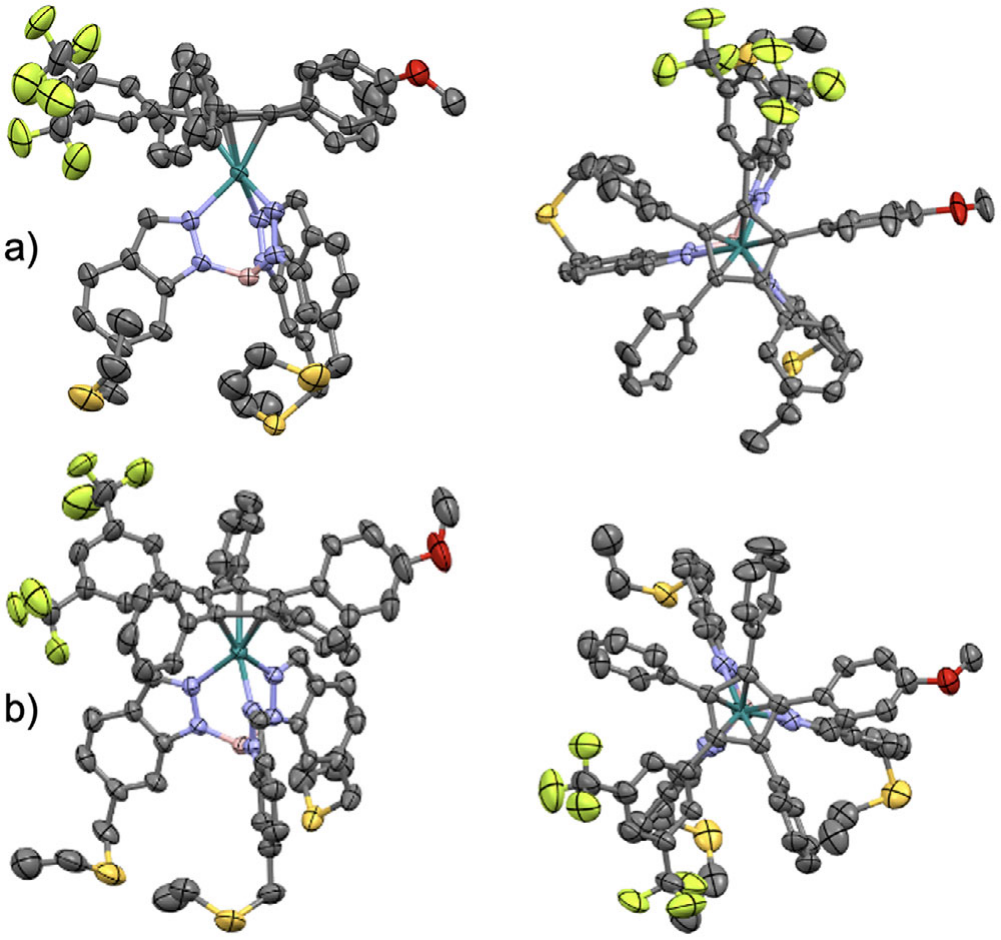
Side view (left) and top view (right) of the molecular structure of the ruthenium complexes a) **1,2‐Cp^Ar5^[Ru]Tp** (CCDC‐2443081) and b) **1,3‐Cp^Ar5^[Ru]Tp** (CCDC‐2443082).^[^ ^24^ ^]^ Thermal ellipsoids are drawn at 30% probability. Hydrogen atoms and solvent molecules are omitted for clarity.

Both ruthenium(II) complexes were characterized by single‐crystal X‐ray diffraction analysis. Single crystals suitable for analysis were obtained by slow evaporation of a 1:1 dichloromethane/methanol solution of the complex (Figure 5). The X‐ray structure is similar to previous structures obtained for this family of Ru(II) complexes featuring a pentaarylcyclopentadienyl ligand combined with the same hydrotrisindazolylborate ligand,^[^ ^25^ ^]^ with the latter binding in a facial tripodal mode (i.e., κ^3^‐*N*,*N*“,*N*”). Both complexes have a piano stool structure with the Cp substituents fitting in the vacant spaces of the tripodal ligand. The distances between the centroid of the Cp ring and the ruthenium center were measured as 1.793 Å for **1,2‐Cp^Ar5^[Ru]Tp** and 1.797 Å for **1,3‐Cp^Ar5^[Ru]Tp**. These values are nearly identical to the Cp–Ru bond distance (1.798 Å) previously reported for the complex incorporating a nonfunctionalized pentaphenylcyclopentadienyl ligand.^[^ ^25^ ^]^ Since both enantiomers are present in the unit cell, it appeared that the crystallization occurred without spontaneous resolution.

### 2.6 Calculation of Dipole Moment in the 1,2 and 1,3‐functionalized Ruthenium Complexes

To evaluate the dipole moment in the ruthenium complexes, we exploited the X‐ray crystallographic structures of both compounds. All types of disorder were first removed, and the resulting geometries were used to calculate the dipole moments. The calculations were performed using the BP86 DFT functional with the def2‐TZVP basis set. For the ruthenium atom, a specific ZORA‐def2‐TZVP basis set was employed instead of an ECP approximation. The dipole moment was calculated to be 5.60 D for **1,2‐Cp^Ar5^[Ru]Tp** and 4.58 D for **1,3‐Cp^Ar5^[Ru]Tp**. These results follow the same trend as the calculated properties of the isolated **1,2‐Cp^Ar5^** and **1,3‐Cp^Ar5^** anionic ligands. However, a significant increase in the dipole moment is observed upon complexation, the anionic character of the ligand being drastically reduced by its coordination with the Ru(II) trisindazolylborate fragment. In consequence, the involvement in Ru–Cp bonding of the previously nonbonding lone pair on the Cp anion reduces its electron shielding effect, thus enhancing the dipole moment in the ruthenium complexes.

## 3 Conclusion

Two novel molecular motor candidates that integrate both planar chirality and a permanent dipole into their architecture have been synthesized and characterized, including by X‐ray crystallography. By incorporating electron‐donating and electron‐withdrawing substituents into dissymmetrically substituted cyclopentadienyl ligands, two ruthenium(II) complexes (**1,2‐Cp^Ar5^[Ru]Tp** and **1,3‐Cp^Ar5^[Ru]Tp**) were successfully synthesized with dipoles of 5.60 and 4.58 D, respectively. Computational and experimental analyses confirmed the enhanced dipole moments upon metal complexation, validating the impact of both ligand geometry and coordination on electronic properties.

These findings pave the way for more sophisticated molecular motors which can be addressed with an electric field. STM studies are now underway to explore the controllability of these motors under an electric field, as well as the role of chirality on the unidirectional rotation.

## 4 Experimental Section

### General methods

All chemicals and solvents were purchased from commercial suppliers and were used without further purification. Anhydrous solvents, 4‐ethynylanisole, copper(II) bromide, sodium sulfate, 3,5‐bis(trifluoromethyl)bromobenzene, triethylamine, copper(I) iodide, sodium thiosulfate, palladium(II) diacetate, diphenylacetone, acetic anhydride, tetrabutylammonium hydroxide (1 M in methanol), ammonium chloride, iodine, dichlorobis(triphenylphosphine)palladium(II), thionyl chloride, phenyllithium (1.8 M in hexane), *n*‐butyllithium (2.5 M in hexane), ammonium chloride, triruthenium dodecacarbonyl, phenylacetylene, and 4‐bromoanisole were purchased from Merck. Pyridine was purchased from Alfa Aesar. The thallium salt of hydrotris{6‐[(ethylsulfanyl)methyl]indazol‐1‐yl}borate was synthesized as previously reported.^[^ ^22^ ^]^ All reactions were carried out using standard Schlenk techniques under an argon atmosphere. A glove box was used for the last synthetic step leading to the target compounds (coordination of the tripodal ligand). Column chromatography was carried out on 230–400 mesh silica gel (Aldrich) unless otherwise stated. Thin layer chromatography (TLC) was performed on pre‐coated aluminum‐backed silica gel 60 UV254 plates (Macherey–Nagel) with visualization effected using ultraviolet irradiation (λ = 254, 366 nm). NMR and mass spectra were recorded by the appropriate services of the Toulouse Institute of Chemistry (ICT – UAR 2599). ^1^H, ^19^F, and ^13^C NMR spectra were recorded on Avance 300 MHz (probe 5mm BBO BB‐1H Z‐GRD), Bruker Avance III HD 500 MHz (cryoprobe Prodigy 5mm BBO, 1H ATMA) and Avance 500 MHz (cryoprobe 5mm ^1^H, ^13^C). Residual solvent signals were used as internal reference for ^1^H, ^19^F, and ^13^C NMR spectra. Chemical shifts (δ) are reported in ppm. Coupling constants (*J*) are given in Hz and the following abbreviations have been used to describe the signals: singlet (s); broad singlet (br. s); doublet (d); triplet (t); quadruplet (q); septet (sep), multiplet (m). Full assignments of ^1^H and ^13^C NMR spectra were made with the assistance of COSY, HMBC and HSQC spectra. The numbering system used for the assignment of signals in new compounds is provided in the Supporting Information section, along with the corresponding spectra. High‐resolution mass spectra (HR‐MS) were performed with a Waters GCT Premier spectrometer for desorption chemical ionization (DCI‐CH_4_), a Waters Xevo G2 QTOF spectrometer for electrospray ionization (ESI) and with a Waters MALDI micro MX spectrometer for matrix assisted laser desorption ionization (MALDI) (matrix: *trans*‐2‐[3‐(4‐*tert*‐butylphenyl)‐2‐methyl‐2‐propenylidene]malono‐nitrile DTCB; λ = 337 nm). UV/Vis spectra were recorded with a Varian Cary 5000 spectrometer, ε [mol^−1^dm^3^cm^−1^] is reported in parentheses. IR spectra were recorded with a PerkinElmer Spectrum 100 FT‐IR. Only selected characteristic peaks are reported. Melting points were measured with a Krüss M5000 melting‐point apparatus and are uncorrected.

### 1‐(3′,5′‐bis(trifluoromethyl)phenyl)‐2‐(4′‐methoxyphenyl)ethyne (2)

[adapted from ref. 12b]

In a dry Schlenk tube were added 4‐ethynylanisole (0.49 mL, 3.78 mmol, 1.2 eq), 3,5‐bis(trifluoromethyl)bromobenzene (0.54 mL, 3.15 mmol, 1.0 eq), dichlorobis(triphenylphosphine)palladium(II) (66 mg, 95 µmol, 3 mol%), dry and degassed triethylamine (1.6 mL, 11.7 mmol, 3.7 eq), and dry and degassed tetrahydrofuran (6.4 mL). Finally, copper(I) iodide (18 mg, 95 µmol, 3 mol%) was added and the mixture was refluxed for 16 hours. After cooling down, a saturated solution of ammonium chloride (25 mL) was added and the reaction medium was extracted with ethyl acetate (20 mL). The organic phase was washed three times with brine, then dried over sodium sulfate and the solvent was removed under reduced pressure. The crude mixture was purified by column chromatography (SiO_2_, Hexane) to give **2** (1085 mg, 3.15 mmol, > 99%) as a white solid. Rf = 0.4 (SiO_2_, Hexane); ^1^H‐NMR (300 MHz, CDCl_3_, 20 °C): δ 7.92 (br. s, 2H, H_o_), 7.78 (br. s, 1H, H_p_), 7.49 (AA'BB’, 2H, ^3^ *J* = 9.2 Hz, ^4^ *J* = 2.2 Hz, H_b_), 6.91 (AA'BB’, 2H, ^3^ *J* = 8.8 Hz, ^4^ *J* = 2.2 Hz, H_a_), 3.84 (s, 3H, CH_3_). The ^1^H‐NMR spectrum data match those reported in the literature.^[^ ^12b^ ^]^

### 1‐(3′,5′‐bis(trifluoromethyl)phenyl)‐2‐(4′‐methoxyphenyl)ethane‐1,2‐dione (3)

[adapted from ref. 14]

In a dry Schlenk tube were added **2** (200 mg, 0.58 mmol, 1.0 eq), palladium(II) diacetate (13 mg, 58 µmol, 0.1 eq), copper(II) bromide (13 mg, 58 µmol, 0.1 eq) and dry dimethylsulfoxide (4 mL). The reaction mixture was stirred at 120 °C overnight. After cooling down, ethyl acetate (10 mL) was added and the organic phase was washed five times with brine, dried over sodium sulfate and the solvents were removed under reduced pressure. The crude mixture was purified by column chromatography (SiO_2_, Hexane/Chloroform 80:20) to afford **3** (153 mg, 0.41 mmol, 71%) as a greenish solid. Rf = 0.35 (SiO_2,_ Hexane/Chloroform 80:20,); m.p. 102 °C; ^1^H‐NMR (300 MHz, CDCl_3_, 20 °C): δ 8.44 (br. s, 2H, H_o_), 8.13 (br. s, 1H, H_p_), 7.99 (AA'BB’, 2H, ^3^ *J* = 9.2 Hz, ^4^ *J* = 2.5 Hz, H_b_), 7.02 (AA'BB’, 2H, ^3^ *J* = 8.8 Hz, ^4^ *J* = 2.5 Hz, H_a_), 3.92 (s, 3H, CH_3_); ^13^C{^1^H} NMR (75 MHz, CDCl_3_, 20 °C): δ 190.9 (C_7_), 190.6 (C_6_), 165.8 (C_2_), 134.9 (C_5_), 132.9 (q, ^2^ *J* (C‐F) = 29.6 Hz, C_10_),133.0 (C_4_), 130.0 (q, ^3^ *J* (C‐F) = 3.7 Hz, C_9_), 127.8 (sep, ^3^ *J* (C‐F) = 3.7 Hz, C_11_), 125.3 (C_8_), 122.8 (q, ^1^ *J* (C‐F) = 273.1 Hz, C_12_), 114.8 (C_3_), 55.9 (C_1_); ^19^F{^1^H}‐NMR (282 MHz, CDCl_3_, 20 °C): δ ‐63.29; IR (KBr, cm^−1^): 1681 (CO); 1655 (CO); HR‐MS (DCI‐CH_4_): calcd. for C_17_H_11_F_6_O_3_ [M + H]^+^: 377.0612, found 377.0610.

### (*Z*)‐2,3‐bis(3′,5′‐bis(trifluoromethyl)phenyl)‐1,4‐bis(4′‐methoxyphenyl)but‐2‐ene‐1,4‐dione (3′)

[adapted from ref. 13]

In a dry Schlenk tube were introduced **2** (625 mg, 1.8 mmol, 1 eq), iodine (509 mg, 2.0 mmol, 1.1 eq) and DMSO (10.4 mL). The mixture was stirred and heated at 150 °C for 17 h. After cooling down the reaction mixture, 20 mL of a 1% sodium thiosulfate aqueous solution were poured, resulting in a precipitation. The solid was filtered under vacuum, washed with water and dried under vacuum. The resulting powder was purified by column chromatography (SiO_2_, Hexane/Chloroform 70:30) to give **3′** (366 mg, 0.53 mmol, 28%) as a white solid. Rf = 0.25 (SiO_2_, Hexane/Chloroform 70:30); m.p. 173 °C; ^1^H‐NMR (300 MHz, CDCl_3_, 20 °C): δ 7.82 (AA'BB’,4H, ^3^ *J* = 6.8 Hz, ^4^ *J* = 2.1 Hz, H_b_), 7.75 (br. s, 4H, H_o_), 7.65 (br.s, 2H, H_p_), 6.89 (AA'BB’, 4H, ^3^ *J* = 6.8 Hz, ^4^ *J* = 2.1 Hz, H_a_), 3.91 (s, 6H, CH_3_); ^13^C{^1^H}‐NMR (75 MHz, CDCl_3_, 20 °C): δ 193.0 (C_6_), 164.8 (C_2_), 141.8 (C_7_), 136.7 (C_8_), 132.9 (C_4_), 132.8 (q, ^2^ *J* (C‐F) = 33.0 Hz, C_10_), 129.6 (q, ^3^ *J* (C‐F) = 2.6 Hz C_9_), 128.1 (C_5_), 122.5 (sep, ^3^ *J* (C‐F) = 3.6 Hz, C_11_) 122.7 (q, ^1^ *J* (C‐F) = 277.2 Hz, C_12_), 114.3 (C_3_), 55.7 (C_1_); ^19^F{^1^H}‐NMR (282 MHz, CDCl_3_, 20 °C): δ ‐63.30; IR (KBr, cm^−1^): 1658 (CO); HR‐MS (MALDI): calcd. for C_34_H_20_F_12_O_4_ [M + Na]^+^: 743.1068, found 743.1062; Crystallographic data: CCDC‐2393052.^[^ ^24^ ^]^

### 3‐[3′,5′‐bis(trifluoromethyl)phenyl]‐4‐(4′‐methoxyphenyl)‐2,5‐diphenylcyclopenta‐2,4‐dien‐1‐one (4)

In a dry Schlenk tube, compound **3** (100 mg, 0.27 mmol, 1 eq), diphenylacetone (80 µL, 0.4 mmol, 1.5 eq) and *tert*‐butanol (0.8 mL) were added. A solution of tetrabutylammonium hydroxide (1 M in methanol, 53 µL, 53 µmol, 0.2 eq) was added dropwise under reflux, until the solution turned red. The mixture was stirred for 1 hour. After cooling down, ethyl acetate (5 mL) and water (5 mL) were added. The organic phase was then washed three times with brine, dried over sodium sulfate and the solvents were removed under reduced pressure. The crude was then dissolved in acetic anhydride (0.3 mL), and a catalytic amount of concentrated sulfuric acid was added dropwise, causing the solution to turn to deep red. The mixture was stirred at room temperature for 30 min, then quenched with water. Dichloromethane (10 mL) was added, and the aqueous phase was extracted three times with dichloromethane. The combined organic phases were dried over sodium sulfate, and the solvents were removed under reduced pressure. The crude mixture was purified by column chromatography (SiO_2_, Hexane/Dichloromethane 80:20) to give **4** (53 mg, 0.10 mmol, 36%) as a dark red oily liquid. Rf = 0.3 (SiO_2_, Hexane/Dichloromethane 70:30); ^1^H‐NMR (300 MHz, CD_2_Cl_2_, 20 °C): δ 7.78 (br. s, 1H, H_p_), 7.36 (br. s, 2H, H_o_), 7.32‐7.25 (m, 8H, H_9,9′,10,10′_), 7.20‐7.17 (m, 2H, H_11_,_11′_), 6.83 (AA'BB’, 2H, ^3^ *J* = 9.0 Hz, H_b_), 6.75 (AA'BB’, 2H, ^3^ *J* = 9.0 Hz, H_a_) 3.76 (s, 3H, CH_3_); ^13^C{^1^H}‐NMR (75 MHz, CDCl_3_, 20 °C): δ 199.5 (C_12_), 160.5 (C_2_), 153.7 (C_6_), 150.2 (C_13_),135.3 (C_8′_),131.1 (q, ^2^ *J* (C‐F) = 29.6 Hz, C_16_), 130.9 (C_4_), 130.9, 130.7, 130.2, 130.1, 129.6, 128.6 (q, ^3^ *J* (C‐F) = 3.7 Hz, C_15_), 127.82 (C_10_ or C_10′_), 127.76 (C_10_ or C_10′_), 124.9 (C_7_), 122.3 (q, ^1^ *J* (C‐F) = 272.7 Hz, C_18_), 124.4 (C_5_), 122.0 (sep, ^3^ *J* (C‐F) = 3.7 Hz, C_17_), 114.1 (C_3_), 55.5 (C_1_). Signals in the 130.9‐129.6 ppm region, corresponding to C_7′_, C_8_, C_9_, C_11_, C_9′_, C_11′_ and C_14_ could not be precisely assigned; ^19^F{^1^H}‐NMR (282 MHz, CD_2_Cl_2_, 20 °C): δ ‐63.65; IR (KBr, cm^−1^): 1709 (CO); HR‐MS (DCI‐CH_4_): calcd. for C_32_H_23_F_6_O_2_ [M + H_2 _+ H]^+^: 553.1602, found 553.1606.

### 3‐[3′,5′‐bis(trifluoromethyl)phenyl]‐4‐(4′‐methoxyphenyl)‐1,2,5‐triphenylcyclopenta‐2,4‐dien‐1‐ol (1,2‐Cp^Ar5^‐OH)

In a dry Schlenk tube, cyclopentadienone **4** (95 mg, 0.16 mmol, 1.0 eq) and dry tetrahydrofuran (2.1 mL) were added and the resulting solution was cooled down at ‐78 °C. Under stirring, a solution of phenyllithium (1.8 M in hexane, 185 µL, 0.34 mmol, 2.1 eq) was added dropwise. After 30 min, the reaction media was quenched by adding a saturated solution of ammonium chloride (10 mL). Dichloromethane (15 mL) was then added, and the aqueous phase extracted three times with dichloromethane. The combined organic phases were washed two times with brine, then dried over sodium sulfate and the solvents were removed under reduced pressure. The crude mixture was purified by column chromatography (SiO_2_, Hexane/Dichloromethane gradient 80:20 to 60:40) to give **1,2‐Cp^Ar5^‐OH** (53 mg, 0.083 mmol, 52%) as a yellow solid. Rf = 0.4 (SiO_2_, Hexane/Dichloromethane 80:20); m.p. 77 °C; ^1^H‐NMR (300 MHz, CDCl_3_, 20 °C): δ 7.63 (br. s, 1H, H_p_), 7.54 (dd, 2H, ^3^ *J* = 8.7 Hz, ^4^ *J* = 1.5 Hz, H_9″_), 7.32 (br. s, 2H, H_o_), 7.29‐7.26 (m, 2H, H_10″_), 7.24‐7.21 (m, 1H, H_11″_), 7.14‐7.05 (m, 8H, H_9,10_, H_9′,10′_), 6.94‐6.88 (m, 4H, H_11_, H_11′_, H_b_), 6.75 (AA'BB’, 2H, ^3^ *J* = 9.0 Hz, H_a_), 3.76 (s, 3H, CH_3_), 2.51 (s, 1H, OH); ^13^C{^1^H}‐NMR (75 MHz, CDCl_3_, 20 °C): δ 159.4 (C_2_), 151.4 (C_12_), 147.5 (C_6_), 141.0 (C_8″_), 139.5 (C_8′_ or C_8_), 139.3 (C_8′_ or C_8_), 137.1 (C_7″_), 133.6 (C_7_ or C_7′_), 133.0 (C_7_ or C_7′_), 131.2 (C_4_), 130.8 (q, ^2^ *J* (C‐F) = 32.9 Hz, C_15_),130.8 (C_13_), 130.5 (q, ^3^ *J* (C‐F) = 3.1 Hz, C_14_), 129.6, 129.5, 128.8, 128.3, 128.10, 128.05, 127.4, 126.6 (C_5_), 125.2 (C_9″_), 123.2 (q, ^1^ *J* (C‐F) = 269.1 Hz, C_17_), 120.6 (sep, ^3^ *J* (C‐F) = 4.0 Hz, C_16_), 114.2 (C_3_), 90.4 (C_7″_), 55.4 (C_1_). Signals in the 129.6‐127.4 ppm region, corresponding to C_9_, C_10_, C_11_, C_9′_, C_10′_, C_11′_, C_10″_ and C_11″_ could not be precisely assigned; ^19^F{^1^H}‐NMR (282 MHz, CDCl_3_, 20 °C): δ ‐63.20; HR‐MS (EI): calcd. for C_38_H_27_FO_2_ [M + H]^+^: 629.1915, found 629.1501.

### Chloridodicarbonyl‐η^5^‐1‐(4′‐methoxyphenyl)‐2‐[3′,5′‐bis(trifluoromethyl)phenyl]‐3,4,5‐triphenylcyclopentadienyl ruthenium(II) (1,2‐Cp^Ar5^[Ru]Cl(CO)_2_)

In a dry Schlenk tube, 1,2‐Cp^Ar5^‐OH (50 mg, 80 µmol, 1 eq), dry pyridine (37 µL, 0.46 mmol, 6 eq) and dry diethylether (6 mL) were added, and the reaction mixture was cooled to ‐10 °C. Thionyl chloride (34 µL, 0.46 mmol, 6 eq) was added dropwise under stirring. The mixture was stirred for 1 hour while allowing to come back to room temperature, and then quenched with a diluted hydrochloric acid solution (1 M, 15 mL). Diethylether was added (10 mL) and the organic phase was washed three times with brine and then with water. The organic phase was dried over sodium sulfate and the solvents were removed under reduced pressure. The formation of the chlorinated intermediate 1,2‐Cp^Ar5^‐Cl was confirmed by HR‐MS (MALDI: calcd. for C_38_H_25_ClF_6_O [M]^+^: 646.1498, found 646.1493), however due to its instability, the crude product was directly engaged in the next step. The crude mixture was added to a dry Schlenk tube. Triruthenium dodecacarbonyl (29 mg, 46 µmol, 0.6 eq) and dry toluene (4.6 mL) were added. The resulting mixture was heated at 110 °C for 1 h. The solvents were then removed *in vacuo*. The crude mixture was adsorbed on silica and purified by column chromatography (SiO_2_, Hexane/Dichloromethane gradient 70:30 to 40:60) to give 1,2‐Cp^Ar5^[Ru]Cl(CO)_2_ (33 mg, 41 µmol, 51% over two steps) as a green solid. Rf = 0.5 (SiO_2_, Hexane/Dichloromethane 40:60); ^1^H‐NMR (300 MHz, CD_2_Cl_2_, 20 °C): δ 7.72 (br. s, 1H, H_p_), 7.43 (br. s, 2H, H_o_), 7.27‐7.01 (m, 15H, H_9,9′,9″_, H_10,10′,10″_, H_11,11′,11″_), 6.96 (AA'BB’, 2H, ^3^ *J* *=* 9.3 Hz, ^4^ *J* = 2.2 Hz, H_b_), 6.70 (AA'BB’, 2H, ^3^ *J* *= *9.3 Hz, ^4^ *J* *= *2.4 Hz, H_a_), 3.75 (s, 3H, CH_3_); ^13^C{^1^H}‐NMR (75 MHz, CD_2_Cl_2_, 20 °C): δ 197.02 (CO), 196.96 (CO), 160.6 (C_2_), 133.8 (C_4_), 132.9 (C_13_), 132.7 (q, ^3^ *J* (C‐F) = 2.9 Hz, C_14_), 132.54, 132.47, 130.7 (q, ^2^ *J* (C‐F) = 33.3 Hz, C_15_), 129.8, 129.5, 129.3, 129.1, 128.7, 128.4, 128.3, 122.2 (q, ^1^ *J* (C‐F) = 273.2 Hz, C_17_), 122.2 (sep, ^3^ *J* (C‐F) = 3.8 Hz, C_16_), 120.7 (C_5_), 114.1 (C_3_), 108.0 (C_7_ or C_7′_ or C_7″_), 107.0 (C_12_), 106.4 (C_6_), 105.6 (C_7_ or C_7′_ or C_7″_), 104.2 (C_7_ or C_7′_ or C_7″_), 55.7 (C_1_). Signals in the 132.5‐128.3 ppm region, corresponding to C_8_, C_9_, C_10_, C_11_, C_8‵_, C_9‵_, C_10‵_, C_11‵_, C_8″_, C_9″_, C_10″_, C_11″_, could not be precisely assigned; ^19^F{^1^H}‐NMR (282 MHz, CD_2_Cl_2_, 20 °C): δ ‐63.35; IR (KBr, cm^−1^): 2047 (CO), 1990 (CO); HR‐MS (ESI): calcd. for C_40_H_29_ClF_6_NO_3_Ru [M + NH_4_]^+^: 822.0792, found 822.0806.

### η^5^‐1‐(4′‐methoxyphenyl)‐2‐[3′,5′‐bis(trifluoromethyl)phenyl]‐3,4,5‐triphenylcyclopentadienyl hydrotris{6′‐[(ethylsulfanyl)methyl]indazol‐1′‐yl}borate ruthenium(II) (1,2‐Cp^Ar5^[Ru]Tp)

In a glovebox, 1,2‐Cp^Ar5^[Ru]Cl(CO)_2_ (33 mg, 41 µmol, 1 eq), thallium hydrotris{6‐[(ethylsulfanyl)methyl]indazol‐1‐yl}borate (65 mg, 82 µmol, 2 eq), and dry acetonitrile (1.4 mL) were placed in an oven dried tube for microwave synthesis containing a magnetic stir bar. The vessel was sealed and heated using microwave irradiation (115 °C, pressure up to 5 bar, available power of 250 W, 3 × 10 min). The resulting suspension was filtered through a pad of silica and eluted with dichloromethane. The solvents were removed under reduced pressure and the crude mixture was purified by column chromatography (SiO_2_, Hexane/Dichloromethane gradient 90:10 to 70:30) to give 1,2‐Cp^Ar5^[Ru]Tp (22 mg, 17 µmol, 41%) as an orange solid. Rf = 0.4 (SiO_2_, Hexane/Dichloromethane 40:60); ^1^H‐NMR (500 MHz, CD_2_Cl_2_, 20 °C): δ 7.90 (br. s, 3H, H_1_), 7.82 (br. s, 3H, H_6_), 7.77 (br. s, 2H, H_o_), 7.64 (br. s, 1H, H_p_), 7.46‐7.38 (m, 6H, H_25,25′,25″_), 7.35 (AA'BB’, 2H, ^3^ *J* = 8.5 Hz, H_b_), 7.29 (AA'BB’, 3H, ^3^ *J* = 7.5 Hz, H_4_), 7.17‐7.14 (m, 3H, H_27,27′,27″_), 7.09‐7.01 (m, 9H, H_26,26′,26″_, H_3_), 6.61 (AA'BB’, 2H, ^3^ *J* *=* 8.8 Hz, H_a_), 3.90 (s, 6H, H_8_), 3.67 (s, 3H, H_22_), 2.47 (q, 6H, ^3^ *J* = 6.0 Hz, H_9_), 1.28 (t, 9H, ^3^ *J *= 7.0 Hz, H_10_); B‐H was not observed. ^13^C{^1^H}‐NMR (125 MHz, CD_2_Cl_2_): δ 159.6 (C_21_), 144.0 (C_7_), 140.7 (C_6_), 138.0 (C_5_), 137.7 (C_12_), 135.3 (C_19_), 134.2, 134,1, 134.0, 133.9 (q, ^3^ *J* (C‐F) = 3.7 Hz, C_13_), 133.7, 133.6, 133.5, 130.3 (q, ^2^ *J* (C‐F) = 32.5 Hz, C_14_), 128.1, 128.0, 127.8, 127.6, 127.5, 123.5 (q, ^1^ *J* (C‐F) = 269.1 Hz, C_16_), 125.1 (C_18_), 122.6 (C_3_), 122.5 (C_2_), 120.5 (sep, ^3^ *J* (C‐F) = 4.4 Hz, C_15_), 119.9 (C_4_), 113.5 (C_20_), 111.4 (C_1_), 90.7 (C_23_ or C_23′_ or C_23″_), 89.8 (C_23_ or C_23′_ or C_23″_), 89.5 (C_11_), 85.5 (C_17_), 84.9 (C_23_ or C_23′_ or C_23″_), 55.5 (C_22_), 36.9 (C_8_), 25.7 (C_9_), 14.7 (C_10_). Signals in the 134.2‐133.5 ppm region corresponding to C_24_, C_24‵_, C_24″_, C_25_, C_25‵_ and C_25″_, and signals in the 128.1‐127.5 ppm region corresponding to C_26_, C_26‵_, C_26″_, C_27_, C_27‵_ and C_27″_, could not be precisely assigned; ^19^F{^1^H}‐NMR (282 MHz, CD_2_Cl_2_, 20 °C): δ ‐63.49; UV: λ_max_ (ε) = 300 (18200 mol^−1^ dm^3^ cm^−1^); HR‐MS (ESI): calcd. for C_68_H_60_BF_6_N_6_ORuS_3_ [M + H]^+^: 1299.3055 found 1299.3093; Crystallographic data: CCDC‐2443081.^[^ ^24^ ^]^

### 1‐(3′,5′‐bis(trifluoromethyl)phenyl)‐2‐phenylethyne (5)

[adapted from ref. 23]

In a dry Schlenk tube were added phenylacetylene (0.14 mL, 1.27 mmol, 1.3 eq), 3,5‐bis(trifluoromethyl)bromobenzene (0.17 mL, 0.98 mmol, 1.0 eq) and dichlorobis(triphenylphosphine)palladium(II) (21 mg, 29 µmol, 3 mol%). Dry degassed tetrahydrofuran (2 mL) and degassed triethylamine (0.5 mL, 3.6 mmol, 3.7 eq) were then added. Finally copper(I) iodide (6 mg, 29 µmol, 3 mol%) was added and the mixture was refluxed for 16 h. After cooling down, a saturated solution of ammonium chloride (10 mL) was added and the reaction medium was extracted with ethyl acetate (15 mL). The organic phase was washed four times with an aqueous solution of ammonium chloride, followed by brine and dried over sodium sulfate. Solvent was then removed under reduced pressure and the crude product was purified by column chromatography (SiO_2_, Hexane 100%) to give **5** (307 mg, 0.98 mmol, 99%) as a white solid. Rf = 0.7 (SiO_2_, Hexane 100%); ^1^H‐NMR (300 MHz, CDCl_3_, 20 °C): δ 7.95 (br. s, 2H, H_o_), 7.81 (br. s, 1H, H_p_), 7.57‐7.54 (m, 2H, H_a_), 7.40‐7.38 (m, 3H, H_b,c_). The ^1^H‐NMR spectrum data match those reported in the literature.^[^ ^23^ ^]^

### 1‐(3′,5′‐bis(trifluoromethyl)phenyl)‐2‐phenylethane‐1,2‐dione (6)

[adapted from ref. 13]

In a dry Schlenk tube, **5** (100 mg, 0.32 mmol, 1.0 eq), palladium(II) diacetate (7 mg, 32 µmol, 0.1 eq), copper(II) bromide (7 mg, 32 µmol, 0.1 eq) and dry dimethylsulfoxide (2.3 mL) were added. The mixture was heated at 120 °C overnight under stirring. After cooling down, ethyl acetate (15 mL) and brine (10 mL) were added. The organic phase was washed five times with brine, dried over sodium sulfate, and the solvent was removed under reduced pressure. The crude mixture was purified by column chromatography (SiO_2_, Hexane/Dichloromethane 95:5) to give compound **6** (78 mg, 0.23 mmol, 71%) as a yellow oil. Rf = 0.35 (SiO_2_, Hexane/Dichloromethane 95:5); ^1^H‐NMR (300 MHz, CDCl_3_, 20 °C): δ 8.45 (br. s, 2H, H_o_), 8.15 (br. s, 1H, H_p_), 8.03‐8.00 (m, 2H, H_3_), 7.75‐7.70 (m, 1H, H_1_), 7.59‐7.54 (m, 2H, H_2_); ^13^C{^1^H}‐NMR (75 MHz, CDCl_3_, 20 °C): δ 192.1 (C_6_), 190.5 (C_5_), 135.8 (C_7_), 134.7 (C_4_), 132.7 (q, ^2^ *J* (C‐F) = 33.8 Hz, C_9_), 133.4 (C_2_), 130.4 (C_3_), 130.0 (q, ^3^ *J* (C‐F) = 3.7 Hz, C_8_), 129.4 (C_1_), 127.9 (sep, ^3^ *J* (C‐F) = 3.7 Hz, C_10_), 122.8 (q, ^1^ *J* (C‐F) = 273.5 Hz, C_11_); ^19^F{^1^H}‐NMR (282 MHz, CDCl_3_, 20 °C): δ ‐63.04; IR (KBr, cm^−1^): 1694 (CO), 1677 (CO); HR‐MS (MALDI): calcd. for C_16_H_7_F_6_O_2_ [M‐H]^−^: 345.0345, found 345.0345.

### 3‐[3′,5′‐bis(trifluoromethyl)phenyl]‐2,4,5‐triphenylcyclopenta‐2,4‐dien‐1‐one (7)

In a dry Schlenk tube were successively added **6** (70 mg, 0.19 mmol, 1 eq), diphenylacetone (60 µL, 0.29 mmol, 1.5 eq), and *tert*‐butanol (0.9 mL). A solution of tetrabutylammonium hydroxide (1 M in methanol, 38 µL, 38 µmol, 0.2 eq) was added dropwise under reflux, causing the reaction mixture to turn red. The mixture was stirred for 1 hour. After cooling down, water (20 mL) and dichloromethane (20 mL) were added. The organic phase was washed several times with brine, dried over sodium sulfate, and the solvent was removed under reduced pressure. The crude compound was dissolved in acetic anhydride (0.22 mL) and a catalytic amount of concentrated sulfuric acid was added, causing the reaction to turn dark pink. The reaction mixture was then stirred for 30 min at room temperature and quenched with water. The crude compound was extracted with dichloromethane (3 × 15 mL), dried over sodium sulfate and the solvent removed under reduced pressure. The crude mixture was purified by column chromatography (SiO_2_, Hexane/Dichloromethane gradient 90:10 to 80:20) to give the cyclopentadienone **7** (50 mg, 96 µmol, 50%) as a dark pink solid. Rf = 0.4 (SiO_2_, Hexane/Dichloromethane 80:20); m.p. 135 °C; ^1^H‐NMR (300 MHz, CD_2_Cl_2_, 20 °C): δ 7.77 (br. s, 1H, H_p_), 7.35‐7.21 (m, 15H, H_o_, H_9,9′_, H_10,10′,10″_, H_11,11′,11″_), 6.95 (dd, 2H, ^3^ *J* = 8.1 Hz, ^4^ *J* = 1.2 Hz, H_9″_); ^13^C{^1^H}‐NMR (75 MHz, CDCl_3_, 20 °C): δ 199.9 (C_12_), 154.3 (C_6_), 151.0 (C_7″_), 135.5 (C_8′_), 132.5 (C_8_), 131.1 (q, ^2^ *J* (C‐F) = 33.8 Hz, C_2_), 130.8 (C_7_), 130.52, 130.45, 130.3 (q, ^3^ *J* (C‐F) = 3.8 Hz, C_4_), 130.1, 129.5, 129.4, 128.9, 128.81, 128.79, 128.5, 128.2, 127.9, 126.1 (C_7′_), 122.4 (sep, ^3^ *J* (C‐F) = 3.9 Hz, C_1_), 123.4 (q,^1^ *J* (C‐F) = 274.6 Hz, C_3_). Signals in the 130.5‐129.4 ppm region corresponding to C_5_, C_8″_, C_9_, C_9‵_ and C_9″_, and signals in the 128.9‐127.9 ppm region corresponding to C_10_, C_10‵_, C_10″_, C_11_, C_11‵_ and C_11″_, could not be precisely assigned; ^19^F{^1^H}‐NMR (297 MHz, CD_2_Cl_2_, 25°C): δ ‐63.36; IR (KBr, cm^−1^): 1711 (CO); HR‐MS (MALDI): calcd. for C_31_H_19_F_6_O [M + H]^+^: 521.1340, found 521.1335.

### 3‐[3′,5′‐bis(trifluoromethyl)phenyl]‐1‐(4′‐methoxyphenyl)‐2,4,5‐triphenylcyclopenta‐2,4‐dien‐1‐ol (1,3‐Cp^Ar5^‐OH)

In an oven‐dried Schlenk tube, 4‐bromoanisole (24 µL, 0.19 mmol, 2 eq) was dissolved in dry diethyl ether (0.4 mL), and cooled down to ‐78 °C. While carefully maintaining this temperature, *n*‐butyllithium (2.5 M in hexane, 75 µL, 0.19 mmol, 2 eq) was added dropwise. The reaction was stirred at ‐78 °C for 30 min and allowed to come back to room temperature, resulting in the formation of the 4‐methoxyphenyllithium solution. At ‐78 °C, this solution was added via cannula into a Schlenk tube containing **7** (50 mg, 96 µmol, 1 eq) dissolved in dry tetrahydrofuran (1 mL). The reaction mixture was stirred for 30 min and then quenched with a saturated solution of ammonium chloride (5 mL), causing the solution to turn pale yellow. Aqueous phase was extracted with dichloromethane (3 × 10 mL) and the combined organic phases were washed with brine (2 × 20 mL), dried over sodium sulfate, and the solvent was evaporated under reduced pressure. The crude mixture was purified by column chromatography (SiO_2_, Hexane/Dichloromethane gradient 80:20 to 60:40) to give **1,3‐Cp^Ar5^‐OH** (30 mg, 48 µmol, 50%) as a yellow solid. Rf = 0.28 (SiO_2_, Hexane/Dichloromethane 60:40); m.p. 75 °C; ^1^H‐NMR (300 MHz, CDCl_3_, 20 °C): δ 7.60 (br. s, 1H, H_p_), 7.46 (AA'BB’, 2H, ^3^ *J* = 8.7 Hz, H_b_), 7.28 (br. s, 2H, H_o_), 7.23‐6.91 (m, 15H, H_9,9′,9″_, H_10,10′,10″_, H_11,11′,11″_), 6.83 (AA'BB’, 2H, ^3^ *J* = 8.7 Hz, ^4^ *J* = 2.2 Hz, H_a_), 3.77 (s, 3H, CH_3_), 2.48 (s, 1H, OH); ^13^C{^1^H}‐NMR (75 MHz, CDCl_3_, 20 °C): δ 158.9 (C_16_), 151.4 (C_6_), 147.9 (C_7″_), 141.1 (C_7_′), 138.9 (C_7_), 137.0 (C_5_), 134.6 (C_8′_), 133.4 (C_8_), 133.1 (C_8″_), 131.0 (q, ^2^ *J* (C‐F) = 33.8 Hz, C_2_), 130.4 (q, ^3^ *J* (C‐F) = 3.6 Hz, C_4_), 129.8 (C_9_ or C_9′_ or C_9″_), 129.7 (C_9_ or C_9′_ or C_9″_), 129.6 (C_9_ or C_9′_ or C_9″_), 128.6, 128.3, 128.1, 127.9, 127.5, 126.4 (C_13_), 123.2 (q, ^1^ *J* (C‐F) = 274.2 Hz, C_3_), 120.6 (sep, ^3^ *J* (C‐F) = 3.9 Hz, C_1_), 114.2 (C_15_), 90.3 (C_12_), 55.3 (C_17_). Signals in the 128.6‐127.5 ppm region, corresponding to C_10_, C_10′_, C_10″_, C_11_, C_11′_, C_11″_ and C_14_, could not be precisely assigned; ^19^F{^1^H}‐NMR (282 MHz, CDCl_3_, 20 °C): δ ‐63.27; HR‐MS (MALDI): calcd. for C_38_H_26_F_6_O_2_ [M]^+^: 628.1837, found 628.1825.

### Chloridodicarbonyl‐η^5^‐1‐(4′‐methoxyphenyl)‐3‐[3′,5′‐bis(trifluoromethyl)phenyl]‐2,4,5‐triphenylcyclopentadienyl ruthenium(II) (1,3‐Cp^Ar5^[Ru]Cl(CO)_2_)

In a dry Schlenk tube, 1,3‐Cp^Ar5^‐OH (100 mg, 0.16 mmol, 1 eq), dry pyridine (74 µL, 0.92 mmol, 6 eq), and dry diethylether (12 mL) were added and the reaction mixture was cooled down to ‐10 °C. Under stirring, thionyl chloride (68 µL, 0.92 mmol, 6 eq) was added dropwise. The mixture was stirred for 1 hour while allowing to come back to room temperature, and then quenched with a diluted hydrochloric acid solution HCl (1 M, 15 mL). Diethylether was added (20 mL), and the organic phase was washed three times with brine followed by water. The organic phase was dried over sodium sulfate and the solvent was removed under reduced pressure. The formation of the chlorinated intermediate 1,3‐Cp^Ar5^‐Cl was confirmed by HR‐MS (MALDI: calcd. for C_38_H_25_ClF_6_O [M]^+^: 646.1498, found 646.1496), however due to its instability, the crude was directly engaged in the next step. The crude mixture was added to a dry Schlenk tube. Triruthenium dodecacarbonyl (58 mg, 92 µmol, 0.6 eq) and dry toluene (9.2 mL) were added. The resulting mixture was heated at 110 °C for 1 h. The solvents were then removed *in vacuo*. The crude mixture was adsorbed on silica and purified by column chromatography (SiO_2_, Hexane/Dichloromethane gradient 80:20 to 40:60) to give 1,3‐Cp^Ar5^[Ru]Cl(CO)_2_ (70 mg, 87 µmol, 54% over two steps) as a green solid. Rf = 0.45 (SiO_2_, Hexane/Dichloromethane 40:60); ^1^H‐NMR (300 MHz, CD_2_Cl_2_, 20 °C): δ 7.71 (br. s, 1H, H_p_), 7.38 (br. s, 2H, H_o_), 7.32‐7.07 (m, 15H, H_9,9′,9″_, H_10,10′,10″_, H_11,11′,11″_), 6.94 (AA'BB’, 2H, ^3^ *J* = 8.7 Hz, ^4^ *J* = 2.2 Hz, H_b_), 6.63 (AA'BB’, 2H, ^3^ *J* = 8.7 Hz, ^4^ *J* = 2.2 Hz, H_a_), 3.73 (s, 3H, CH_3_); ^13^C{^1^H}‐NMR (75 MHz, CD_2_Cl_2_): δ 197.04 (CO), 197.03 (CO), 160.3 (C_16_), 133.7 (C_14_), 132.7, 132.63, 132.56, 130.9 (q, ^2^ *J* (C‐F) = 33.8 Hz, C_2_), 129.8, 129.6, 129.32, 129.26, 129.0, 128.9, 128.8, 128.7, 128.5, 123.3 (q, ^1^ *J* (C‐F) = 259.6 Hz, C_3_), 122.2 (sep, ^3^ *J* (C‐F) = 3.7 Hz, C_1_), 121.0 (C_13_), 113.7 (C_15_), 108.6 (C_6_), 107.0 (C_12_), 106.4 (C_7_ or C_7′_ or C_7″_), 105.3 (C_7_ or C_7′_ or C_7″_), 104.8 (C_7_ or C_7′_ or C_7″_), 55.6 (C_17_). Signals in the 132.7‐132.5 ppm region corresponding to C_5_, C_9_, C_9‵_, C_9″_, C_10_, C_10‵_, and C_10″_, and signals in the 129.8‐128.5 ppm region corresponding to C_4_, C_8_, C_8‵_, C_8″_, C_11_, C_11‵_ and C_11″_, could not be precisely assigned; ^19^F{^1^H}‐NMR (282 MHz, CD_2_Cl_2_, 20 °C): δ ‐63.75; IR (KBr, cm^−1^): 2047 (CO), 1998 (CO); HR‐MS (ESI): calcd. for C_41_H_26_ClF_6_O_5_Ru [M + COO]^−^: 849.0425, found 849.0424.

### η^5^‐1‐(4′‐methoxyphenyl)‐3‐[3′,5′‐bis(trifluoromethyl)phenyl]‐2,4,5‐triphenylcyclopentadienyl hydrotris{6′‐[(ethylsulfanyl)methyl]indazol‐1′‐yl}borate ruthenium(II) (1,3‐Cp^Ar5^[Ru]Tp)

In a glovebox, 1,3‐Cp^Ar5^[Ru]Cl(CO)_2_ (57 mg, 71 µmol, 1 eq), dry acetonitrile (2.3 mL) and thallium hydrotris{6‐[(ethylsulfanyl)methyl]indazol‐1‐yl}borate (112 mg, 140 µmol, 2 eq) were placed in an oven dried tube for microwave synthesis containing a magnetic stir bar. The vessel was sealed and heated using microwave irradiation (115 °C, pressure up to 5 bar, available power of 250 W, 3 × 10 minutes). The resulting suspension was filtered through a pad of silica and eluted with dichloromethane. The solvent was removed under reduced pressure, and the crude mixture was purified by column chromatography (SiO_2_, Hexane/Dichloromethane gradient 90:10 to 70:30) to give 1,3‐Cp^Ar5^[Ru]Tp (53 mg, 41 µmol, 58%) as an orange solid. Rf = 0.35 (SiO_2_, Hexane/Dichloromethane 40:60); ^1^H‐NMR (500 MHz, CD_2_Cl_2_, 20 °C): δ 7.93 (br. s, 3H, H_1_), 7.85 (br. s, 3H, H_6_), 7.78 (br. s, 2H, H_o_), 7.65 (br. s, 1H, H_p_), 7.50‐7.45 (m, 6H, H_19,19′,19″_), 7.32 (m, 5H, H_4_, H_b_), 7.19 (m, 3H, H_21,21′,21″_), 7.12‐7.07 (m, 6H, H_20,20′,20″_), 7.04 (AA'BB’, 3H, ^3^ *J* = 8.4 Hz, H_3_), 6.59 (AA'BB’, 2H, ^3^ *J* = 9.4 Hz, H_a_), 3.97 (s, 6H, H_8_), 3.69 (s, 3H, H_27_), 2.50 (q, 6H, ^3^ *J* = 7.4 Hz, H_9_), 1.30 (t, 9H, ^3^ *J* = 7.4 Hz, H_10_); B‐H was not observed. ^13^C{^1^H}‐NMR (125 MHz, CD_2_Cl_2_): δ 159.4 (C_26_), 144.1 (C_7_), 140.7 (C_6_), 138.0 (C_5_), 137.5 (C_12_), 135.1 (C_24_), 134.2, 134.1, 134.0, 133.8, 133.6, 133.8 (q, ^3^ *J* (C‐F) = 3.3 Hz, C_13_), 130.6 (q, ^2^ *J* (C‐F) = 32.5 Hz, C_14_), 128.2, 128.1, 127.8, 127.7, 124.9 (C_23_), 124.6 (q, ^1^ *J* (C‐F) = 272.9 Hz, C_15_), 122.6 (C_3_), 122.5 (C_2_), 120.9 (sep, ^3^ *J* (C‐F) = 3.7 Hz, C_16_), 120.3 (C_4_), 113.0 (C_25_), 111.4 (C_1_), 91.5 (C_22_), 90.1 (C_11_), 89.1 (C_17_ or C_17′_ or C_17″_), 85.6 (C_17_ or C_17′_ or C_17″_), 84.0 (C_17_ or C_17_′ or C_17″_), 55.4 (C_27_), 36.9 (C_8_), 25.7 (C_9_), 14.8 (C_10_). Signals in the 134.2‐133.6 ppm region corresponding to C_18_, C_18‵_, C_18″_, C_19_, C_19‵_, and C_19″_, and signals in the 128.2‐127.7 ppm region corresponding to C_20_, C_20‵_, C_20″_, C_21_, C_21‵_ and C_21″_, could not be precisely assigned; ^19^F{^1^H}‐NMR (282 MHz, CD_2_Cl_2_, 20 °C): δ ‐63.49; UV: λ_max_ (ε) = 300 (18400 mol^−1^ dm^3^ cm^−1^); HR‐MS (ESI): calcd. for C_68_H_60_BF_6_N_6_ORuS_3_ [M + H]^+^: 1299.3055, found 1299.3053; Crystallographic data: CCDC‐2443082.^[^ ^24^ ^]^

## Supporting Information

Electronic Supplementary Information (ESI) available with ^1^H‐NMR, ^13^C‐NMR, ^19^F‐NMR, HR‐MS, DFT calculations and X‐ray crystallographic data.

## Conflict of Interest

The authors declare no conflict of interest.

## Supporting information



Supporting Information

Supporting Information

## Data Availability

The data that support the findings of this study are available in the supplementary material of this article.
