# Supplementary material for: Dipolar Molecular Motor Candidates with Planar Chirality
Source: Chemistry. 2025 Sep 29;32(7):e02277. doi: 10.1002/chem.202502277 (PMC12910426; doi:10.1002/chem.202502277)
Supplement: Supplementary file 1 — Supporting Information [file CHEM-32-e02277-s002.pdf]

Supporting Information

Dipolar Molecular Motors with Planar Chirality

Thomas A. Hector,[a] Shohei Katao,[b] Nathalie Saffon-Merceron,[c] Valentin Magné,[a] Claire Kammerer,[a] and Gwénaël Rapenne*[a,b]

[a] CEMES, Université de Toulouse, CNRS, 29, rue Jeanne Marvig, 31055 Toulouse, Cedex 4, France
[b] Division of Materials Science, Nara Institute of Science and Technology, 8916-5 Takayama, Ikoma, 630-0192, Japan
[c] Université de Toulouse, Institut de Chimie de Toulouse, ICT UAR 2599, 118 route de Narbonne, 31062 Toulouse, France

Table of contents

I. NMR spectra **S2**

Compound **2** S2
Compound **3** S3
Compound **3'** S5
Compound **4** S7
Compound **1,2-Cp^{Ar5}-OH** S9
Compound **1,2-Cp^{Ar5}[Ru]Cl(CO)$_2$** S11
Compound **1,2-Cp^{Ar5}[Ru]Tp** S13
Compound **5** S15
Compound **6** S16
Compound **7** S18
Compound **1,3-Cp^{Ar5}-OH** S20
Compound **1,3-Cp^{Ar5}[Ru]Cl(CO)$_2$** S22
Compound **1,3-Cp^{Ar5}[Ru]Tp** S24

II. HR-MS data **S26**

Compound **3** S26
Compound **3'** S26
Compound **4** S27
Compound **1,2-Cp^{Ar5}-OH** S27
Compound **1,2-Cp^{Ar5}[Ru]Cl(CO)$_2$** S28
Compound **1,2-Cp^{Ar5}[Ru]Tp** S29
Compound **6** S30
Compound **7** S31
Compound **1,3-Cp^{Ar5}-OH** S32
Compound **1,3-Cp^{Ar5}[Ru]Cl(CO)$_2$** S33
Compound **1,3-Cp^{Ar5}[Ru]Tp** S34

III. Crystallographic data **S35**

Compound **3'** S35
Compound **1,2-Cp^{Ar5}[Ru]Tp** S37
Compound **1,3-Cp^{Ar5}[Ru]Tp** S39
Tables with bond lengths and angles S42

IV. DFT calculations **S49**

1. Optimized coordinates and associated vibrational frequencies of the **1,2-Cp^{Ar5}** anion S49
2. Optimized coordinates and associated vibrational frequencies of the **1,3-Cp^{Ar5}** anion S55
3. Optimized coordinates and associated vibrational frequencies of the **3'-E** isomer S61
4. Optimized coordinates and associated vibrational frequencies of the **3'-Z** isomer S67
5. Electron density map of the **1,2-Cp^{Ar5}** and **1,3-Cp^{Ar5}**anions S73

V. References **S74**

I. NMR spectra

Compound 2

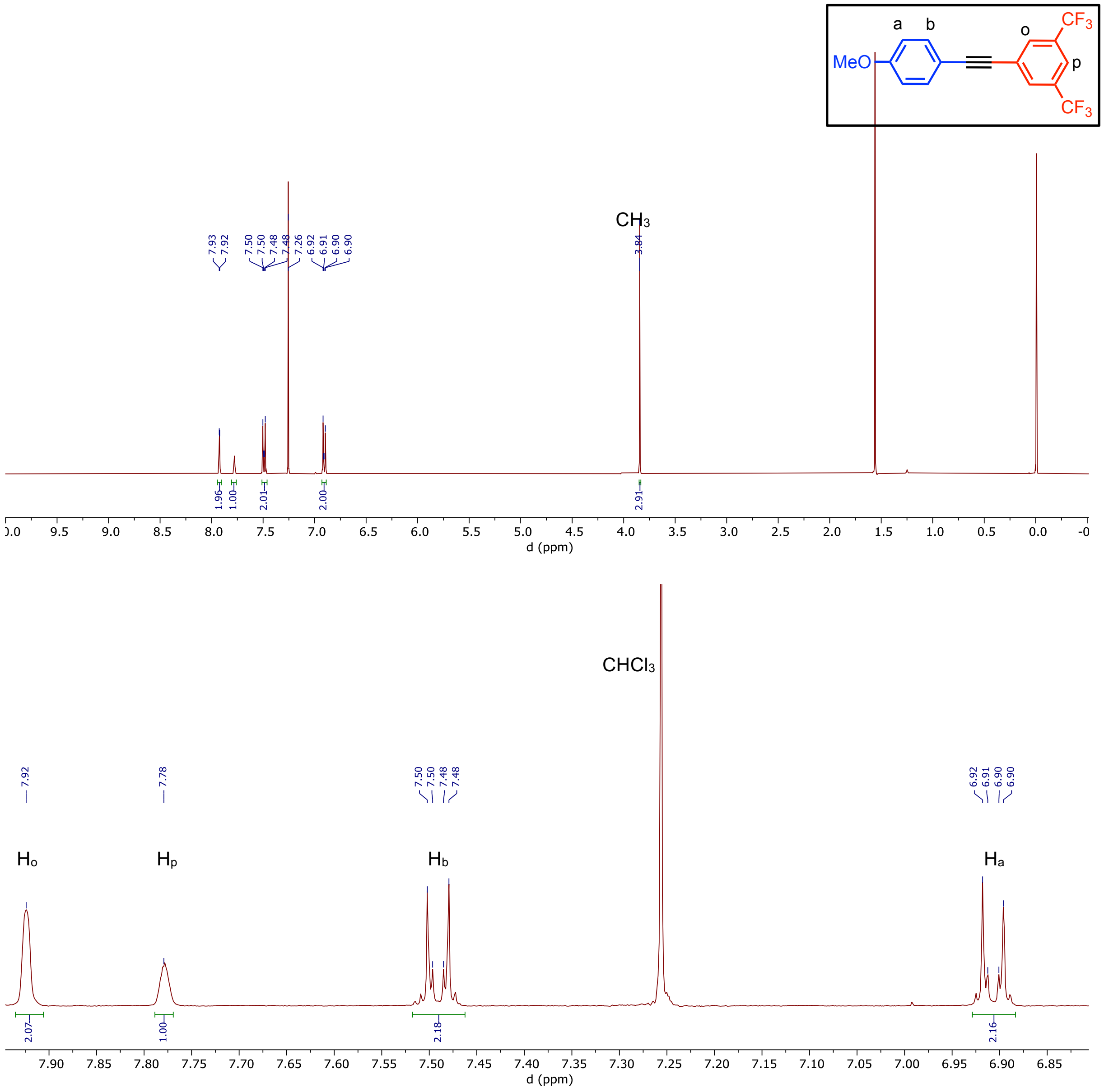

Figure S1. ^{1}H-NMR spectrum of **2** in $CDCl_3$ at 293 K, 300 MHz.

Compound 3

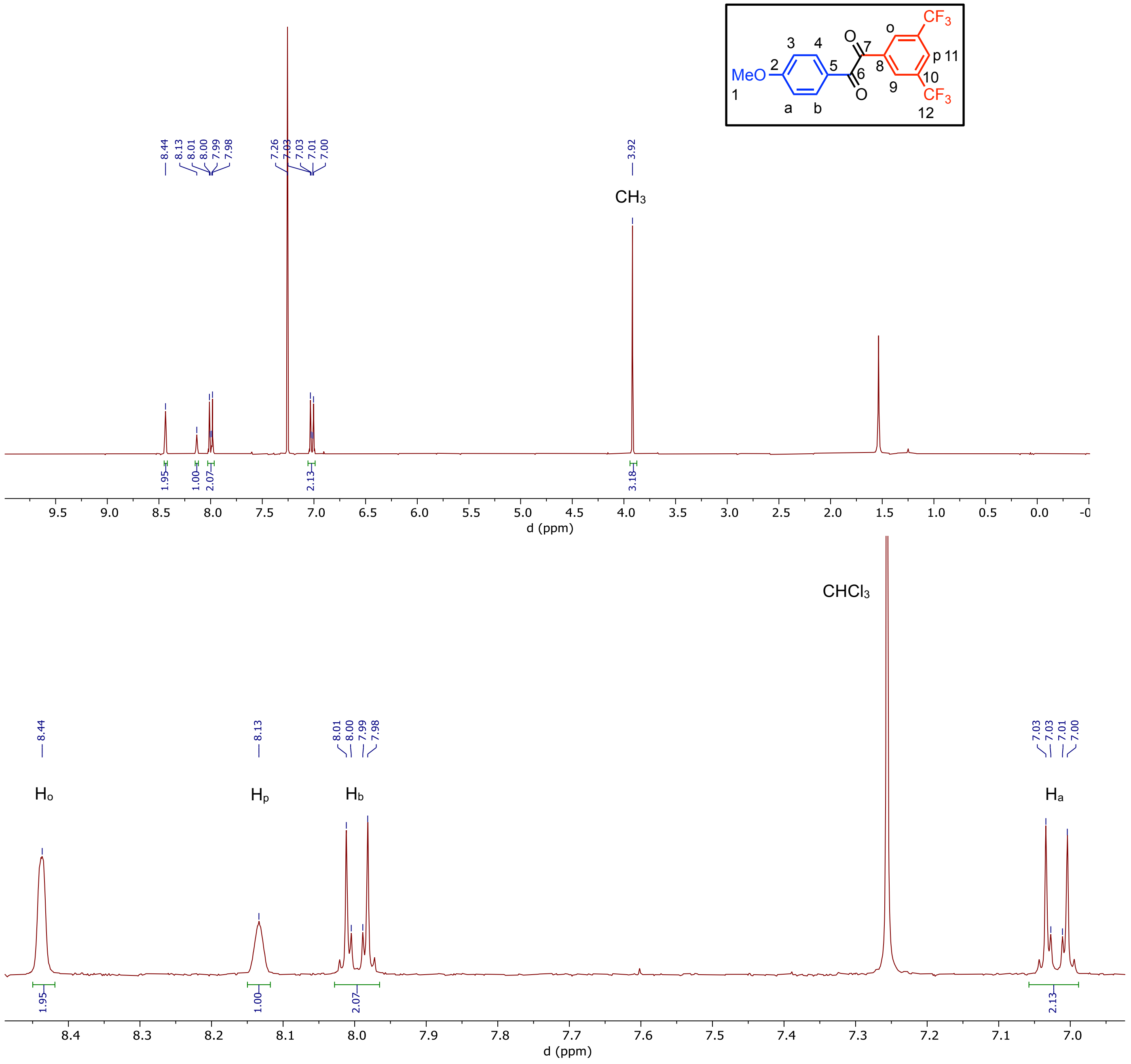

Figure S2. ^{1}H-NMR spectrum of **3** in $CDCl_3$ at 293 K, 300 MHz.

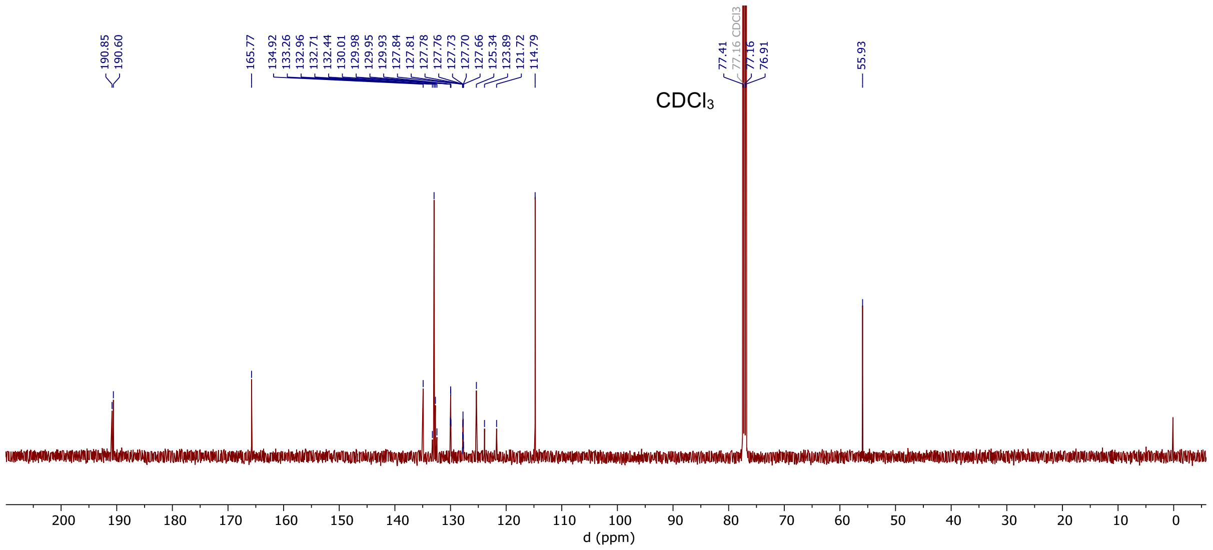

Figure S3. $^{13}C\{^{1}H\}$-NMR spectrum of **3** in $CDCl_3$ at 293 K, 75 MHz.

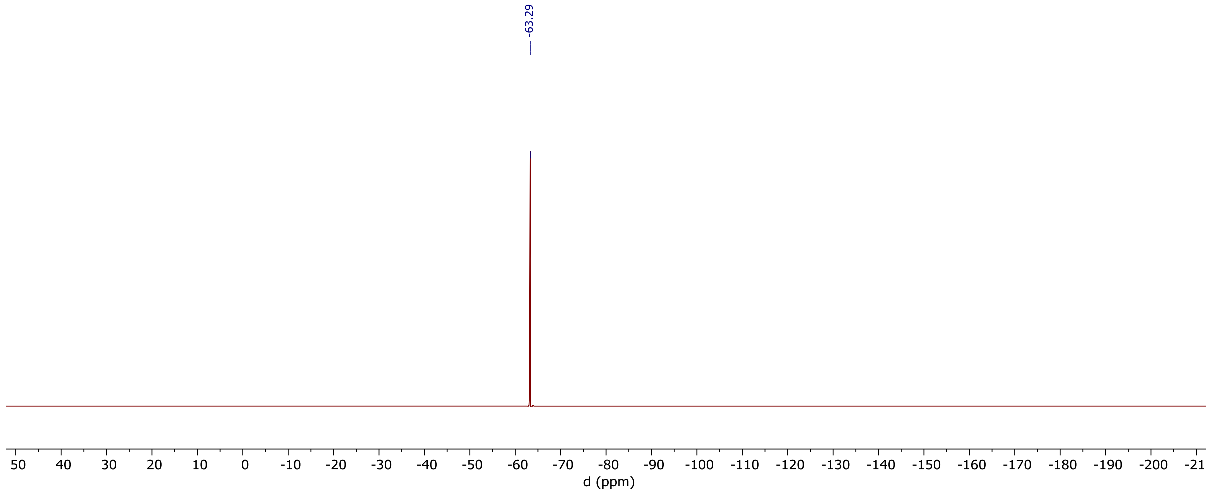

Figure S4. $^{19}F\{^{1}H\}$-NMR spectrum of **3** in $CDCl_3$ at 293 K, 282 MHz.

Compound 3’

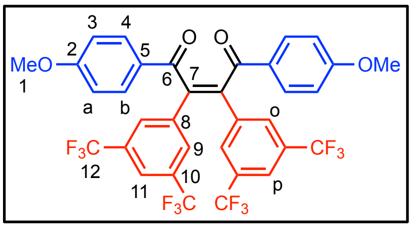

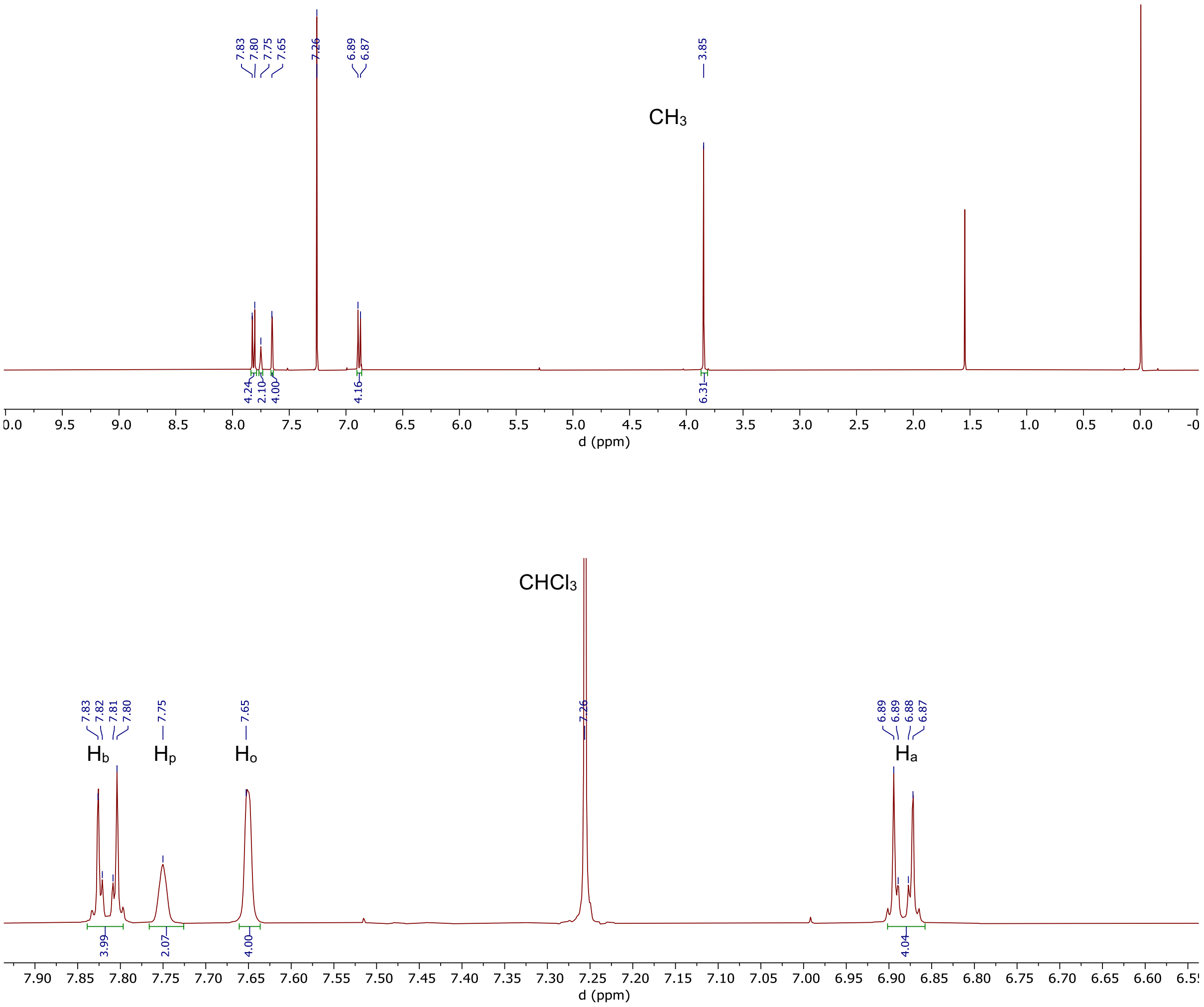

Figure S5. ^{1}H-NMR spectrum of **3’** in $CDCl_3$ at 293 K, 300 MHz.

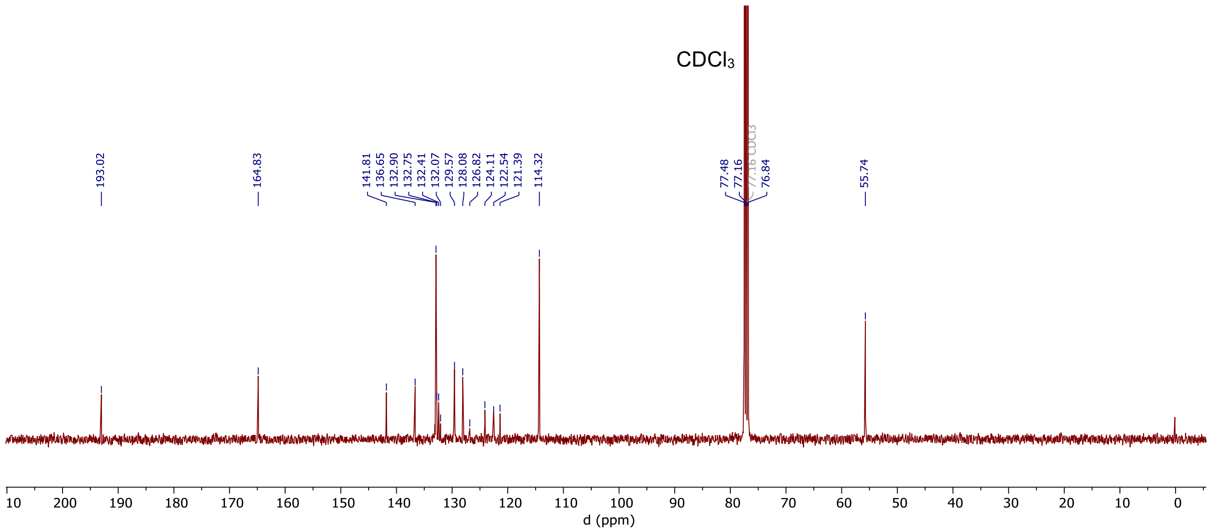

Figure S6. $^{13}C\{^1H\}$-NMR spectrum of **3’** in $CDCl_3$ at 293 K, 75 MHz.

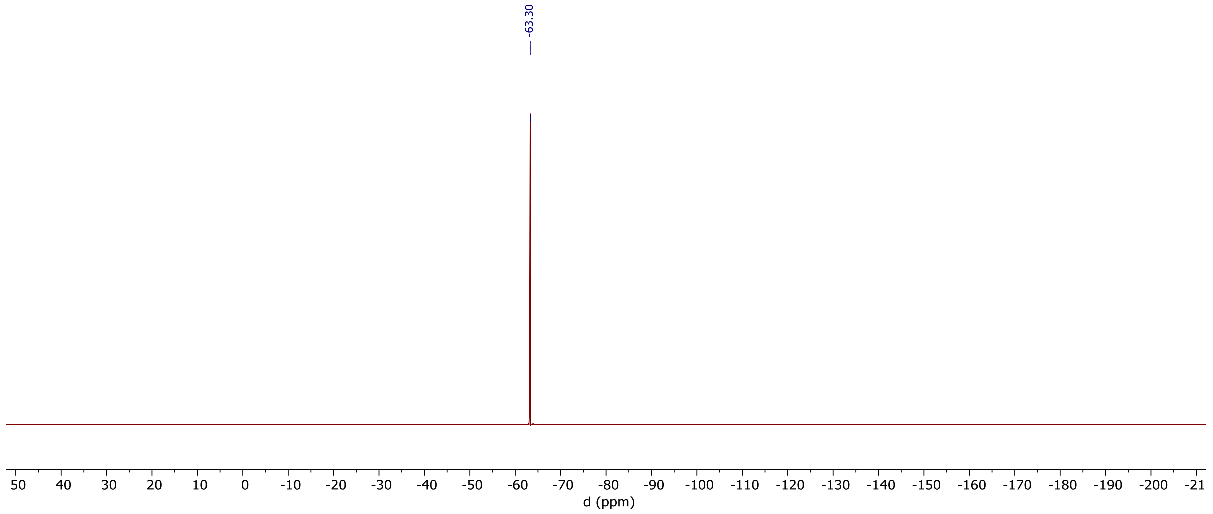

Figure S7. $^{19}F\{^1H\}$-NMR spectrum of **3’** in $CDCl_3$ at 293 K, 282 MHz.

Compound 4

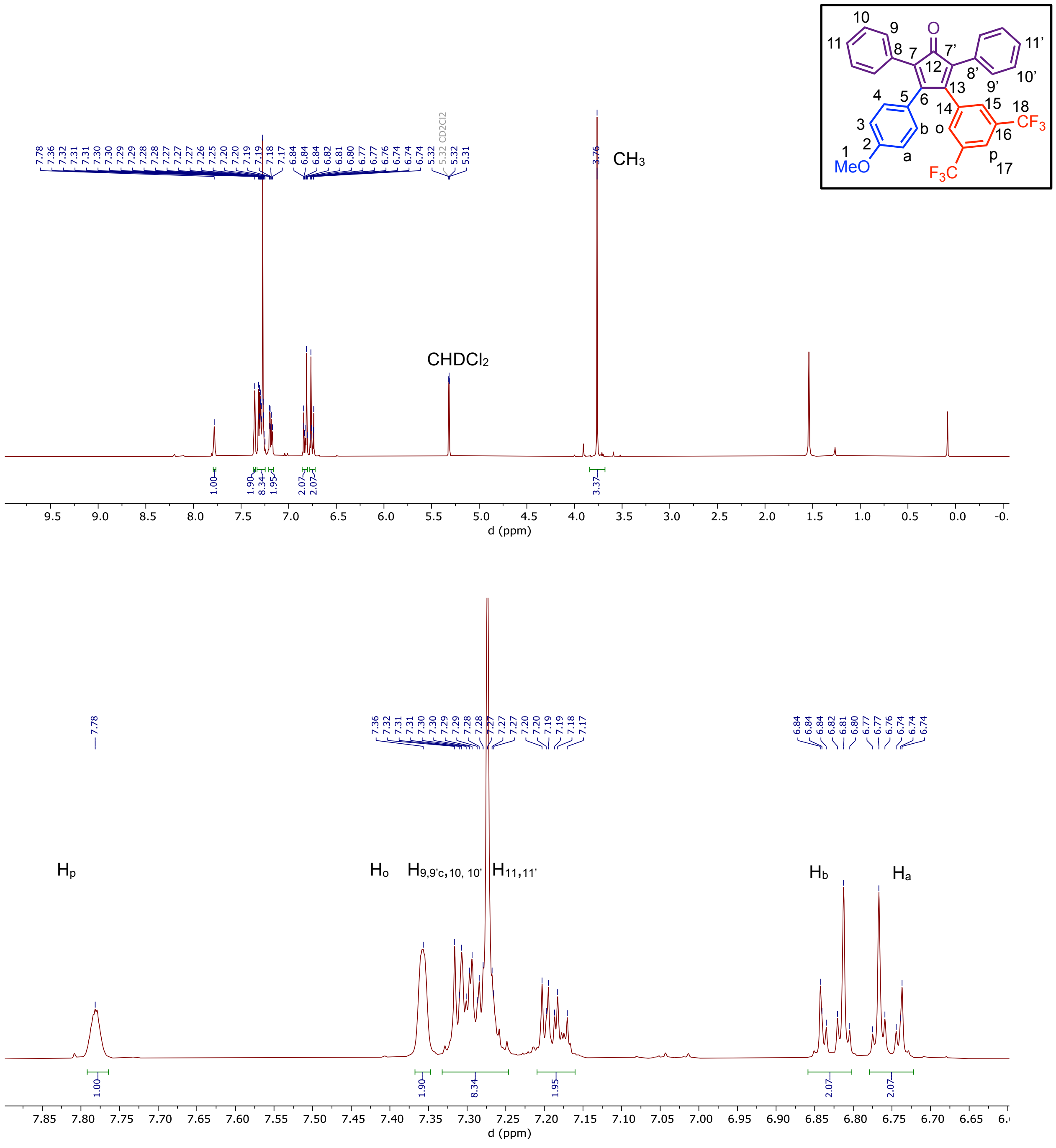

Figure S8. ^{1}H-NMR spectrum of **4** in CD_2Cl_2 at 293 K, 300 MHz.

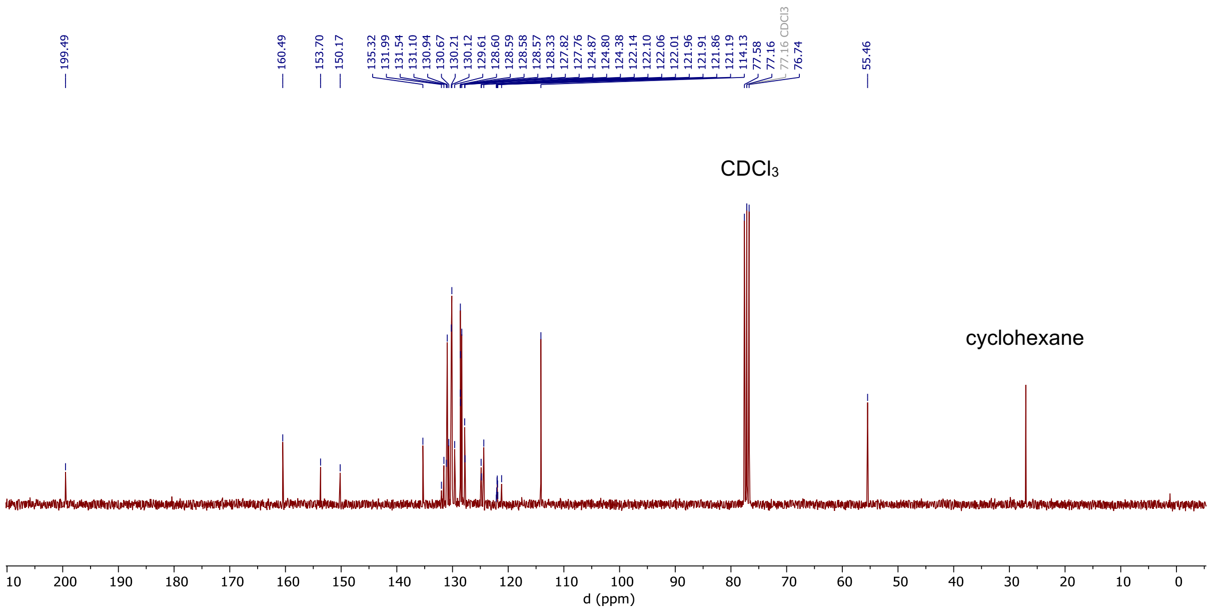

Figure S9. $^{13}C\{^{1}H\}$-NMR spectrum of **4** in $CDCl_3$ at 293 K, 75 MHz.

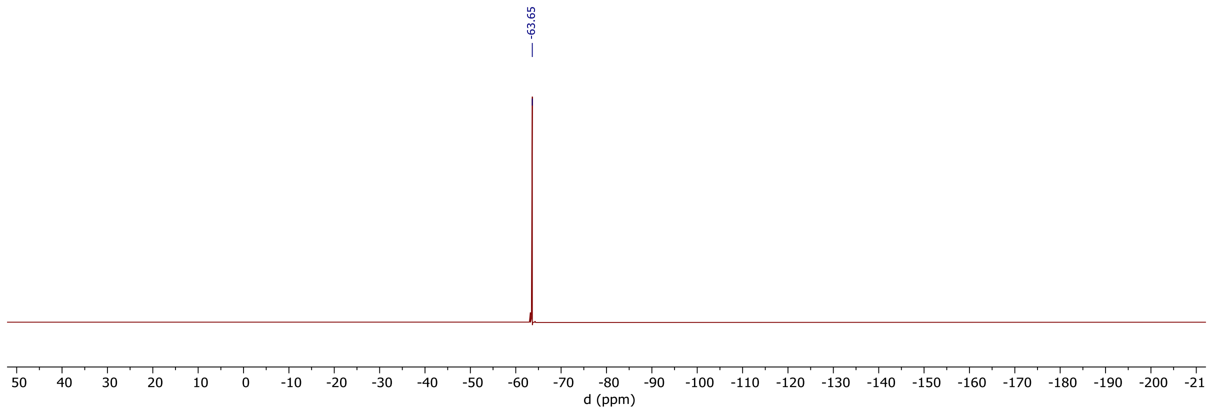

Figure S10. $^{19}F\{^{1}H\}$-NMR spectrum of **4** in CD_2Cl_2 at 293 K, 282 MHz.

Compound 1,2-Cp^{Ar5}-OH

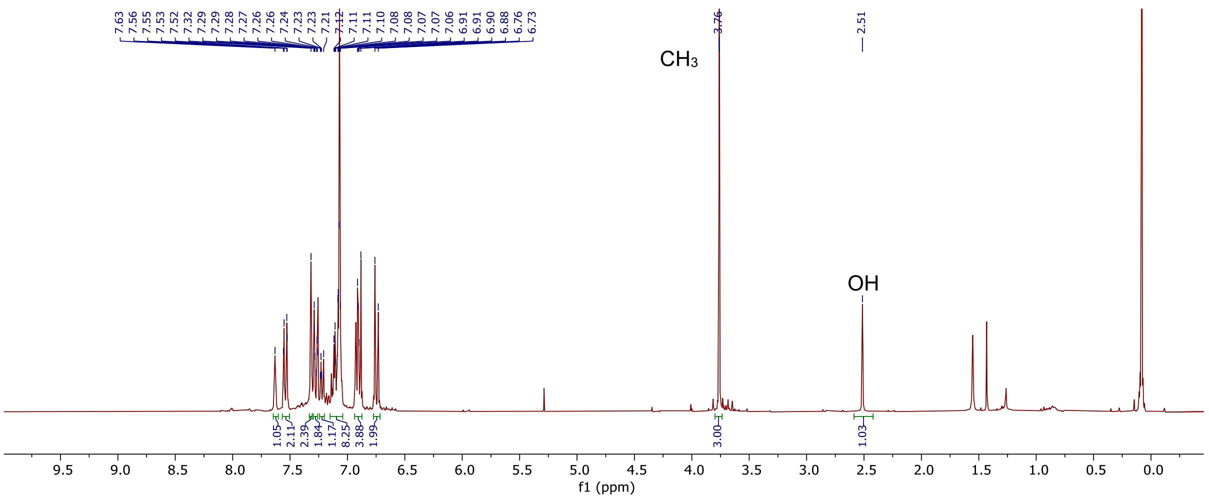

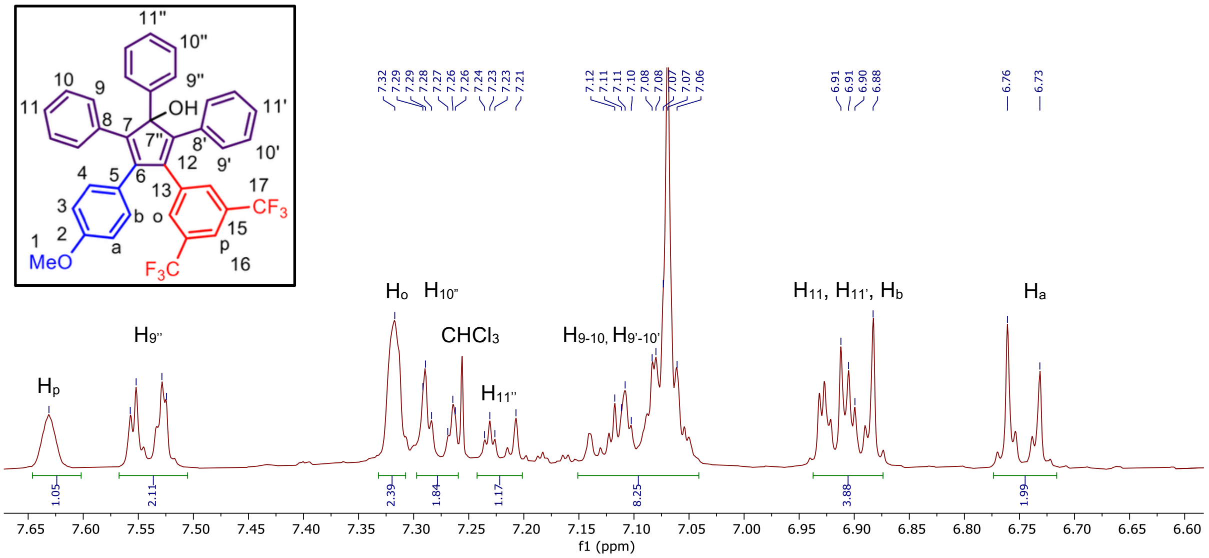

Figure S11. ^{1}H-NMR spectrum of **1,2-Cp^{Ar5}-OH** in $CDCl_3$ at 293 K, 300 MHz.

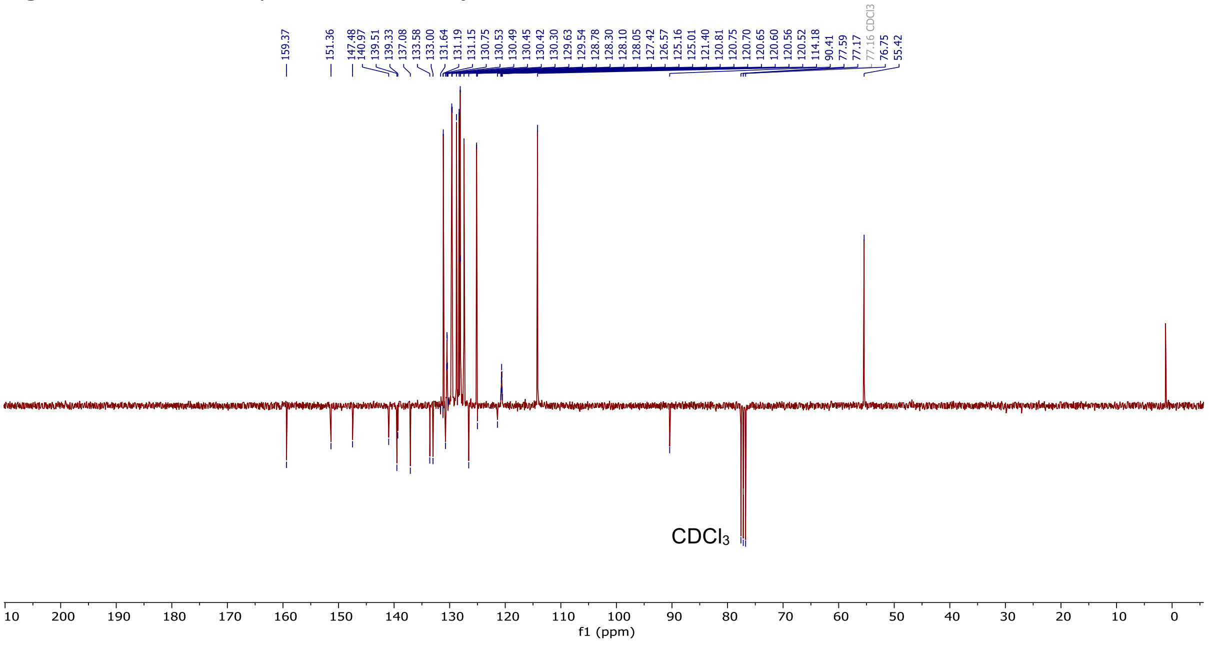

Figure S12. $^{13}C\{^{1}H\}$-Jmod NMR spectrum of **1,2-Cp^{Ar5}-OH** in $CDCl_3$ at 293 K, 75 MHz.

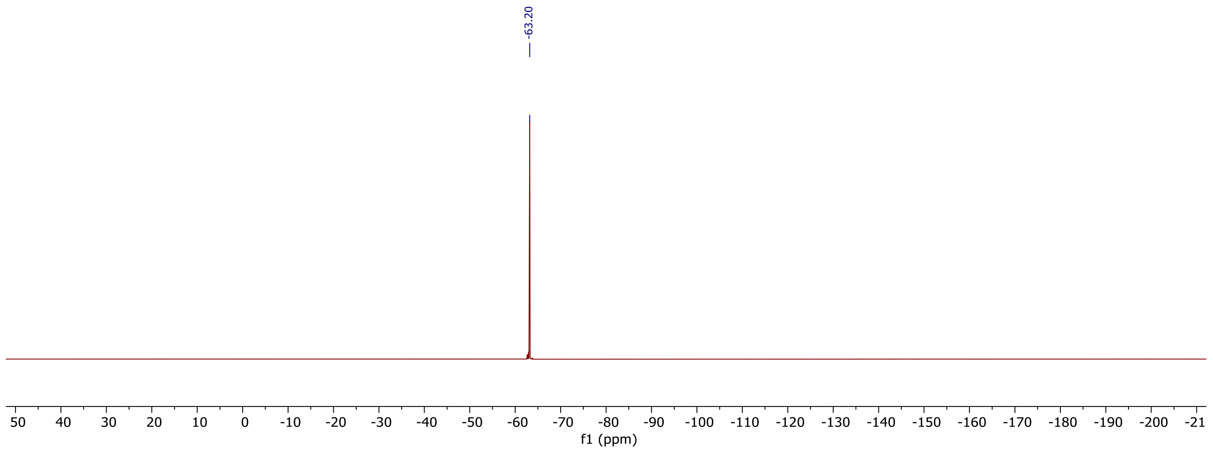

Figure S13. $^{19}F\{^{1}H\}$-NMR spectrum of **1,2-Cp^{Ar5}-OH** in $CDCl_3$ at 293 K, 282 MHz.

Compound 1,2-Cp^{Ar5}[Ru]Cl(CO)$_2$

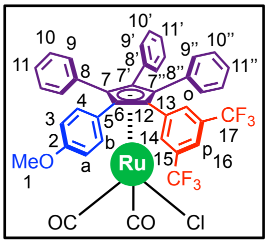

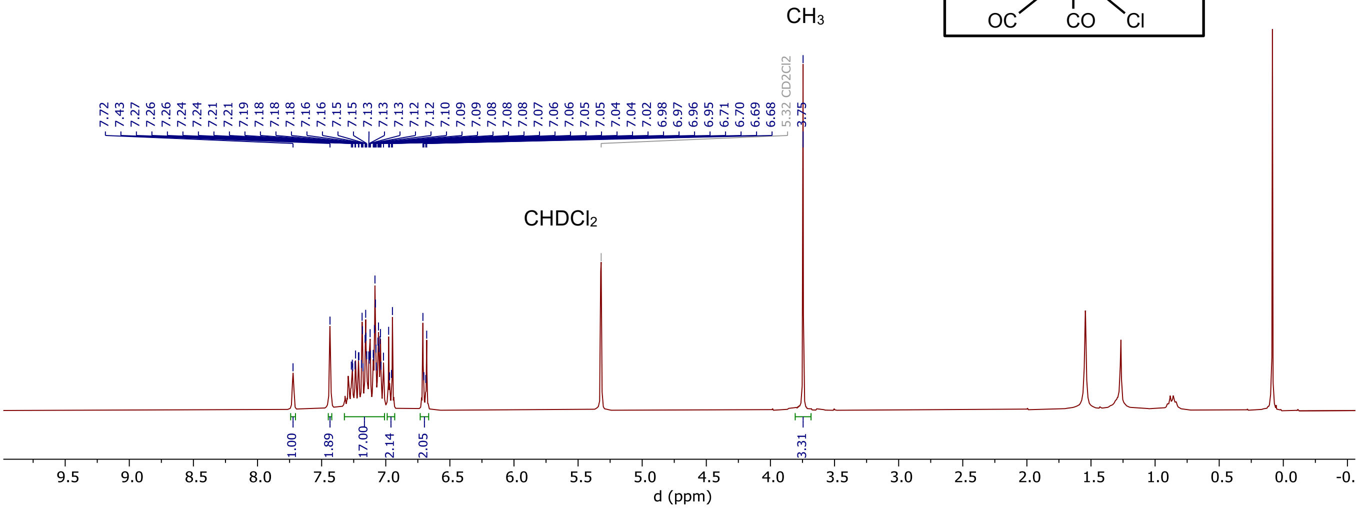

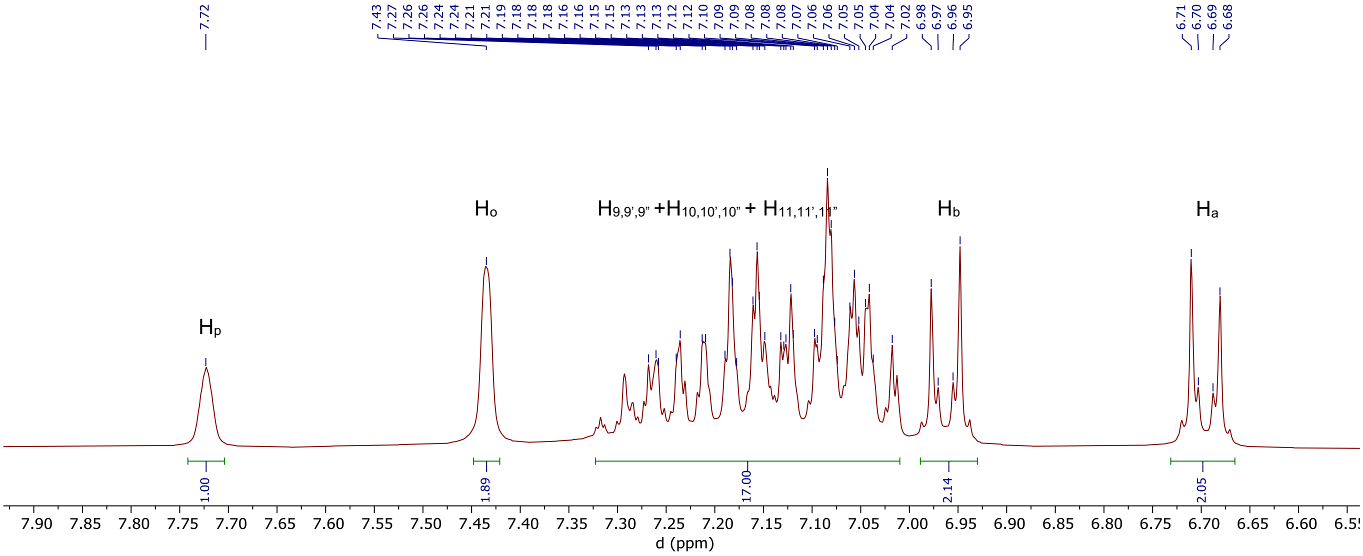

Figure S14. ^{1}H-NMR spectrum of **1,2-Cp^{Ar5}[Ru]Cl(CO)$_2$** in CD_2Cl_2 at 293 K, 300 MHz.

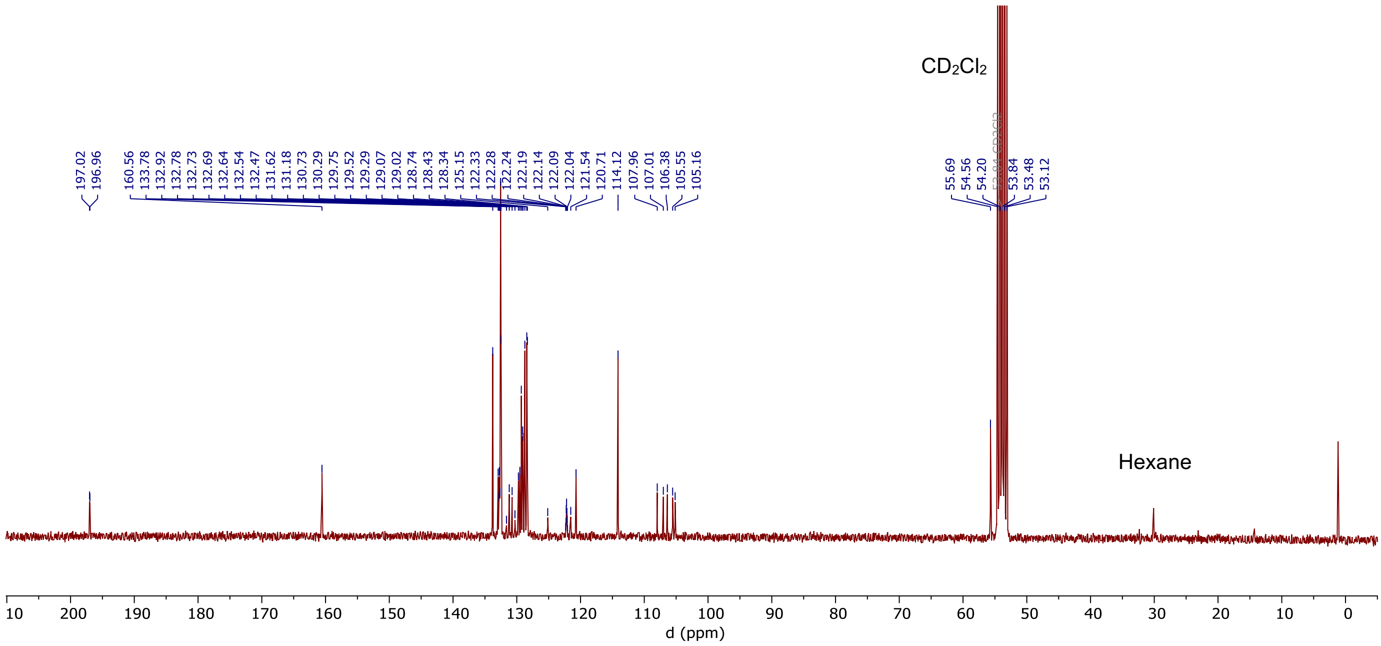

Figure S15. $^{13}C\{^1H\}$-NMR spectrum of **1,2-Cp^{Ar5}[Ru]Cl(CO)$_2$** in CD_2Cl_2 at 293 K, 75 MHz.

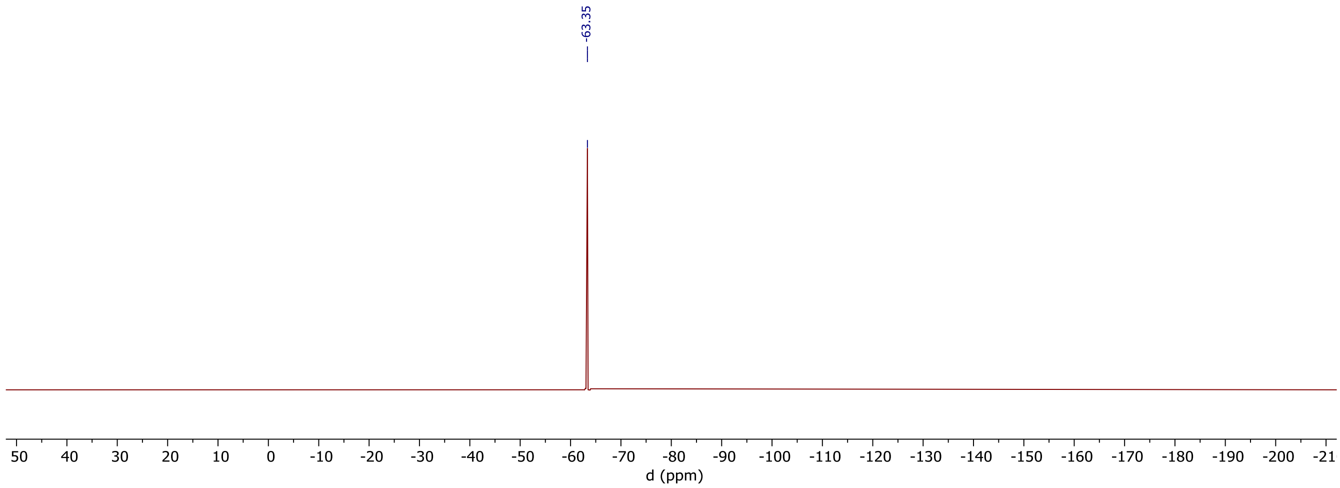

Figure S16. $^{19}F\{^1H\}$-NMR spectrum of **1,2-Cp^{Ar5}[Ru]Cl(CO)$_2$** in CD_2Cl_2 at 293 K, 282 MHz.

Compound 1,2-Cp^{Ar5}[Ru]Tp

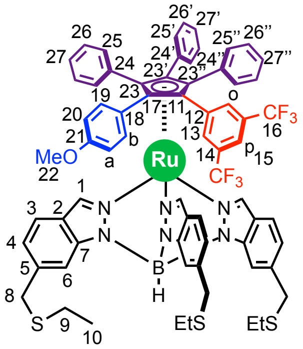

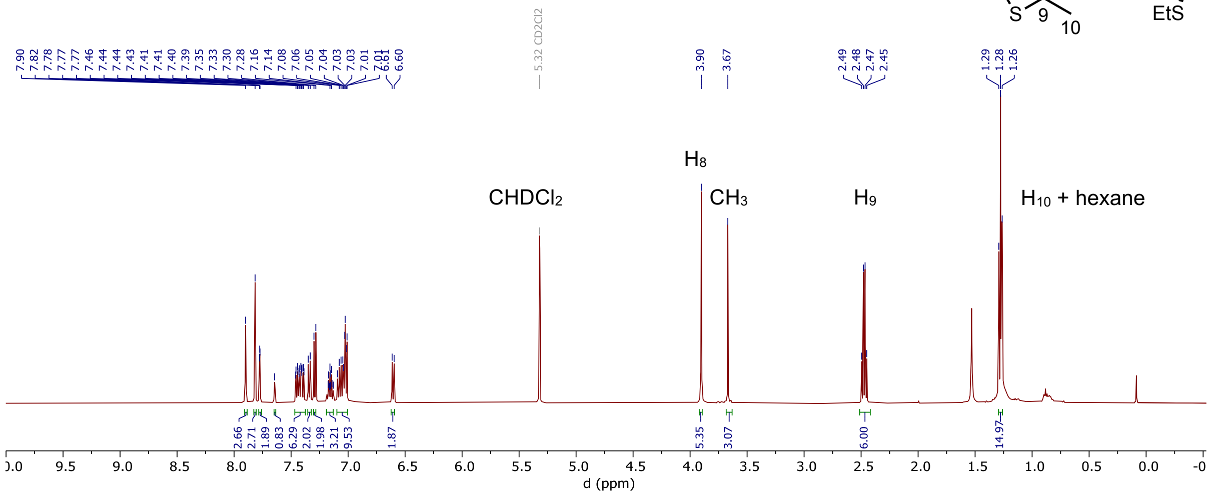

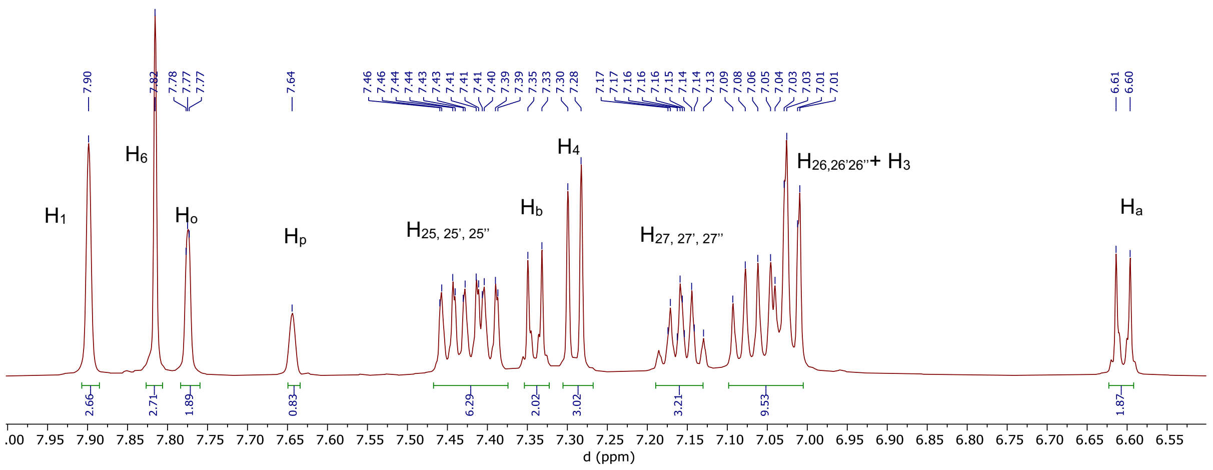

Figure S17. ^{1}H-NMR spectrum of **1,2-Cp^{Ar5}[Ru]Tp** in CD_2Cl_2 at 293 K, 500 MHz.

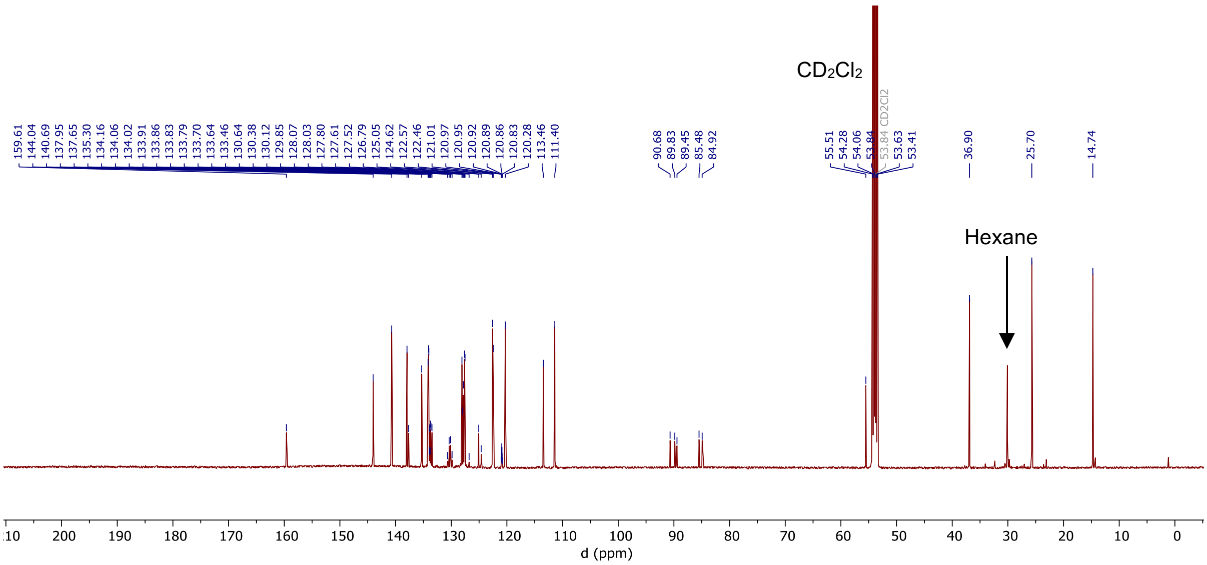

Figure S18. $^{13}C\{^{1}H\}$-NMR spectrum of **1,2-Cp^{Ar5}[Ru]Tp** in CD_2Cl_2 at 293 K, 125 MHz.

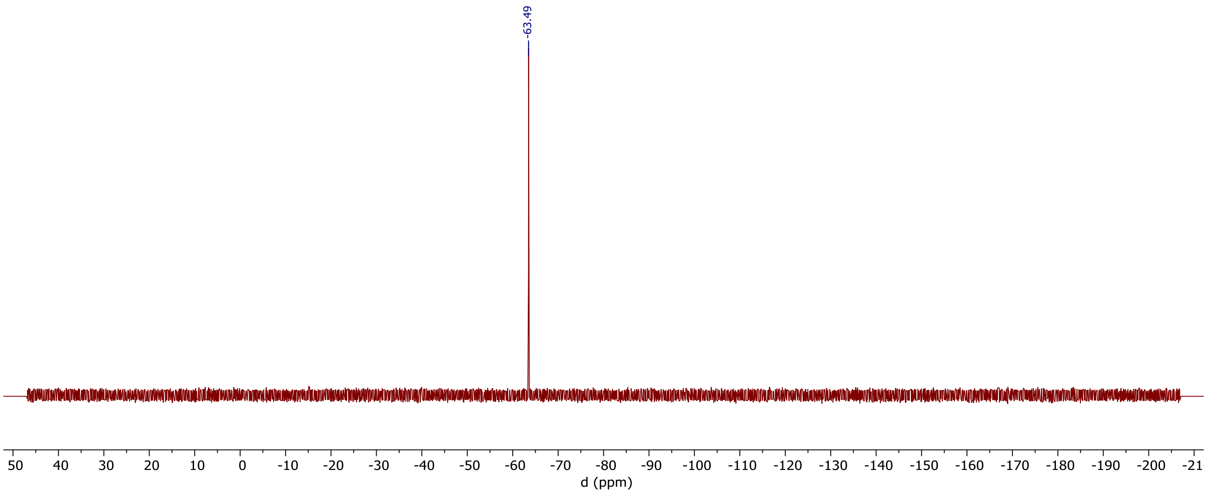

Figure S19. $^{19}F\{^{1}H\}$-NMR spectrum of **1,2-Cp^{Ar5}[Ru]Tp** in CD_2Cl_2 at 293 K, 282 MHz.

Compound 5

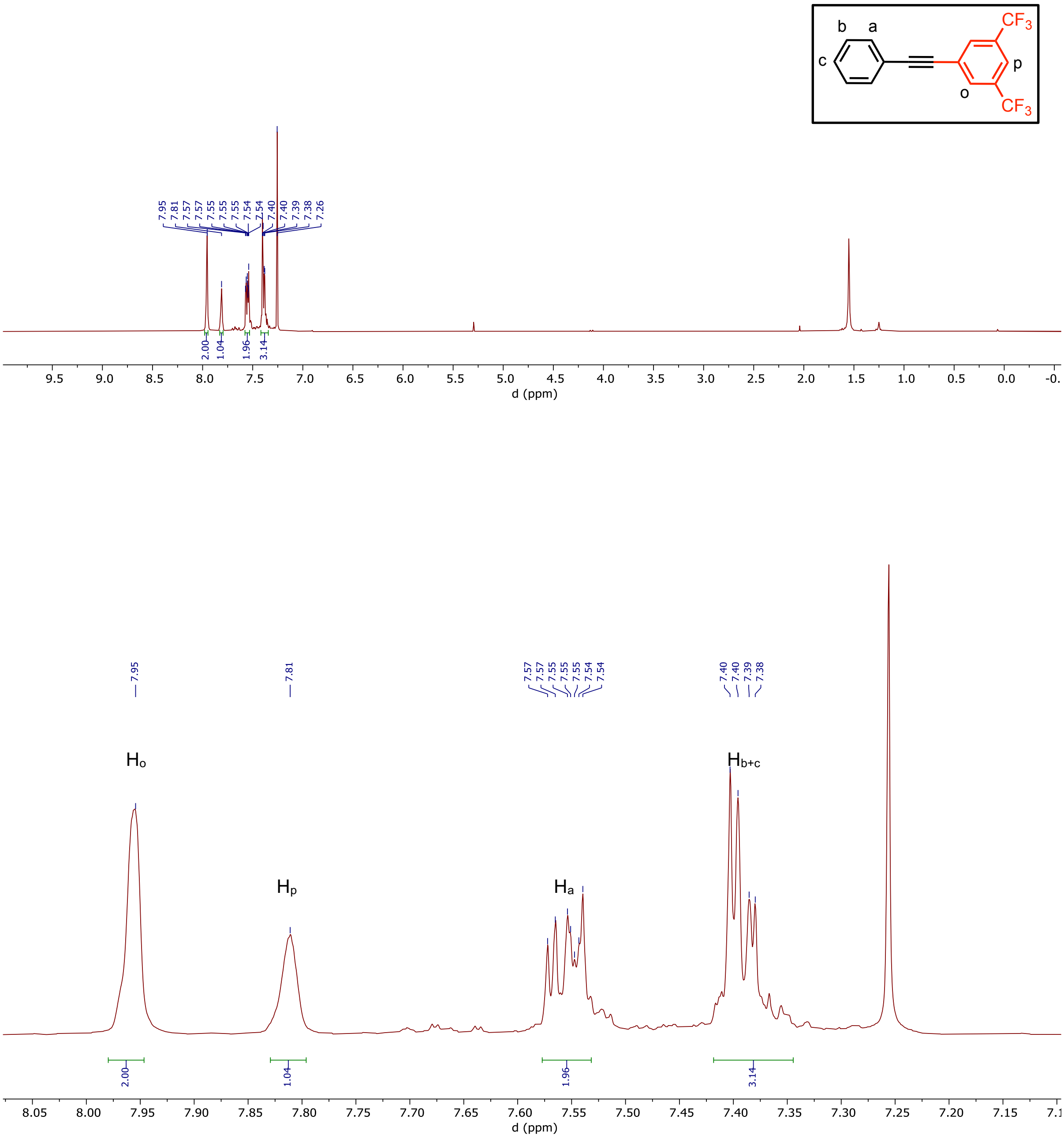

Figure S20. ^{1}H-NMR spectrum of **5** in $CDCl_3$ at 293 K, 300 MHz.

Compound 6

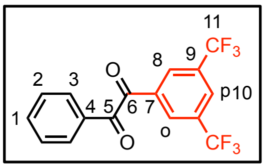

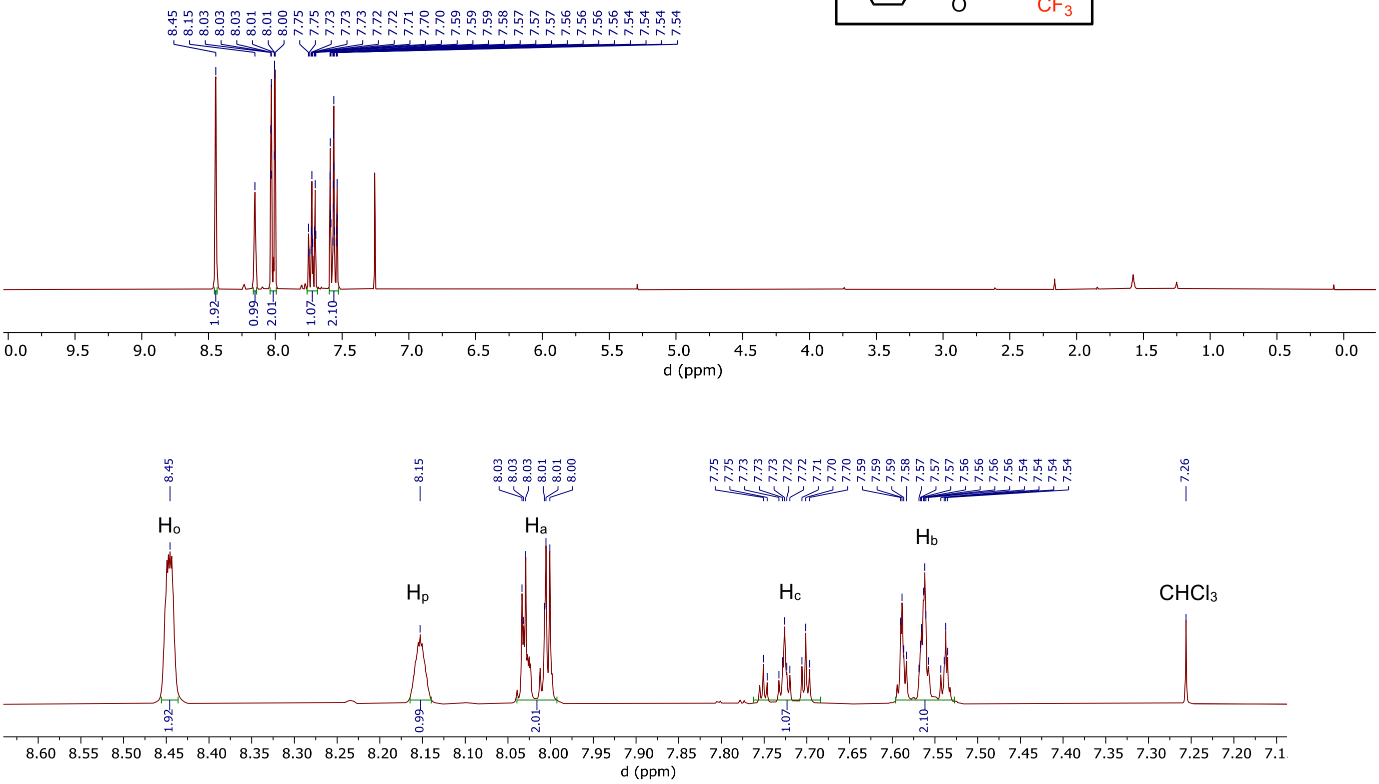

Figure S21. ^{1}H-NMR spectrum of **6** in $CDCl_3$ at 293 K, 300 MHz.

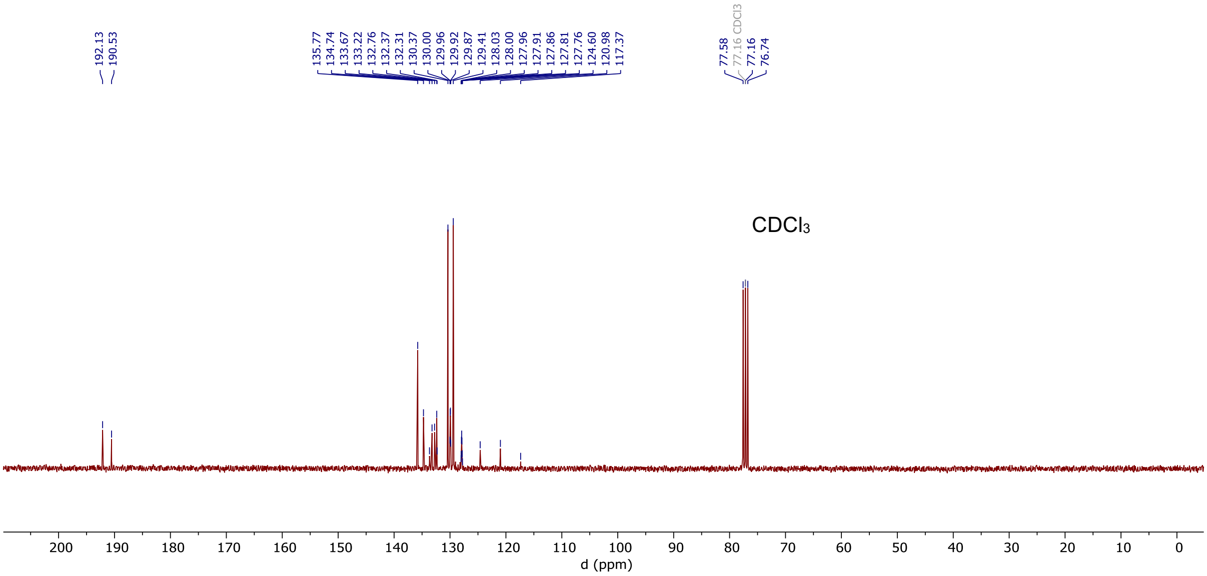

Figure S22. $^{13}C\{^{1}H\}$-NMR spectrum of **6** in $CDCl_3$ at 293 K, 75 MHz.

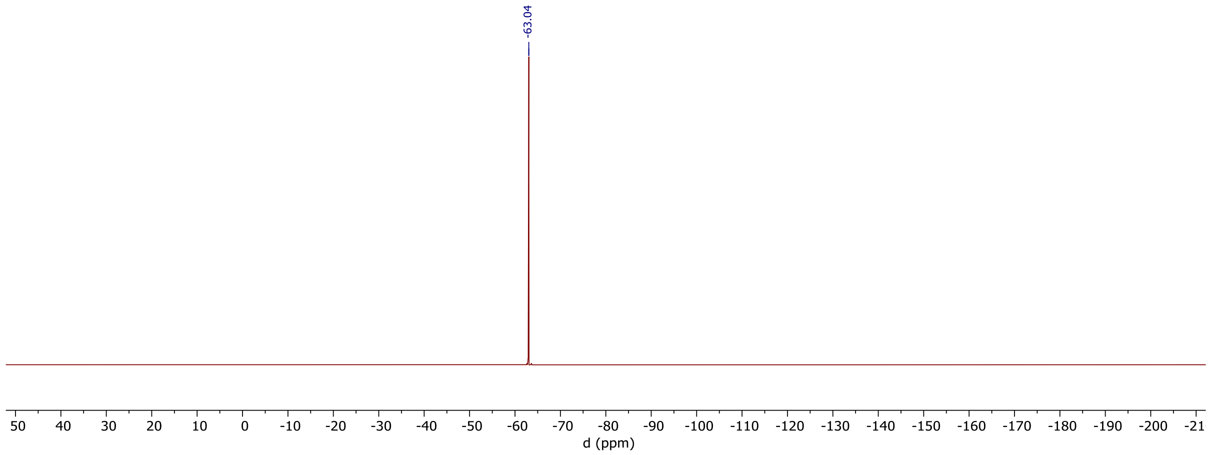

Figure S23. $^{19}F\{^{1}H\}$-NMR spectrum of **6** in $CDCl_3$ at 293 K, 282 MHz.

Compound 7

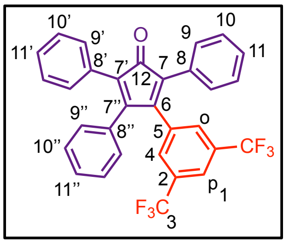

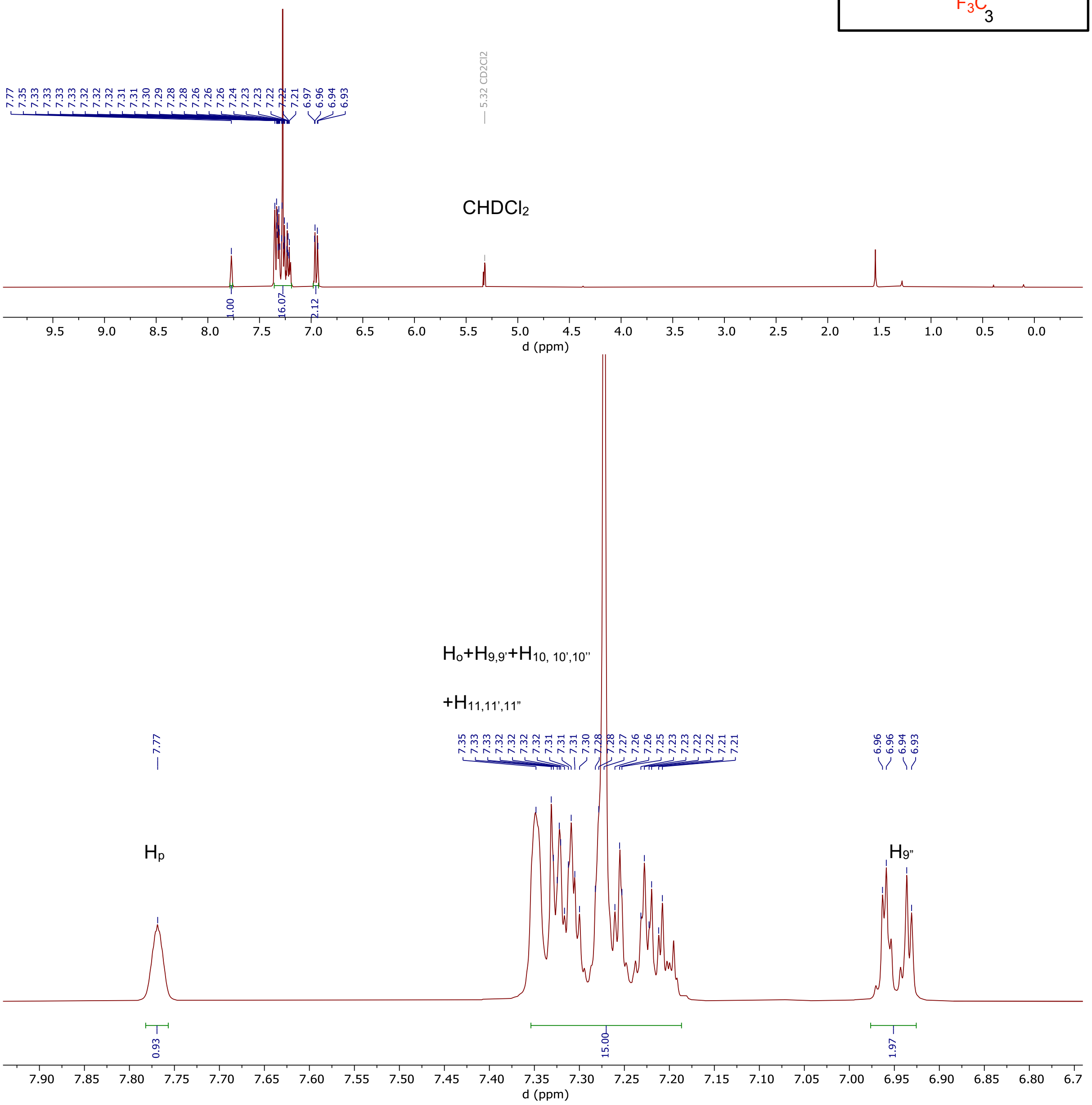

Figure S24. ^{1}H-NMR spectrum of **7** in CD_2Cl_2 at 293 K, 300 MHz.

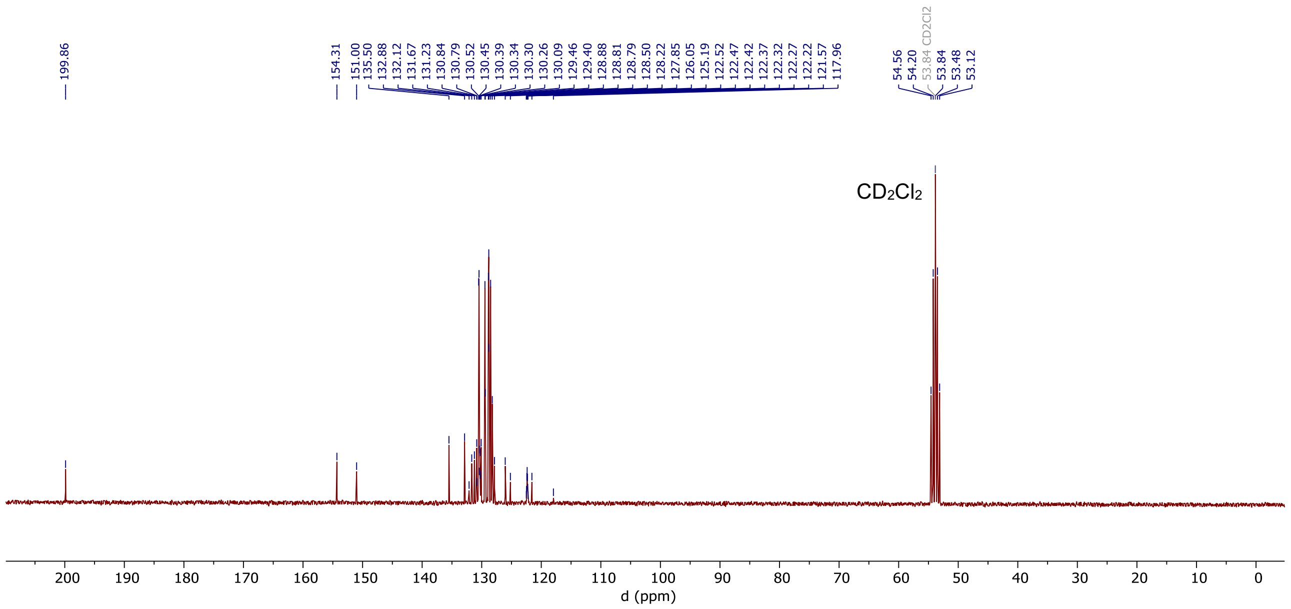

Figure S25. $^{13}C\{^{1}H\}$-NMR spectrum of **7** in CD_2Cl_2 at 293 K, 75 MHz.

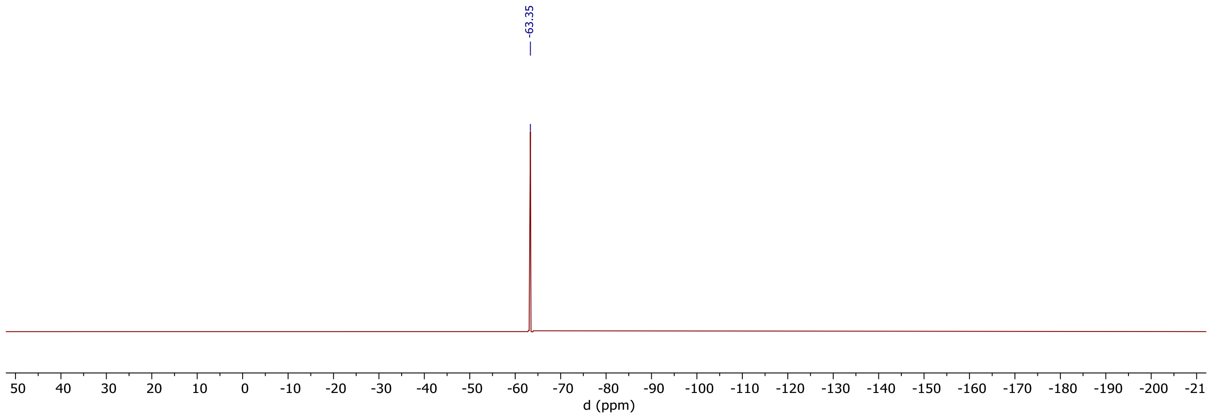

Figure S26. $^{19}F\{^{1}H\}$-NMR spectrum of **7** in CD_2Cl_2 at 293 K, 282 MHz.

Compound 1,3-Cp^{Ar5}-OH

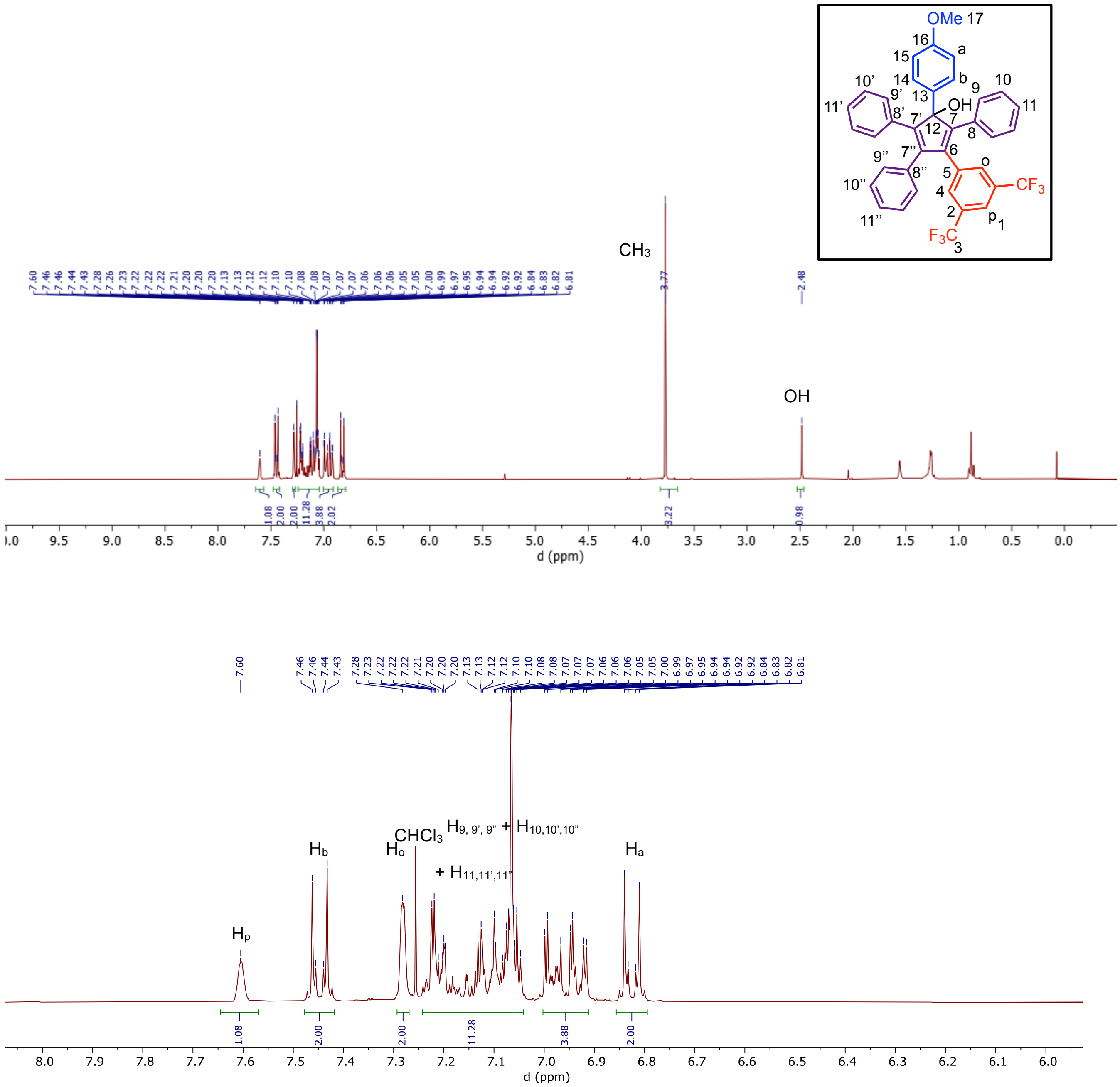

Figure S27. ^{1}H-NMR spectrum of **1,3-Cp^{Ar5}-OH** in $CDCl_3$ at 293 K, 300 MHz.

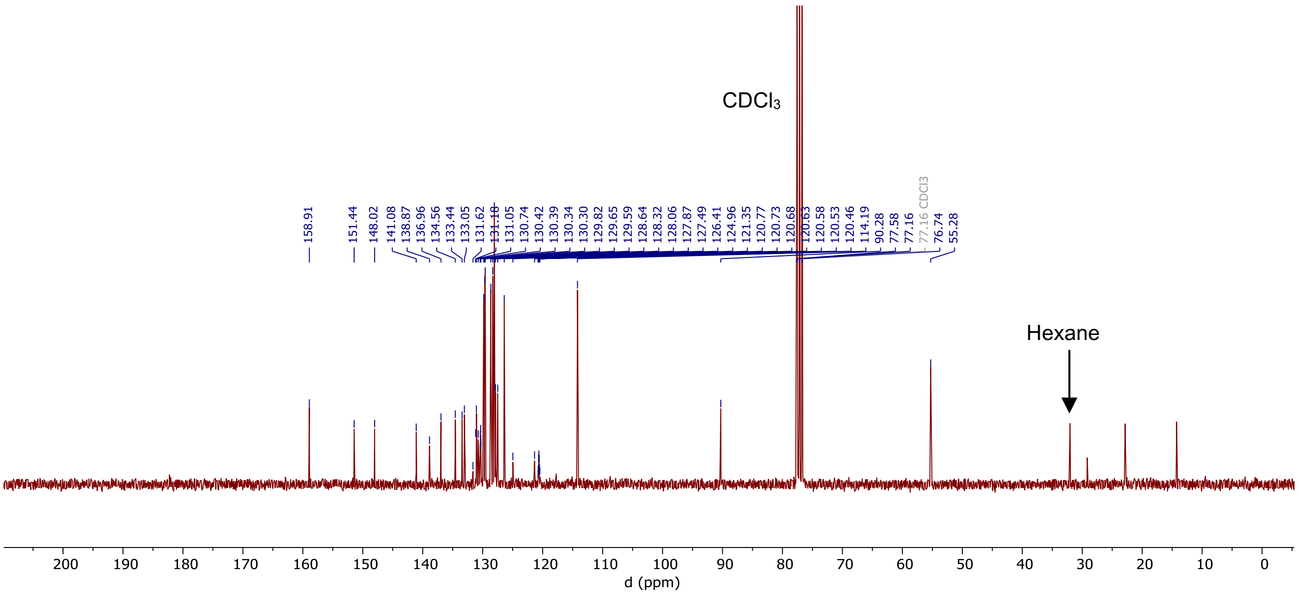

Figure S28. $^{13}C\{^{1}H\}$-NMR spectrum of **1,3-Cp^{Ar5}-OH** in $CDCl_3$ at 293 K, 75 MHz.

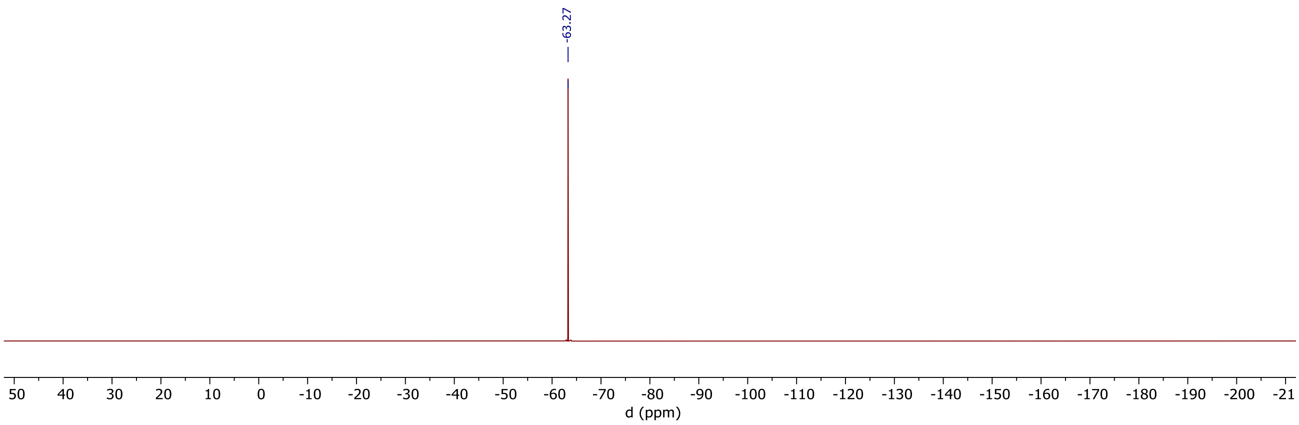

Figure S29. $^{19}F\{^{1}H\}$-NMR spectrum of **1,3-Cp^{Ar5}-OH** in $CDCl_3$ at 293 K, 282 MHz.

Compound 1,3-Cp^{Ar5}[Ru]Cl(CO)$_2$

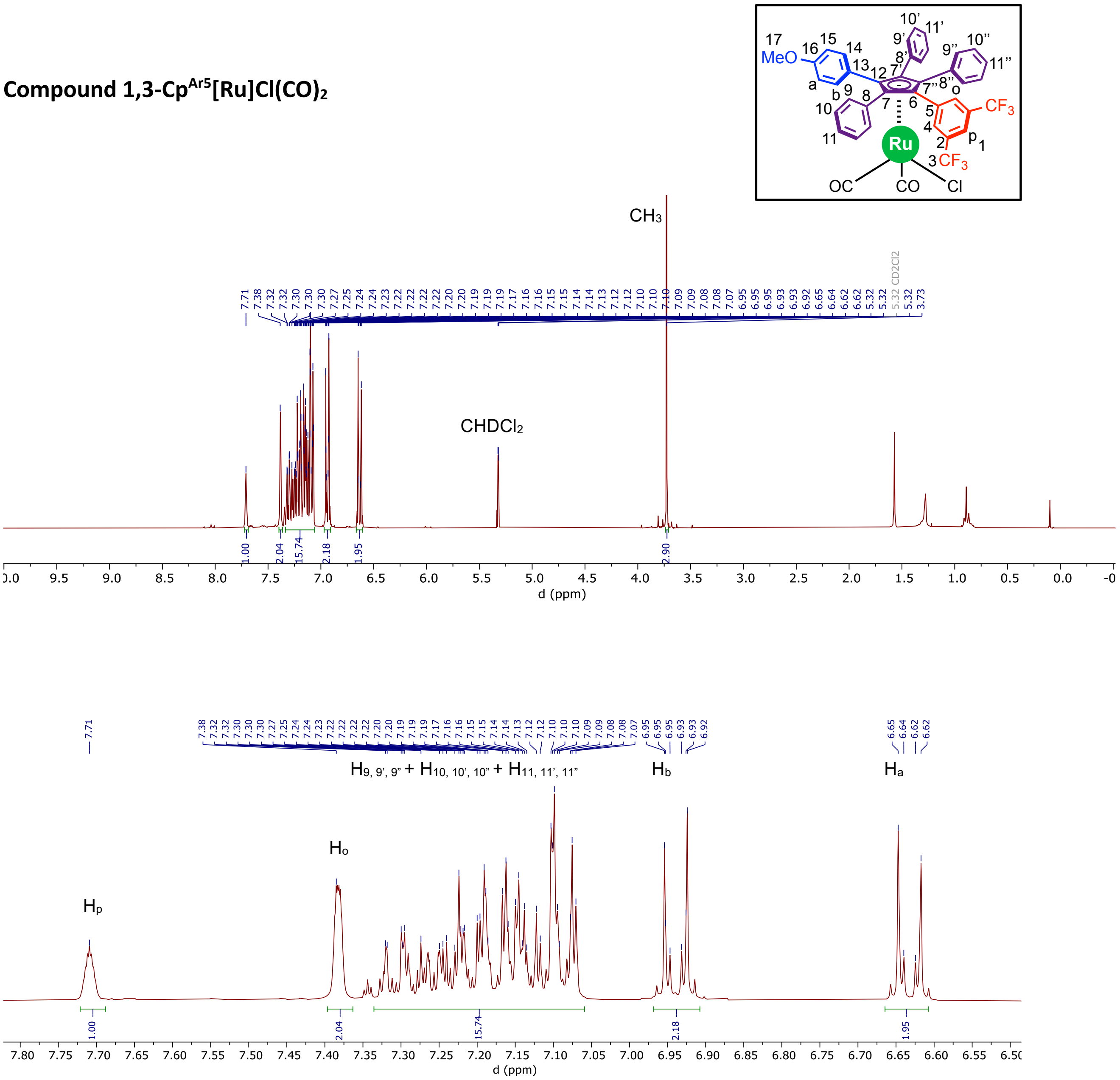

Figure S30. ^{1}H-NMR spectrum of **1,3-Cp^{Ar5}[Ru]Cl(CO)$_2$** in CD$_2$Cl$_2$ at 293 K, 300 MHz.

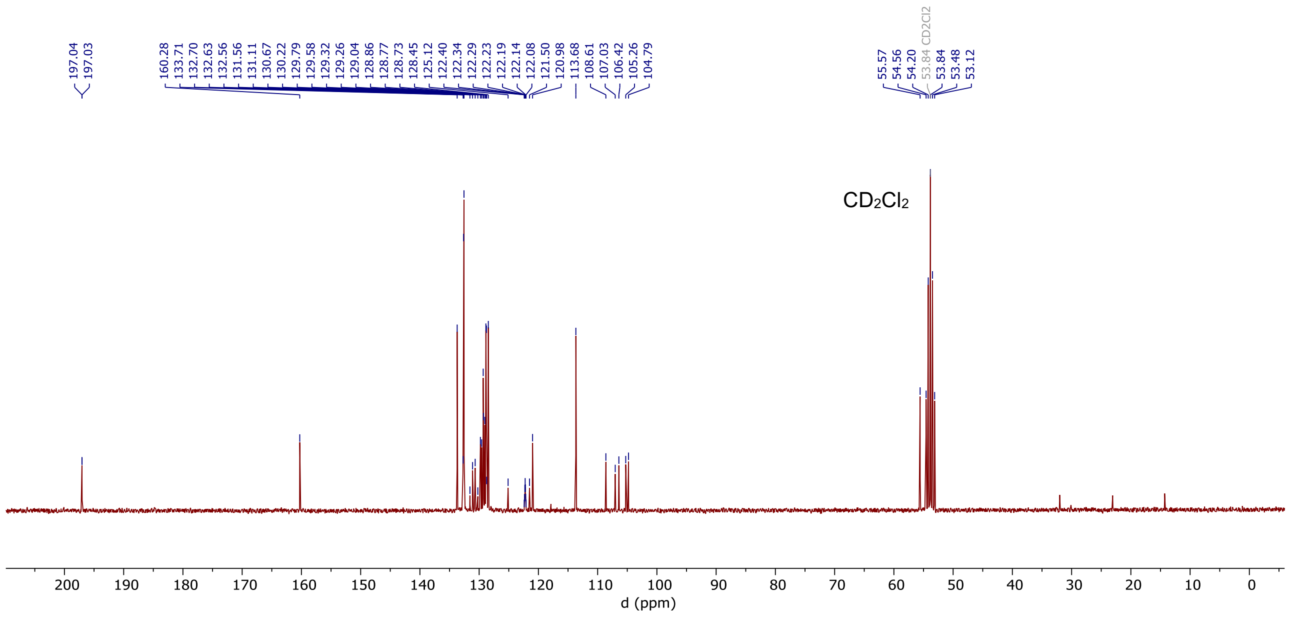

Figure S31. $^{13}C\{^{1}H\}$-NMR spectrum of **1,3-Cp^{Ar5}[Ru]Cl(CO)$_2$** in CD_2Cl_2 at 293 K, 75 MHz.

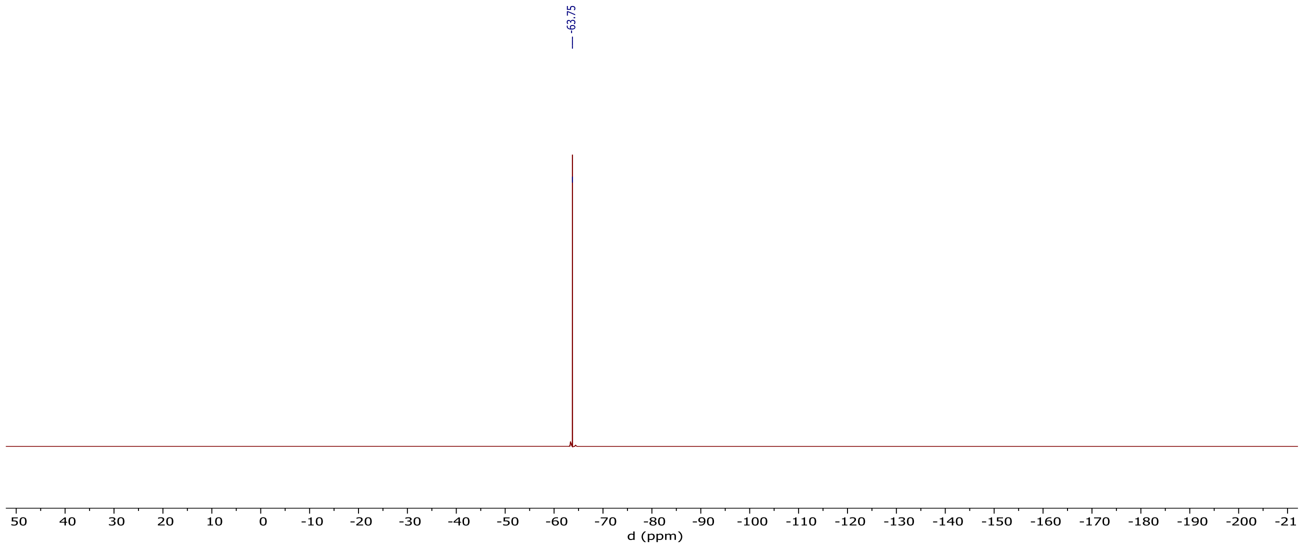

Figure S32. $^{19}F\{^{1}H\}$-NMR spectrum of **1,3-Cp^{Ar5}[Ru]Cl(CO)$_2$** in CD_2Cl_2 at 293 K, 282 MHz.

Compound 1,3-Cp^{Ar5}[Ru]Tp

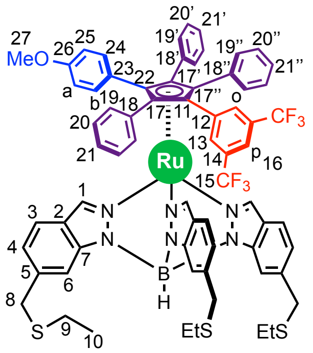

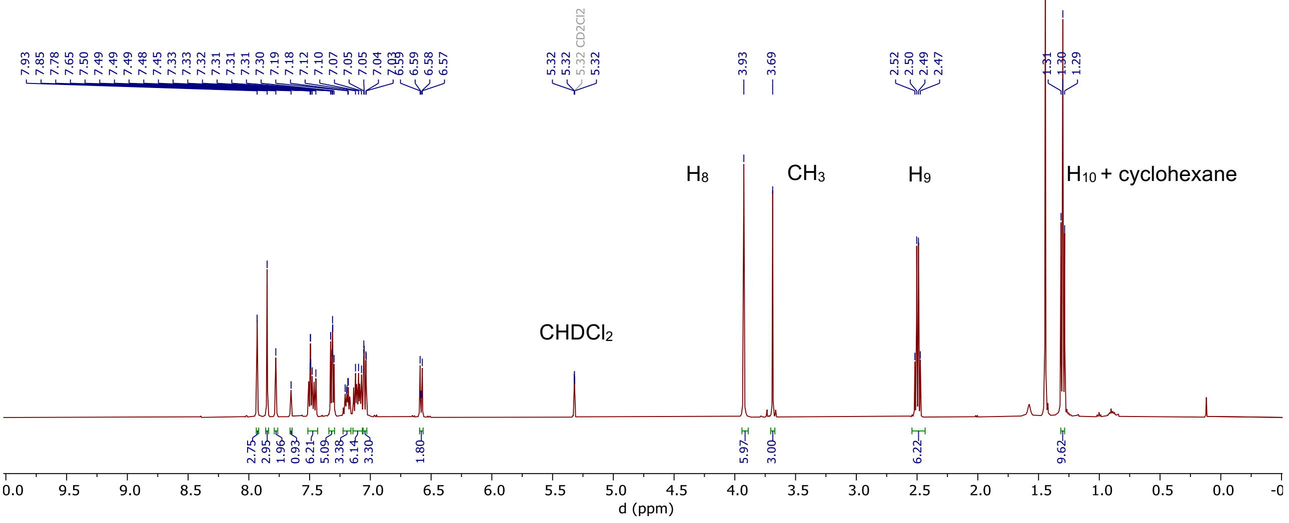

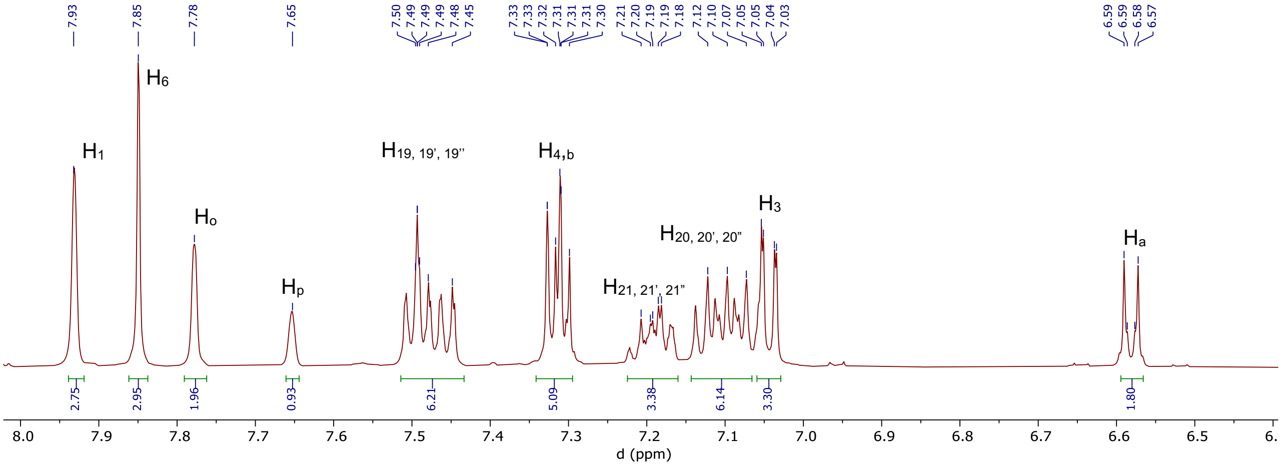

Figure S33. ^{1}H-NMR spectrum of **1,3-Cp^{Ar5}[Ru]Tp** in CD_2Cl_2 at 293 K, 500 MHz.

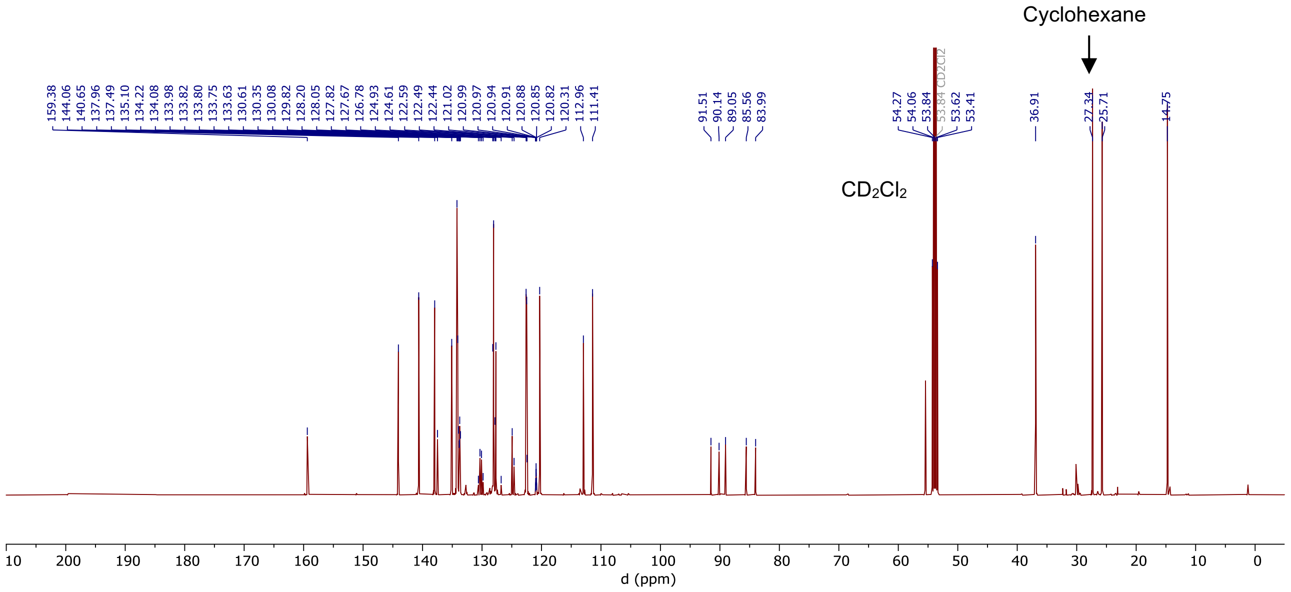

Figure S34. $^{13}C\{^{1}H\}$-NMR spectrum of **1,3-Cp^{Ar5}[Ru]Tp** in CD_2Cl_2 at 293 K, 125 MHz.

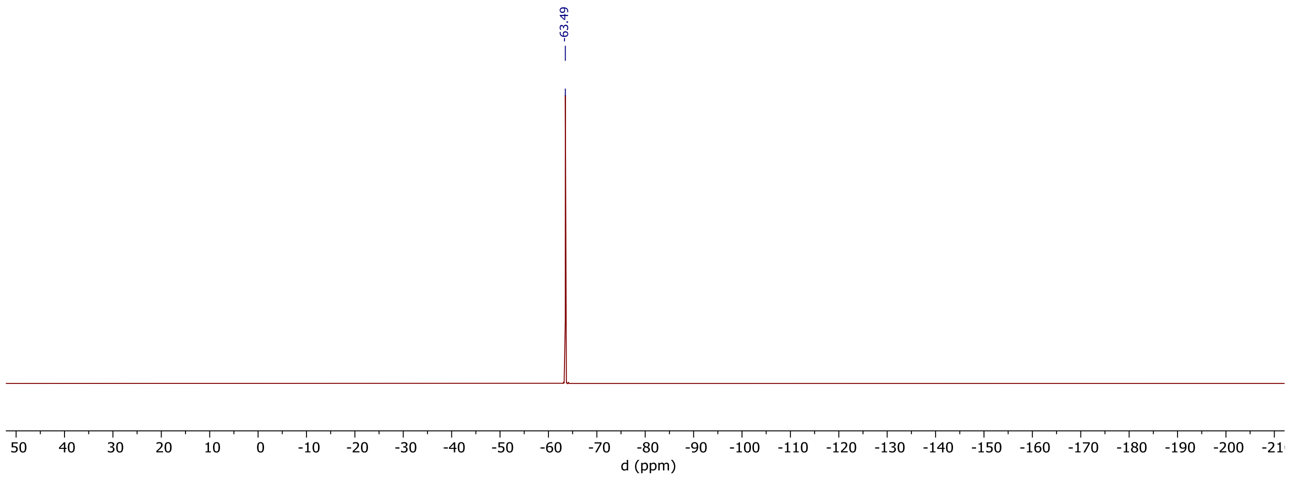

Figure S35. $^{19}F\{^{1}H\}$-NMR spectrum of **1,3-Cp^{Ar5}[Ru]Tp** in CD_2Cl_2 at 293 K, 282 MHz.

II. HR-MS data

Compound 3

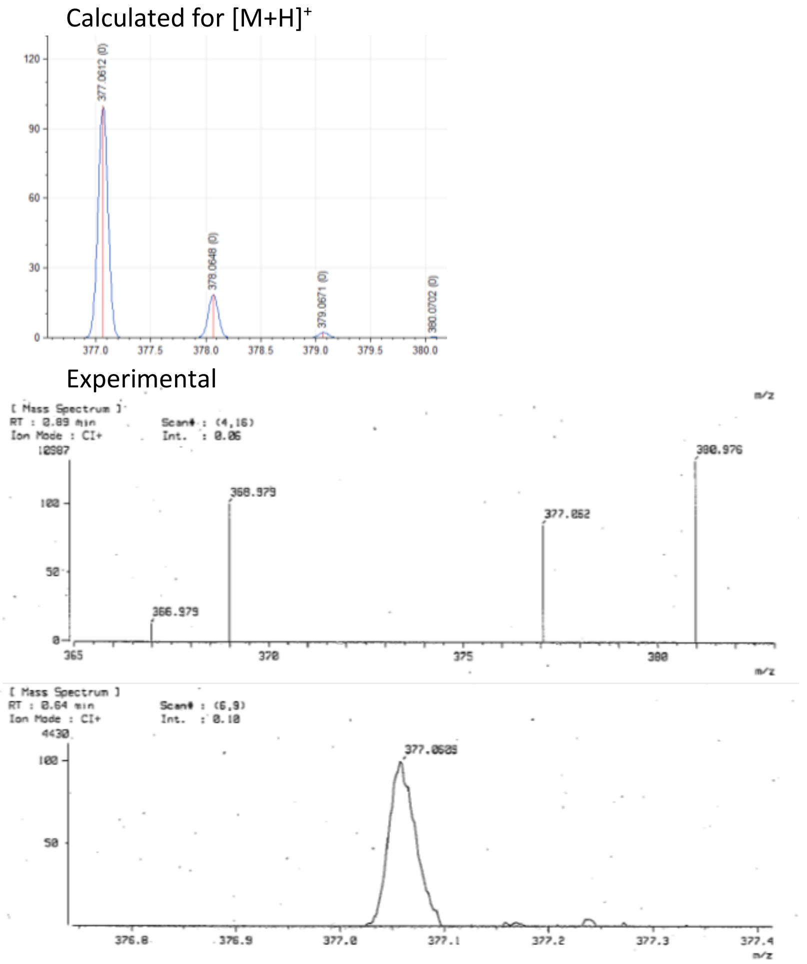

Figure S36. DCI-CH_4 MS spectrum of **3**.

Compound 3'

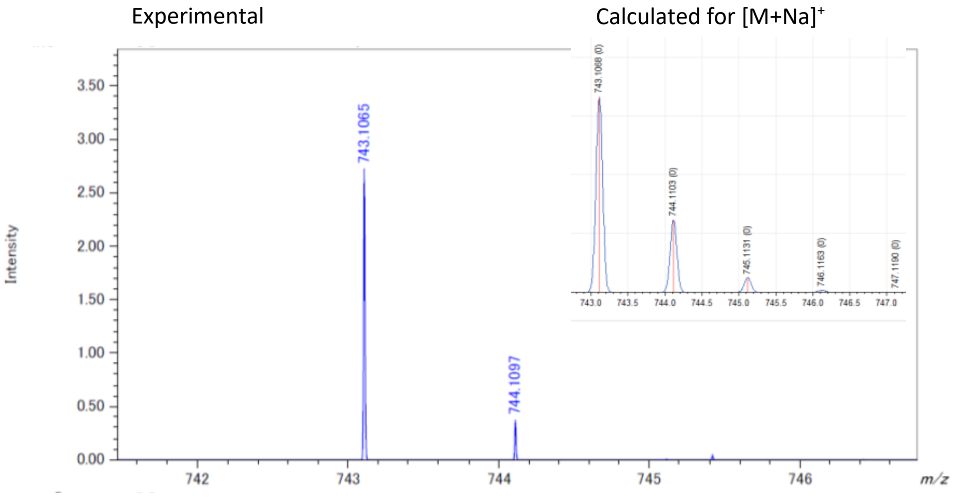

Figure S37. MALDI-TOF MS spectrum of **3'**.

Compound 4

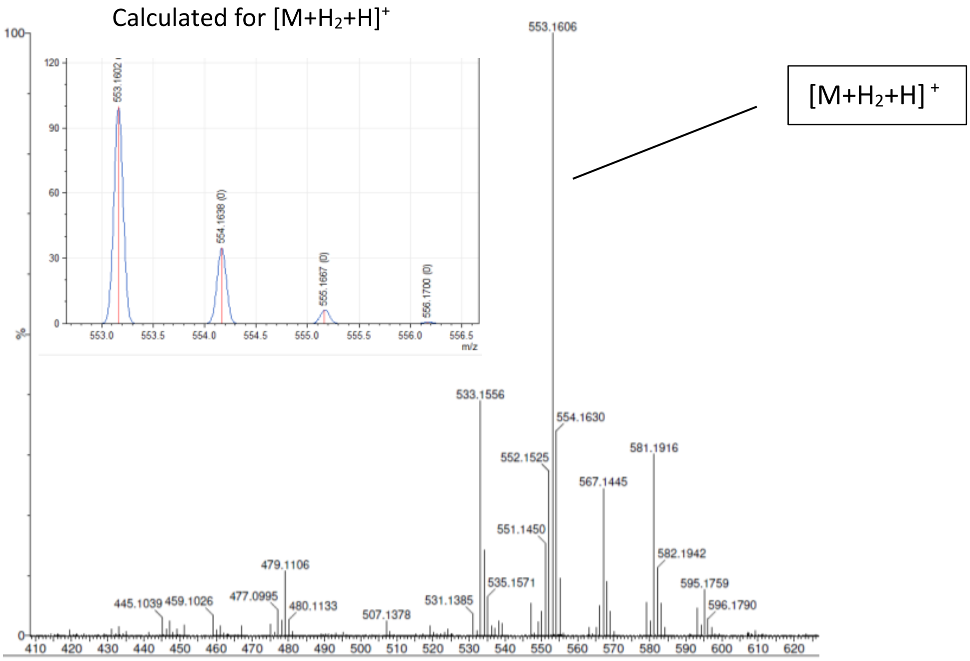

Figure S38. DCI-CH_4 MS spectrum of **4**.

Compound 1,2-Cp^{Ar5}-OH

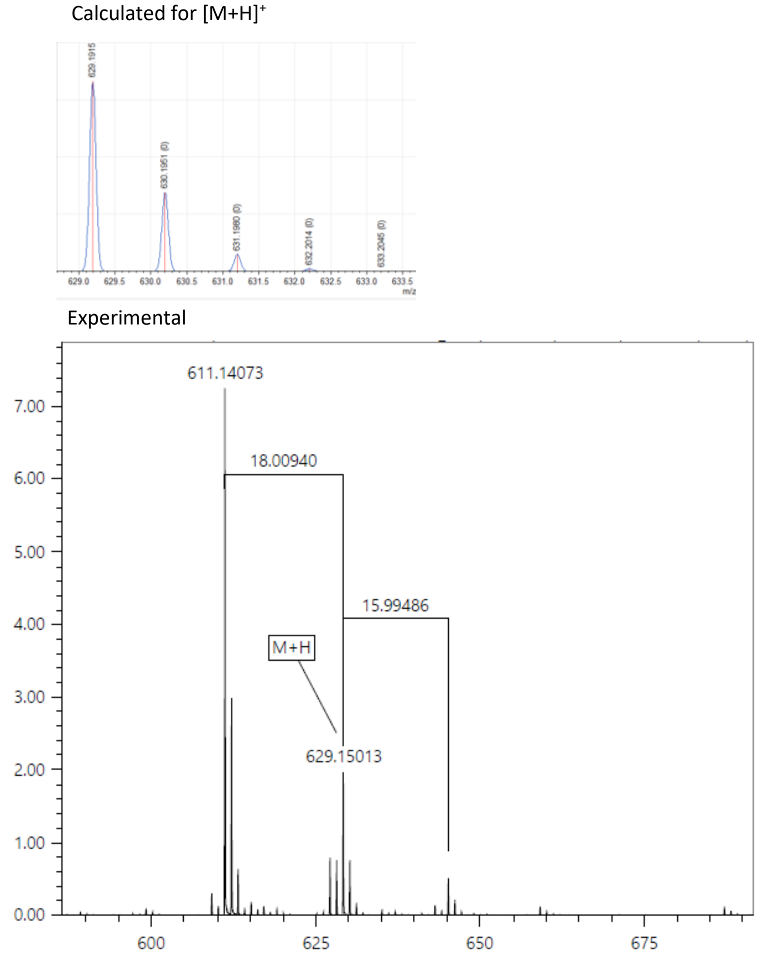

Figure S39. EI MS spectrum of **1,2-Cp^{Ar5}-OH**.

Compound 1,2-Cp^{Ar5}[Ru]Cl(CO)$_2$

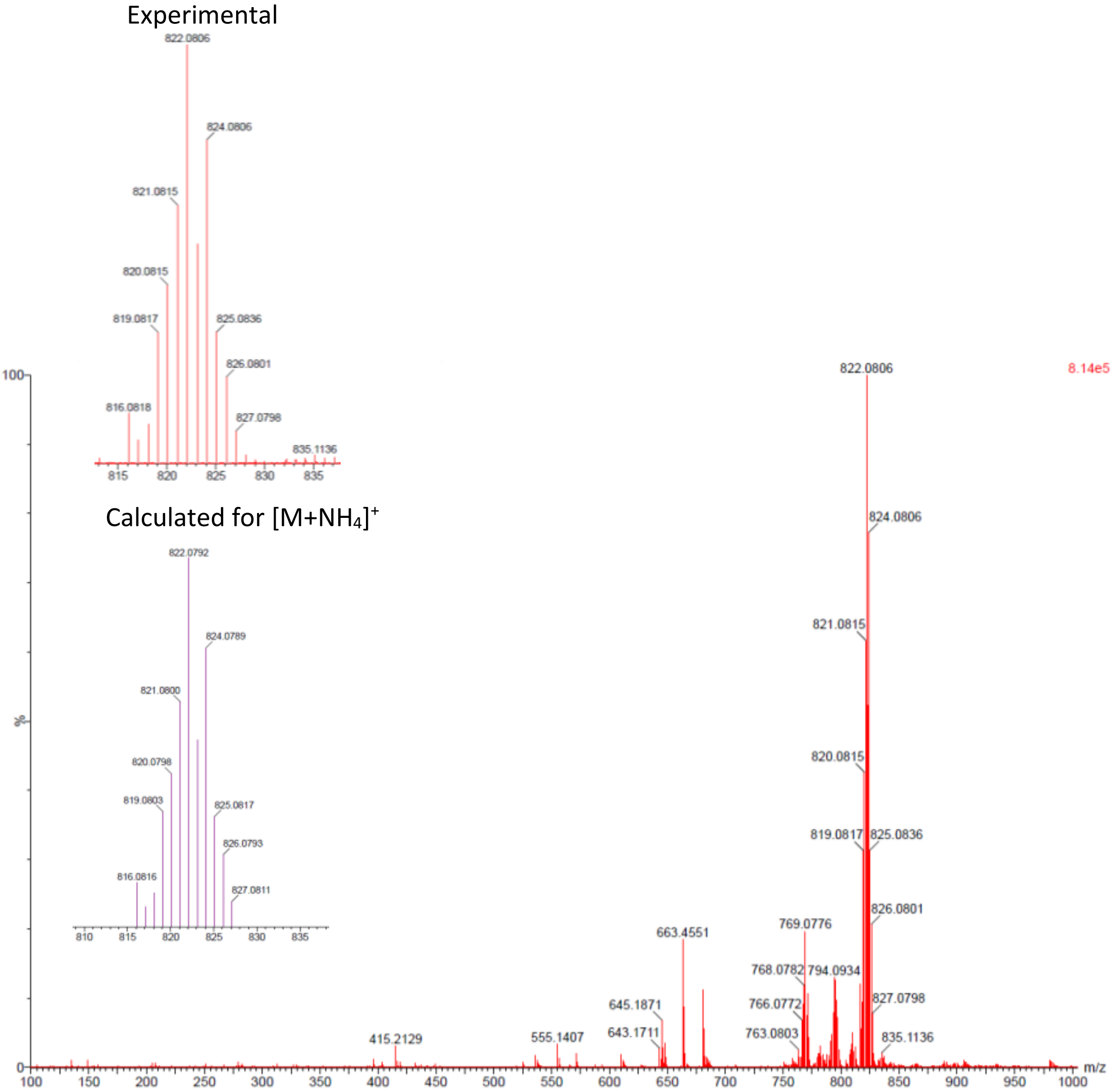

Figure S40. ESI MS spectrum of **1,2-Cp^{Ar5}[Ru]Cl(CO)$_2$**.

Compound 1,2-Cp^{Ar5}[Ru]Tp

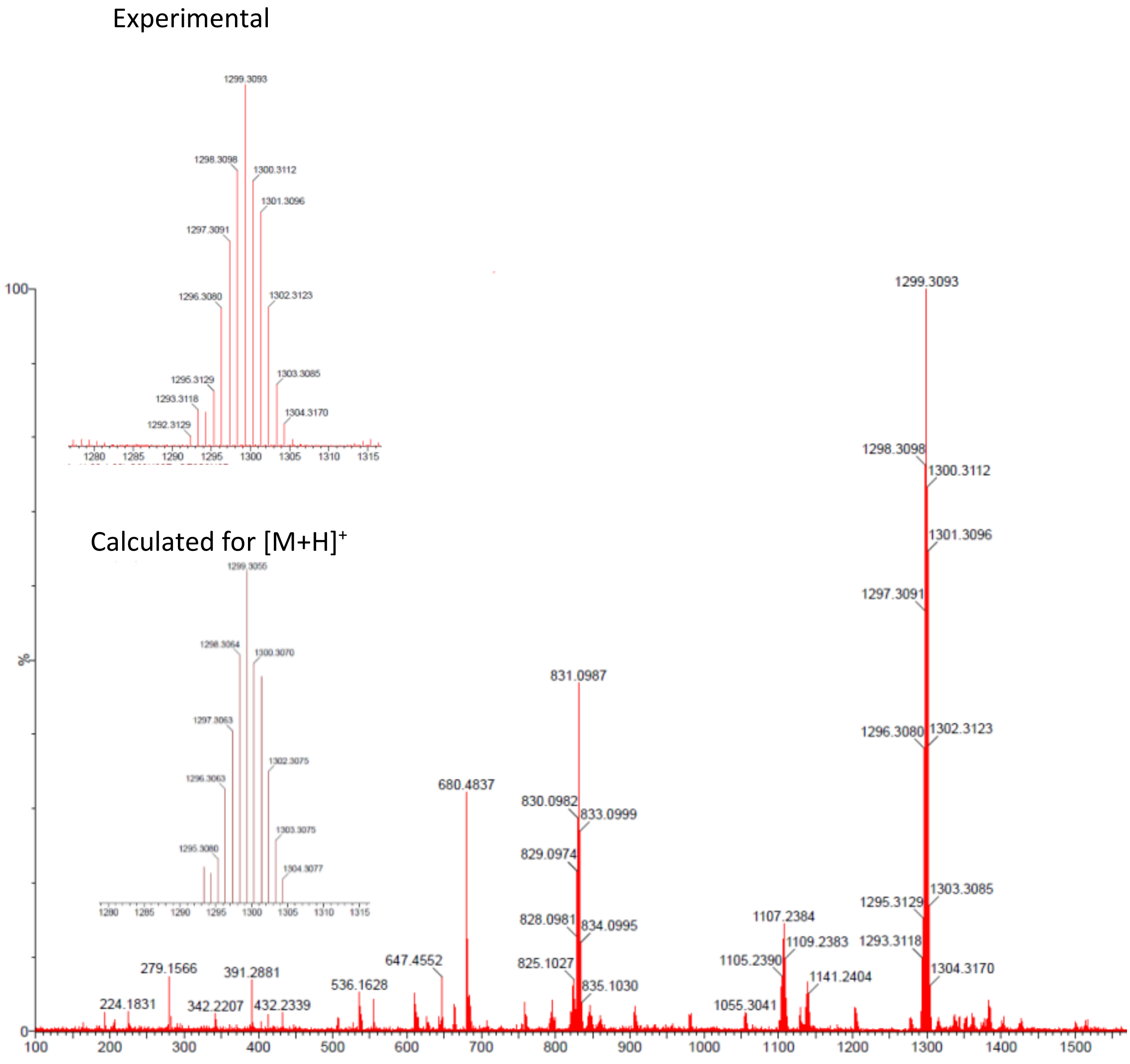

Figure S41. ESI MS spectrum of **1,2-Cp^{Ar5}[Ru]Tp**.

Compound 6

Calculated for [M-H]$^-$

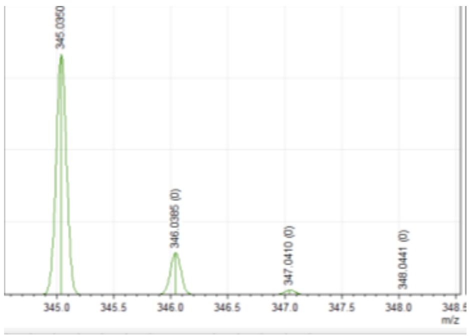

Experimental

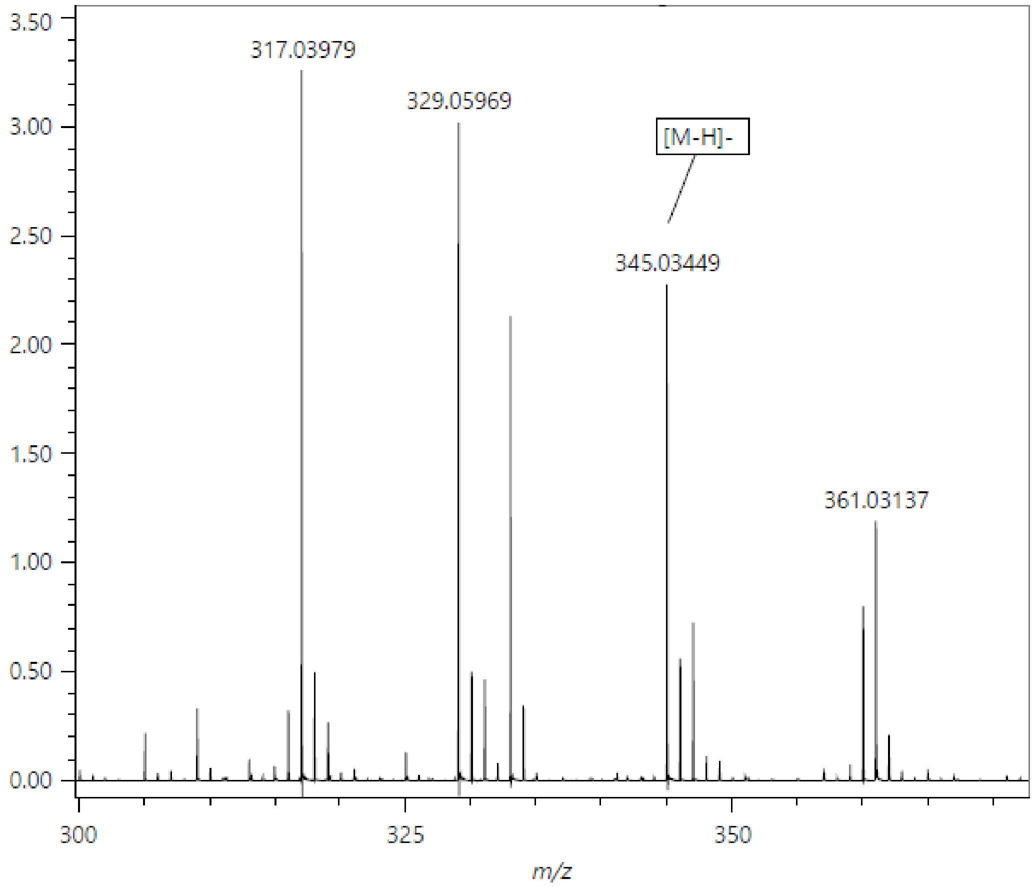

Figure S42. MALDI-TOF MS spectrum of **6**.

Compound 7

Calculated for [M+H]$^+$

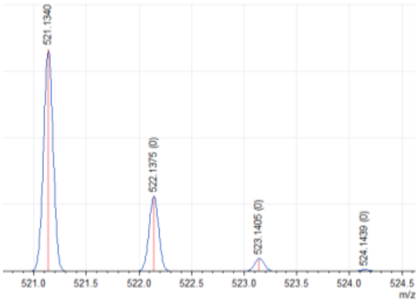

Experimental

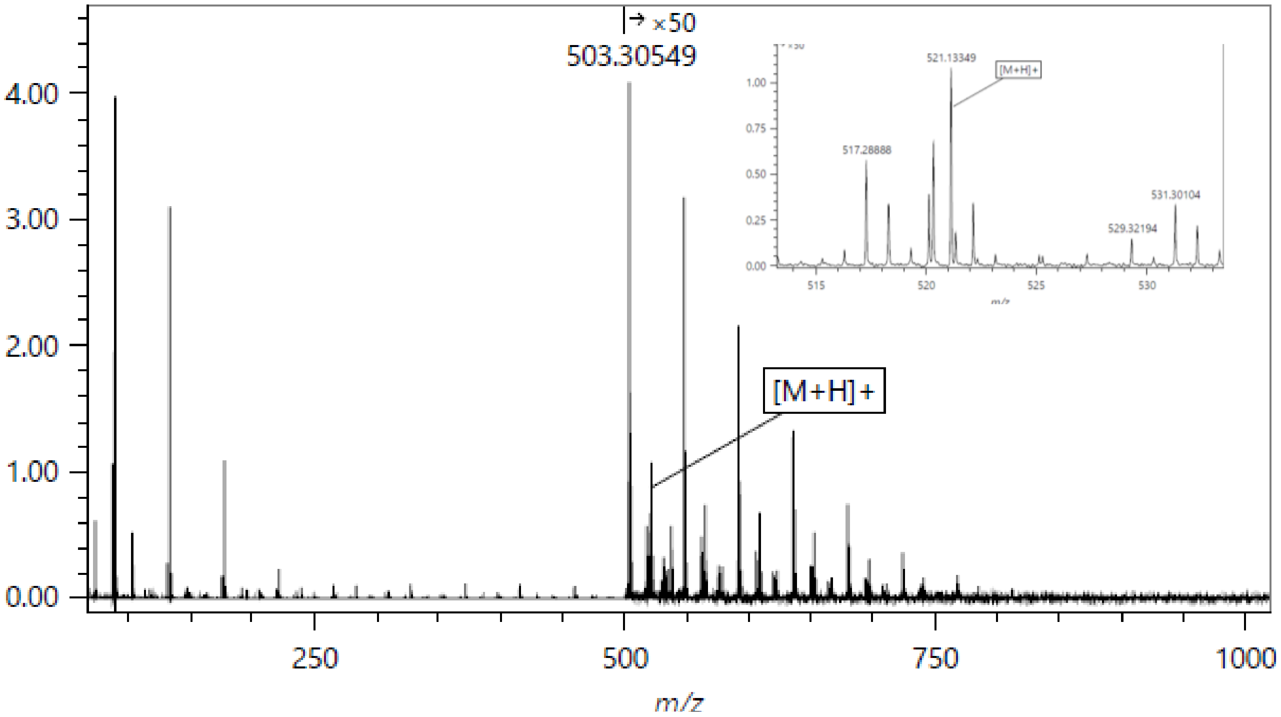

Figure S43. MALDI-TOF MS spectrum of **7**.

Compound 1,3-Cp^{Ar5}-OH

Calculated for $[M]^+$

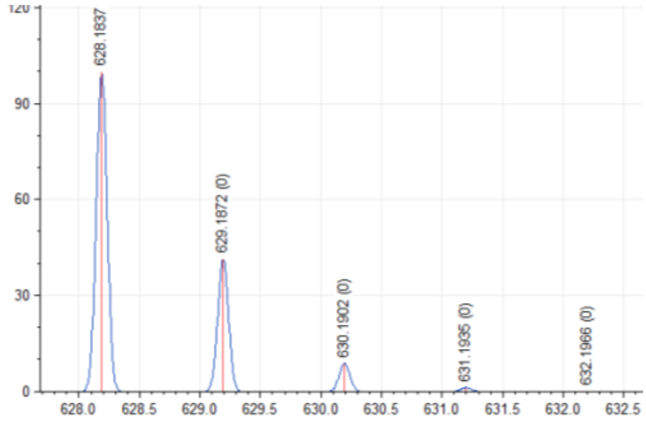

Experimental

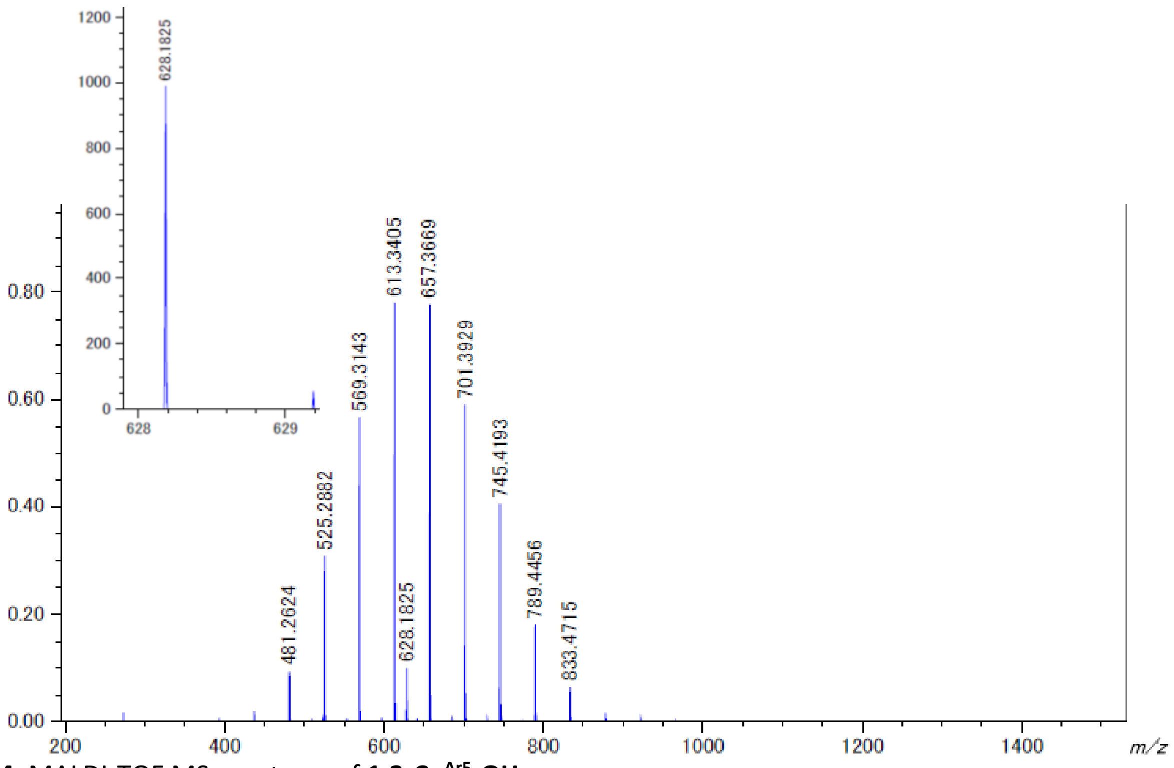

Figure S44. MALDI-TOF MS spectrum of **1,3-Cp^{Ar5}-OH**.

Compound 1,3-Cp^{Ar5}[Ru]Cl(CO)$_2$

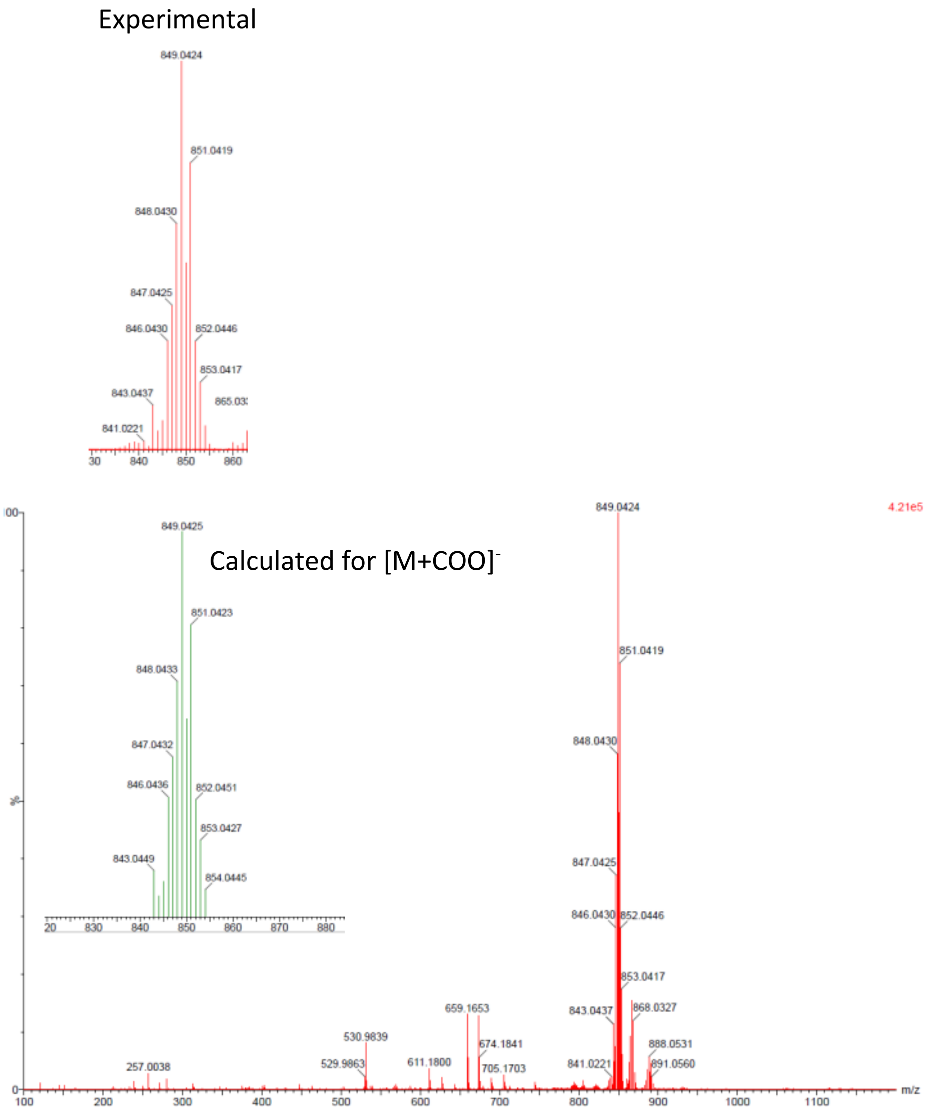

Figure S45. ESI MS spectrum of **1,3-Cp^{Ar5}[Ru]Cl(CO)$_2$**.

Compound 1,3-Cp^{Ar5}[Ru]Tp

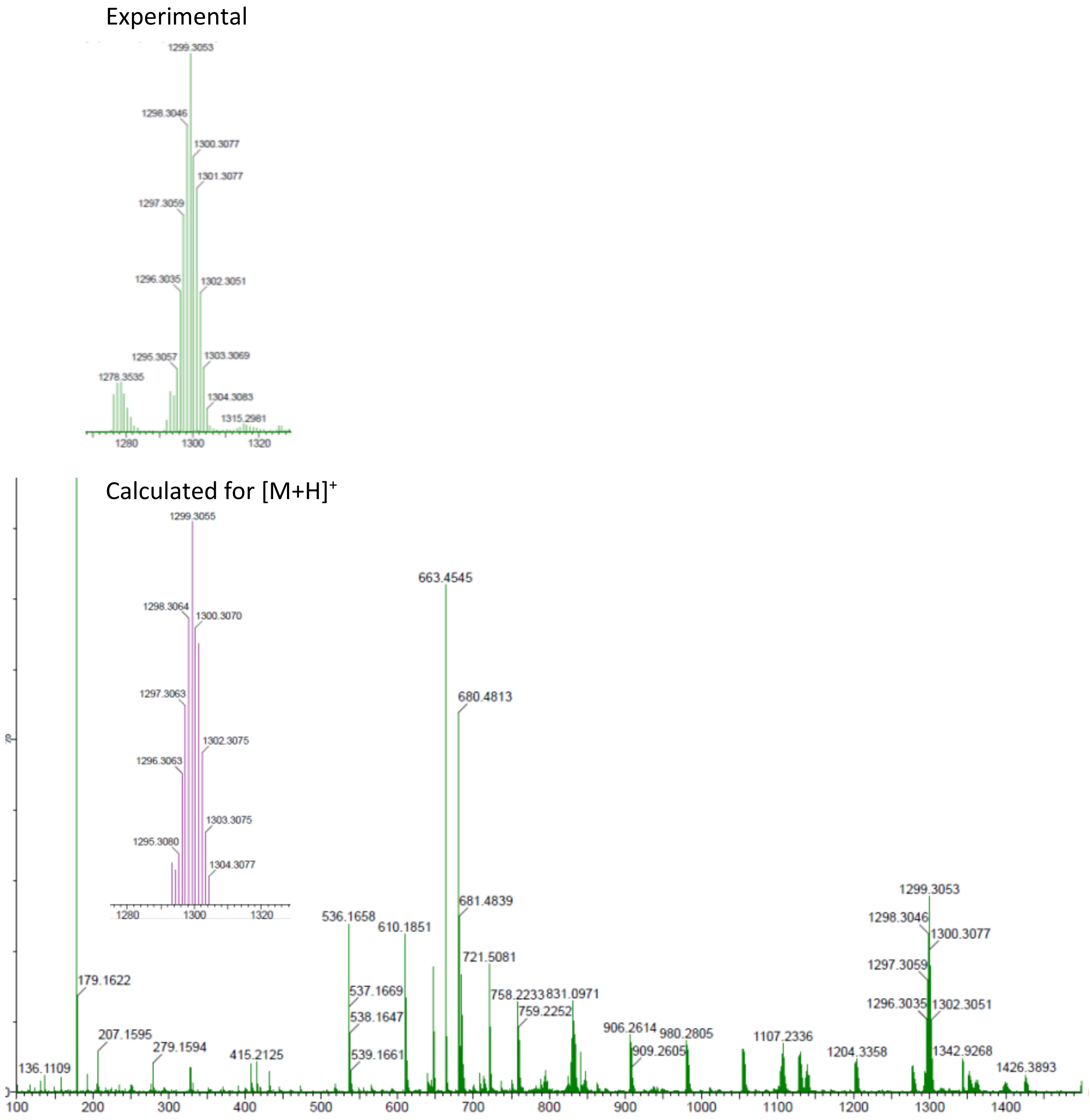

Figure S46. ESI spectrum of **1,3-Cp^{Ar5}[Ru]Tp**

III. Crystallographic data

1. X-ray structure of 3’ (CCDC-2393052)

Crystals of **3’** suitable for single-crystal X-ray analysis were obtained by the slow diffusion of methanol in a concentrated $CHCl_3$ solution of the compound. The single crystal X-ray diffraction data were collected on a Rigaku XtaLAB Synergy-R diffractometer equipped with PhotonJet-R X-ray source (1.2 kW) with multi-layer mirror optics for CuKα radiation and Rigaku HyPix-6000 area detector. The collected X-ray diffraction data were processed using CrysAlisPro software. Using Olex2 software,[1] the structure was solved by SHELXT[2] and refined by full-matrix least-squares on F^2 using the SHELXL.[3] The geometrical restraints, i.e. RIGU on peripheral alkyl chains on the indazole moiety, were used in the refinements of **3’**.

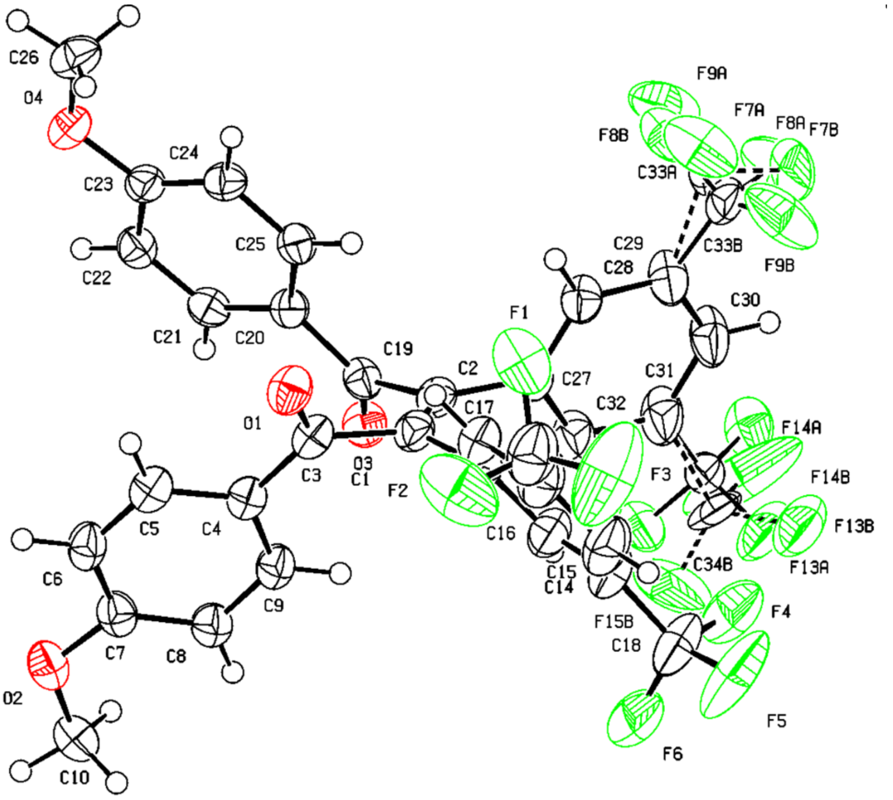

Figure S47. ORTEP of **3’**. Thermal ellipsoids drawn at 50% probability. Solvent molecules are omitted for clarity.

Table S1. Crystal data for compound **3’** (CCDC-2393052):

Empirical formula	$C_{34}H_{20}F_{12}O_4$
CCDC number	2393052
Formula weight	720.50
Temperature [K]	103(2)
Crystal system	monoclinic
Space group (number)	P 1 21/c 1
a [Å]	23.0969(18)
b [Å]	11.0117(9)
c [Å]	12.0108(9)
α [°]	90
β [°]	93.0490(13)
γ [°]	90
Volume [$Å^3$]	3050.5(4)
Z	4
ρ_{calc} [gcm^{-3}]	1.569
μ [mm^{-1}]	0.151
F(000)	1456
Crystal size [mm^3]	0.03×0.09×0.16
Crystal colour	Colourless
Crystal shape	Prism
Radiation	MoK_α (λ=0.71075 Å)
2θ range [°]	3.74 to 50.70
Index ranges	−27 ≤ h ≤ 27<br>−13 ≤ k ≤ 13<br>−14 ≤ l ≤ 13
Reflections collected	41660
Independent reflections	5595<br>R_{int} = 0.0689<br>R_{sigma} = 0.0389
Completeness to<br>θ = 25.242°	99.9
Data / Restraints / Parameters	5595 / 0 / 527
Goodness-of-fit on F^2	1.029
Final *R* indexes<br>[*I*≥2σ(*I*)]	R_1 = 0.0604<br>wR_2 = 0.1405
Final *R* indexes<br>[all data]	R_1 = 0.0830<br>wR_2 = 0.1548
Largest peak/hole [e$Å^{-3}$]	0.41/-0.34

2. X-ray structure of 1,2-Cp^{Ar5}[Ru]Tp (CCDC-2443081)

Single crystals of **1,2-Cp^{Ar5}[Ru]Tp** grown from evaporation of a 1:1 dichloromethane/methanol solution of the complex, were selected in mother liquor from a flask and covered with perfluorated polyether oil on a microscope slide. These solvated crystals were highly unstable, leading very rapidly to desolvation and decomposition of the crystal lattice. An appropriate crystal was quickly selected using a polarizing microscope, fixed on the tip of a MiTeGen© MicroMount, transferred to a goniometer head, and shock cooled by the crystalcooling device. Crystallographic data were collected at 193(2) K on a Bruker-AXS kappa APEX II Quazar diffractometer equipped with a 30W air-cooled microfocus source using Mo K_α radiation (λ=0.71073 Å). Phi- and omega-scans were used. Space group was determined on the basis of systematic absences and intensity statistics. Semi-empirical absorption correction was employed.[4] These structures were solved using an intrinsic phasing method (SHELXT),[2] and refined using the least-squares method on F^2.[3] All non-H atoms were refined with anisotropic displacement parameters. Hydrogen atoms were refined isotropically at calculated positions using a riding model with their isotropic displacement parameters constrained to be equal to 1.5 times the equivalent isotropic displacement parameters of their pivot atoms for terminal sp^3 carbon and 1.2 times for all other carbon atoms. Hydrogen on Boron atom was located by difference Fourier map.

The structure was found to be strongly disordered. Solvent molecules, thioether groups, trifluoromethyl groups and methoxy groups were disordered over two or more positions, for which occupancies were refined. Several restraints (SAME, SIMU, RIGU, ISOR, DANG, FLAT) and equal xyz and U_{ij} constraints (EXYZ and EADP) were also applied to refine some moieties of the molecule and to avoid the collapse of the structure during the least-squares refinement by the large anisotropic displacement parameters. Some bond lengths were restrained with DFIX to suitable target values.

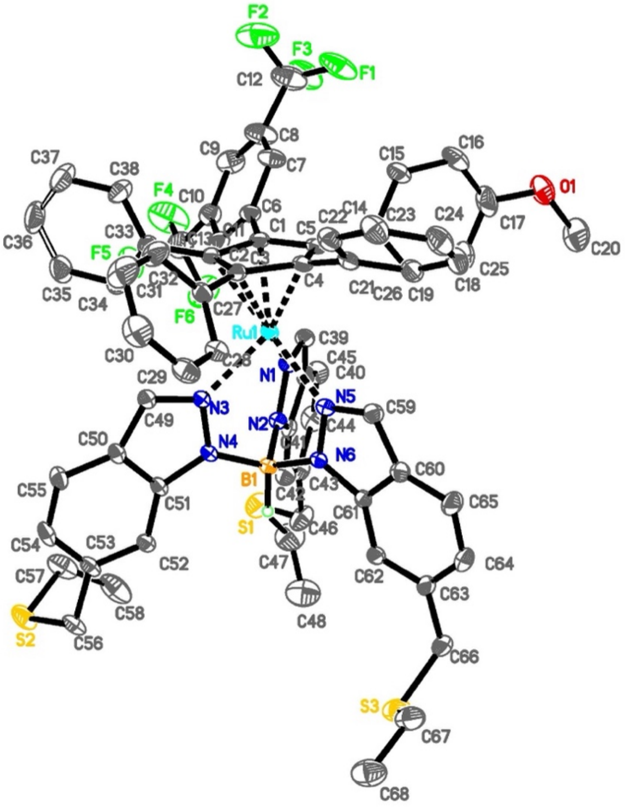

Figure S48. Molecular view of **1,2-Cp^{Ar5}[Ru]Tp**. Thermal ellipsoids drawn at 30% probability, hydrogen atoms, solvent molecules and disordered atoms are omitted for clarity.

Table S2. Crystal data for complex **1,2-Cp^{Ar5}[Ru]Tp** (CCDC-2443081).

Empirical formula	$C_{69}H_{63}BF_6N_6O_2RuS_3$, CH_4O
CCDC number	2443081
Formula weight	1330.31
Temperature [K]	193(2)
Crystal system	triclinic
Space group (number)	$P\overline{1}$ (2)
a [Å]	11.9803(4)
b [Å]	14.3449(5)
c [Å]	20.3176(6)
α [°]	86.5640(11)
β [°]	86.8385(11)
γ [°]	71.1262(11)
Volume [Å^3]	3295.67(19)
Z	2
ρ_{calc} [gcm^{-3}]	1.341
μ [mm^{-1}]	0.399
F(000)	1372
Crystal size [mm^3]	0.04×0.08×0.14
Crystal colour	Orange
Crystal shape	Stick
Radiation	MoK_{α} (λ=0.71073 Å)
2θ range [°]	3.00 to 56.71 (0.75 Å)
Index ranges	$-15 \le h \le 15$<br>$-19 \le k \le 19$<br>$-27 \le l \le 27$
Reflections collected	109420
Independent reflections	16382<br>R_{int} = 0.0556<br>R_{sigma} = 0.0399
Completeness to θ = 25.242°	99.8
Data / Restraints / Parameters	16382 / 817 / 1012
Goodness-of-fit on F^2	1.057
Final R indexes [I≥2σ(I)]	R_1 = 0.0475<br>wR_2 = 0.1259
Final R indexes [all data]	R_1 = 0.0663<br>wR_2 = 0.1388
Largest peak/hole [eÅ^{-3}]	0.97/−0.69
Extinction coefficient	---

3. X-ray structure of 1,3-Cp^{Ar5}[Ru]Tp (CCDC-2443082)

Single crystals of **1,3-Cp^{Ar5}[Ru]Tp** grown by evaporation of a 1:1 dichloromethane/methanol solution of complex, were selected in mother liquor from a flask and covered with perfluorated polyether oil on a microscope slide. These solvated crystals were highly unstable, leading very rapidly to desolvation and decomposition of the crystal lattice. An appropriate crystal was quickly selected using a polarizing microscope, fixed on the tip of a MiTeGen© MicroMount, transferred to a goniometer head, and shock cooled by the crystalcooling device. Crystallographic data were collected at 193(2) K on a Bruker-AXS kappa D8-Venture equipped with a 30W air-cooled microfocus source using Cu K_{α} radiation (λ=1.54178 Å) and a PHOTON III-C14 detector. Phi- and omega-scans were used. Space group was determined on the basis of systematic absences and intensity statistics. Semi-empirical absorption correction was employed.[4] These structures were solved using an intrinsic phasing method (SHELXT),[2] and refined using the least-squares method on F^2.[3] All non-H atoms were refined with anisotropic displacement parameters. Hydrogen atoms were refined isotropically at calculated positions using a riding model with their isotropic displacement parameters constrained to be equal to 1.5 times the equivalent isotropic displacement parameters of their pivot atoms for terminal sp^3 carbon and 1.2 times for all other carbon atoms. Hydrogen on Boron atom was located by difference Fourier map.

The structure was found to be strongly disordered. Solvent molecules, thioether groups, trifluoromethyl groups and methoxy groups were disordered over two or more positions, for which occupancies were refined. Several restraints (SAME, SIMU, RIGU, ISOR, DANG, FLAT) and equal xyz and U_{ij} constraints (EXYZ and EADP) were also applied to refine some moieties of the molecule and to avoid the collapse of the structure during the least-squares refinement by the large anisotropic displacement parameters. Some residual electron density were difficult to modelize and therefore, the SQUEEZE function of PLATON[5] was used to eliminate the contribution of the electron density in the solvent region from the intensity data, and the solvent-free model was employed for the final refinement.

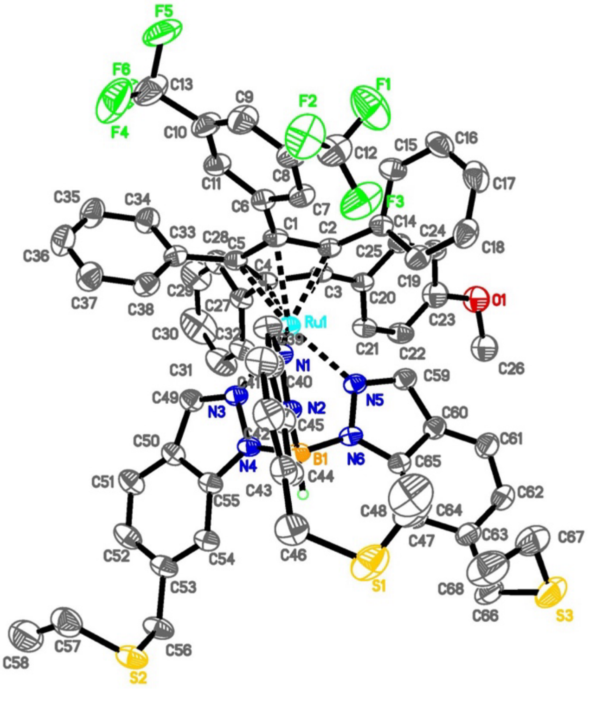

Figure S49. Molecular view of **1,3-Cp^{Ar5}[Ru]Tp**. The thermal ellipsoids are drawn at 30% probability level, hydrogen atoms and disordered atoms are omitted for clarity.

Table S3. Crystal data for complex **1,3-Cp^{Ar5}[Ru]Tp** (CCDC-2443082).

Empirical formula	$C_{68}H_{59}BF_6N_6ORuS_3$ + solvent
CCDC number	2443082
Formula weight	1298.27
Temperature [K]	193(2)
Crystal system	triclinic
Space group (number)	$P\overline{1}$ (2)
a [Å]	14.0201(11)
b [Å]	16.0847(12)
c [Å]	17.0326(14)
α [°]	73.488(4)
β [°]	72.210(4)
γ [°]	84.302(4)
Volume [Å^3]	3506.2(5)
Z	2
ρ_{calc} [gcm^{-3}]	1.23
μ [mm^{-1}]	3.134
F(000)	1336
Crystal size [mm^3]	0.1×0.5×0.6
Crystal colour	Orange
Crystal shape	Plate
Radiation	CuK_α (λ=1.54178 Å)
2θ range [°]	5.66 to 140.57 (0.82 Å)
Index ranges	−17 ≤ h ≤ 17<br>−19 ≤ k ≤ 19<br>−20 ≤ l ≤ 20
Reflections collected	50566
Independent reflections	13257<br>R_{int} = 0.0624<br>R_{sigma} = 0.0594
Completeness to<br>θ = 67.679°	99.4
Data / Restraints / Parameters	13257 / 581 / 911
Goodness-of-fit on F^2	1.036
Final *R* indexes<br>[I≥2σ(I)]	R_1 = 0.0576<br>wR_2 = 0.1603
Final *R* indexes<br>[all data]	R_1 = 0.0624<br>wR_2 = 0.1651
Largest peak/hole [eÅ^{-3}]	1.03/−1.15
Extinction coefficient	0.0015(2)

Table S4. Bond lengths and angles for **3'**.

Atom–Atom	Length [Å]
F2-C17	1.307(4)
F1–C17	1.343(4)
F3-C17	1.326(4)
F7B-C33B	1.290(14)
F4-C18	1.325(5)
F15A C34A	1.352(7)
F13A C34A	1.299(9)
F14A C34A	1.352(10)
F5-C18	1.318(4)
F6 C18	1.312(5)
O4-C23	1.358(3)
O4-C26	1.432(3)
O2-C7	1.360(3)
O2-C10	1.440(3)
O3-C19	1.223(3)
O1-C3	1.228(3)
C23-C24	1.386(4)
C23-C22	1.400(4)
C20-C25	1.399(3)
C20-C21	1.402(4)
C20-C19	1.472(3)
C25-C24	1.381(3)
C4-C9	1.393(4)
C4-C3	1.466(4)
C4-C5	1.400(4)
F9B-C33B	1.321(12)
C7-C6	1.397(4)
C7-C8	1.389(4)
C21-C22	1.368(4)
C1-C3	1.525(4)
C1-C2	1.344(4)
C1-C11	1.489(4)
C6-C5	1.375(4)
C19-C2	1.516(4)
C9-C8	1.386(4)
C2-C27	1.500(4)
C27-C28	1.383(4)
C27-C32	1.391(4)
C28-C29	1.390(4)
C11-C12	1.389(4)
C11-C16	1.396(4)
C12-C13	1.391(4)
C16-C15	1.392(4)
C29-C30	1.387(4)
C29-C33B	1.446(13)
C29-C33A	1.613(15)
C13-C14	1.382(5)
C13-C17	1.494(5)
C32-C31	1.390(4)
C30-C31	1.379(5)
C15-C14	1.381(5)
C15-C18	1.498(5)
C31-C34A	1.485(8)
C31-C34B	1.648(14)
F8B-C33B	1.324(12)
F7A-C33A	1.262(19)
C33A-F9A	1.318(16)
C33A-F8A	1.334(18)
F15B-C34B	1.245(16)
F13B-C34B	1.324(19)
F14B-C34B	1.267(19)
**Atom–Atom–Atom**	**Angle [°]**
C23-O4-C26	117.6(2)
C7-O2-C10	117.4(2)
O4-C23-C24	124.7(2)
O4-C23-C22	114.8(2)
C24-C23-C22	120.5(2)
C25-C20-C21	118.6(2)
C25-C20-C19	122.3(2)
C21-C20-C19	119.0(2)
C24-C25-C20	120.9(2)
C9-C4-C3	122.6(2)
C9-C4-C5	118.2(2)
C5-C4-C3	119.2(2)
C25-C24-C23	119.4(2)
O2-C7-C6	115.6(2)
O2-C7-C8	124.4(2)
C8-C7-C6	120.1(3)
C22-C21-C20	120.9(2)
C21-C22-C23	119.6(2)
C2-C1-C3	120.9(2)
C2-C1-C11	124.5(2)
C11-C1-C3	114.6(2)
C5-C6-C7	120.0(2)
O3-C19-C20	122.6(2)
O3-C19-C2	118.0(2)
C20-C19-C2	119.5(2)
C8-C9-C4	121.6(2)
O1-C3-C4	121.9(2)
O1-C3-C1	117.6(2)
C4-C3-C1	120.5(2)
C6-C5-C4	120.9(2)

C9-C8-C7	119.2(2)
C1-C2-C19	121.2(2)
C1-C2-C27	125.4(2)
C27-C2-C19	113.3(2)
C28-C27-C2	120.8(2)
C28-C27-C32	118.9(3)
C32-C27-C2	120.1(2)
C27-C28-C29	120.4(2)
C12-C11-C1	121.6(2)
C12-C11-C16	118.7(3)
C16-C11-C1	119.6(3)
C11-C12-C13	120.0(3)
C15-C16-C11	120.5(3)
C28-C29-C33B	122.3(5)
C28-C29-C33A	116.5(6)
C30-C29-C28	120.8(3)
C30-C29-C33B	116.5(5)
C30-C29-C33A	120.7(6)
C12-C13-C17	118.5(3)
C14-C13-C12	121.5(3)
C14-C13-C17	119.9(3)
C31-C32-C27	120.3(3)
C31-C30-C29	118.7(3)
C16-C15-C18	117.9(3)
C14-C15-C16	120.7(3)
C14-C15-C18	121.3(3)
C15-C14-C13	118.6(3)
C32-C31-C34A	121.7(3)
C32-C31-C34B	111.7(6)
C30-C31-C32	120.9(3)
C30-C31-C34A	117.2(3)
C30-C31-C34B	123.2(6)
F2-C17-F1	104.4(3)
F2-C17-F3	108.6(3)
F2-C17-C13	113.2(3)

F1-C17-C13	112.1(3)
F3-C17-F1	104.0(3)
F3-C17-C13	113.7(3)
F4-C18-C15	111.2(3)
F5-C18-F4	105.5(3)
F5-C18-C15	113.2(4)
F6-C18-F4	106.0(4)
F6-C18-F5	107.6(4)
F6-C18-C15	112.8(3)
F15A-C34A-F14A	103.2(7)
F15A-C34A-C31	112.9(4)
F13A-C34A-F15A	105.7(6)
F13A-C34A-F14A	108.2(5)
F13A-C34A-C31	108.5(8)
F14A-C34A-C31	117.6(5)
F7B-C33B-F9B	105.3(10)
F7B-C33B-C29	113.6(9)
F7B-C33B-F8B	106.5(10)
F9B-C33B-C29	118.3(8)
F9B-C33B-F8B	104.6(10)
F8B-C33B-C29	107.6(9)
F7A-C33A-C29	111.2(12)
F7A-C33A-F9A	106.7(12)
F7A-C33A-F8A	109.6(13)
F9A-C33A-C29	119.1(10)
F9A-C33A-F8A	103.0(13)
F8A-C33A-C29	106.7(11)
F15B-C34B-C31	120.5(12)
F15B-C34B-F13B	107.7(15)
F15B-C34B-F14B	117.6(15)
F13B-C34B-C31	115.7(11)
F14B-C34B-C31	97.5(12)
F14B-C34B-F13B	94.8(15)

Table S5. Bond lengths and angles for **1,2-Cp^{Ar5}[Ru]Tp.**

**Atom–Atom**	**Length [Å]**
Ru1–N3	2.140(2)
Ru1–N5	2.149(2)
Ru1–C2	2.156(3)
Ru1–N1	2.156(2)
Ru1–C3	2.160(3)
Ru1–C1	2.165(2)
Ru1–C5	2.173(2)
Ru1–C4	2.215(3)
N1–C39	1.326(3)
N1–N2	1.371(3)
N2–C41	1.356(3)
N2–B1	1.536(3)
N3–C49	1.328(3)
N3–N4	1.375(3)
N4–C51	1.362(3)
N4–B1	1.528(4)
N5–C59	1.318(4)
N5–N6	1.381(3)
N6–C61	1.360(3)
N6–B1	1.536(4)
B1–H1	1.11(3)
C1–C2	1.432(4)
C1–C5	1.442(4)
C1–C6	1.485(4)
C2–C3	1.455(4)
C2–C33	1.494(4)
C3–C4	1.441(4)
C3–C27	1.486(4)
C4–C5	1.450(4)
C4–C21	1.491(4)
C5–C14	1.490(4)
C6–C11	1.389(4)
C6–C7	1.394(4)
C7–C8	1.384(5)
C8–C9	1.376(6)
C8–C12	1.513(9)
C12–F2	1.273(9)
C12–F3	1.309(9)
C12–F1	1.354(10)
C9–C10	1.383(5)
C10–C11	1.383(4)
C10–C13	1.479(6)
C13–F4	1.299(7)
C13–F6	1.327(6)
C13–F5	1.361(7)
C14–C15	1.386(4)
C14–C19	1.389(4)
C15–C16	1.399(5)
C16–C17	1.373(6)
C17–C18	1.372(6)
C17–O1	1.382(5)
O1–C20	1.445(9)
C36–C35	1.362(7)
C36–C37	1.368(7)
C18–C19	1.389(5)
C21–C26	1.400(4)
C21–C22	1.400(4)
C22–C23	1.383(5)
C23–C24	1.371(5)
C24–C25	1.371(5)
C25–C26	1.387(5)
C27–C32	1.391(4)
C27–C28	1.399(4)
C28–C29	1.384(4)
C29–C30	1.387(5)
C30–C31	1.370(6)
C31–C32	1.393(5)
C33–C34	1.388(5)
C33–C38	1.390(4)
C34–C35	1.394(5)
C37–C38	1.396(6)
C39–C40	1.407(4)
C40–C41	1.401(4)
C40–C45	1.412(4)
C41–C42	1.400(4)
C42–C43	1.372(4)
C44–C45	1.363(5)
C44–C43	1.414(5)
C43–C46	1.504(5)
C46–S1	1.833(7)
S1–C47	1.753(11)
C47–C48	1.500(14)
C49–C50	1.404(4)
C50–C51	1.405(4)
C50–C55	1.406(4)
C51–C52	1.408(4)
C52–C53	1.381(4)
C53–C54	1.416(4)
C53–C56	1.496(4)
C54–C55	1.368(4)
C56–S2	1.812(3)
S2–C57	1.807(4)
C57–C58	1.491(7)
C59–C60	1.418(4)
C60–C65	1.403(4)
C60–C61	1.404(4)
C61–C62	1.407(4)

C62–C63	1.377(4)
C63–C64	1.414(5)
C63–C66'	1.508(4)
C63–C66	1.508(4)
C64–C65	1.366(5)
C66–S3	1.800(12)
S3–C67	1.811(14)
C67–C68	1.536(15)
O2–C69	1.380(9)
O3–C70	1.390(14)
**Atom–Atom–Atom**	**Angle [°]**
N3–Ru1–N5	81.28(8)
N3–Ru1–C2	96.58(9)
N5–Ru1–C2	156.89(10)
N3–Ru1–N1	87.42(8)
N5–Ru1–N1	81.70(8)
C2–Ru1–N1	121.32(9)
N3–Ru1–C3	98.01(9)
N5–Ru1–C3	117.81(9)
C2–Ru1–C3	39.39(10)
N1–Ru1–C3	160.27(9)
N3–Ru1–C1	127.99(10)
N5–Ru1–C1	150.69(10)
C2–Ru1–C1	38.72(10)
N1–Ru1–C1	96.51(9)
C3–Ru1–C1	65.20(10)
N3–Ru1–C5	161.47(9)
N5–Ru1–C5	113.26(10)
C2–Ru1–C5	65.46(10)
N1–Ru1–C5	105.49(9)
C3–Ru1–C5	65.47(10)
C1–Ru1–C5	38.83(11)
N3–Ru1–C4	131.00(9)
N5–Ru1–C4	99.34(9)
C2–Ru1–C4	64.67(10)
N1–Ru1–C4	141.48(9)
C3–Ru1–C4	38.44(10)
C1–Ru1–C4	64.17(10)
C5–Ru1–C4	38.57(10)
C39–N1–N2	107.7(2)
C39–N1–Ru1	132.15(19)
N2–N1–Ru1	120.09(15)
C41–N2–N1	109.3(2)
C41–N2–B1	130.4(2)
N1–N2–B1	119.1(2)
C49–N3–N4	107.6(2)
C49–N3–Ru1	131.08(18)
N4–N3–Ru1	121.29(16)
C51–N4–N3	109.2(2)
C51–N4–B1	131.8(2)
N3–N4–B1	118.9(2)

C59–N5–N6	107.9(2)
C59–N5–Ru1	132.37(18)
N6–N5–Ru1	119.47(16)
C61–N6–N5	109.1(2)
C61–N6–B1	130.6(2)
N5–N6–B1	120.3(2)
N4–B1–N2	108.6(2)
N4–B1–N6	108.8(2)
N2–B1–N6	107.6(2)
N4–B1–H1	109.7(14)
N2–B1–H1	112.5(14)
N6–B1–H1	109.5(14)
C2–C1–C5	109.0(2)
C2–C1–C6	123.2(3)
C5–C1–C6	127.2(2)
C2–C1–Ru1	70.29(14)
C5–C1–Ru1	70.89(14)
C6–C1–Ru1	131.84(19)
C1–C2–C3	107.6(2)
C1–C2–C33	122.3(2)
C3–C2–C33	129.2(2)
C1–C2–Ru1	70.99(15)
C3–C2–Ru1	70.46(14)
C33–C2–Ru1	132.0(2)
C4–C3–C2	107.7(2)
C4–C3–C27	125.4(2)
C2–C3–C27	126.8(2)
C4–C3–Ru1	72.85(15)
C2–C3–Ru1	70.14(14)
C27–C3–Ru1	124.91(18)
C3–C4–C5	108.3(2)
C3–C4–C21	126.0(2)
C5–C4–C21	124.6(2)
C3–C4–Ru1	68.71(15)
C5–C4–Ru1	69.15(14)
C21–C4–Ru1	137.05(19)
C1–C5–C4	107.2(2)
C1–C5–C14	126.6(2)
C4–C5–C14	125.6(3)
C1–C5–Ru1	70.29(14)
C4–C5–Ru1	72.28(14)
C14–C5–Ru1	129.81(19)
C11–C6–C7	118.1(3)
C11–C6–C1	122.9(3)
C7–C6–C1	118.9(3)
C8–C7–C6	119.8(3)
C9–C8–C7	121.8(3)
C9–C8–C12	120.4(5)
C7–C8–C12	117.6(5)
F2–C12–F3	110.8(8)
F2–C12–F1	102.9(8)
F3–C12–F1	101.2(7)

F2–C12–C8	111.0(7)
F3–C12–C8	114.0(7)
F1–C12–C8	116.0(7)
C8–C9–C10	118.7(3)
C9–C10–C11	120.1(3)
C9–C10–C13	120.4(3)
C11–C10–C13	119.4(3)
C10–C11–C6	121.4(3)
F4–C13–F6	108.9(6)
F4–C13–F5	103.8(6)
F6–C13–F5	100.4(5)
F4–C13–C10	117.3(5)
F6–C13–C10	113.4(4)
F5–C13–C10	111.2(4)
C15–C14–C19	118.2(3)
C15–C14–C5	120.5(3)
C19–C14–C5	121.1(3)
C14–C15–C16	120.5(4)
C17–C16–C15	119.8(4)
C18–C17–C16	120.7(3)
C18–C17–O1	118.0(4)
C16–C17–O1	121.2(4)
C17–O1–C20	121.6(5)
C35–C36–C37	120.7(4)
C17–C18–C19	119.3(4)
C18–C19–C14	121.5(3)
C26–C21–C22	117.2(3)
C26–C21–C4	122.4(3)
C22–C21–C4	119.9(3)
C23–C22–C21	121.1(3)
C24–C23–C22	120.5(3)
C25–C24–C23	119.6(3)
C24–C25–C26	120.6(3)
C25–C26–C21	120.8(3)
C32–C27–C28	118.4(3)
C32–C27–C3	121.1(3)
C28–C27–C3	120.5(2)
C29–C28–C27	121.1(3)
C28–C29–C30	119.4(3)
C31–C30–C29	120.5(3)
C30–C31–C32	120.3(4)
C27–C32–C31	120.3(3)
C34–C33–C38	118.1(3)
C34–C33–C2	123.6(3)
C38–C33–C2	118.0(3)
C33–C34–C35	121.2(3)
C36–C35–C34	119.6(4)
C36–C37–C38	120.1(4)
C33–C38–C37	120.3(4)
N1–C39–C40	110.3(2)

C41–C40–C39	104.7(2)
C41–C40–C45	119.5(3)
C39–C40–C45	135.8(3)
N2–C41–C42	129.9(3)
N2–C41–C40	108.0(2)
C42–C41–C40	122.1(3)
C43–C42–C41	117.6(3)
C45–C44–C43	122.3(3)
C44–C45–C40	118.0(3)
C42–C43–C44	120.5(3)
C42–C43–C46	119.7(4)
C44–C43–C46	119.5(3)
C43–C46–S1	107.2(4)
C47–S1–C46	102.4(6)
C48–C47–S1	117.4(10)
N3–C49–C50	110.6(2)
C49–C50–C51	104.7(2)
C49–C50–C55	135.8(3)
C51–C50–C55	119.5(3)
N4–C51–C50	107.9(2)
N4–C51–C52	130.1(2)
C50–C51–C52	122.0(2)
C53–C52–C51	117.5(3)
C52–C53–C54	120.3(3)
C52–C53–C56	120.0(3)
C54–C53–C56	119.7(3)
C55–C54–C53	122.3(3)
C54–C55–C50	118.3(3)
C53–C56–S2	112.8(2)
C57–S2–C56	100.80(16)
C58–C57–S2	114.7(3)
N5–C59–C60	110.5(2)
C65–C60–C61	119.9(3)
C65–C60–C59	135.7(3)
C61–C60–C59	104.4(2)
N6–C61–C60	108.1(2)
N6–C61–C62	130.3(2)
C60–C61–C62	121.6(2)
C63–C62–C61	117.7(3)
C62–C63–C64	120.4(3)
C62–C63–C66	120.6(3)
C64–C63–C66	119.0(3)
C65–C64–C63	122.3(3)
C64–C65–C60	118.1(3)
C63–C66–S3	118.1(7)
C66–S3–C67	100.6(9)
C68–C67–S3	108.1(14)

Table S6. Bond lengths and angles for **1,3-Cp^{Ar5}[Ru]Tp.**

Atom–Atom	Length [Å]
Ru1–N5	2.153(3)
Ru1–N3	2.153(3)
Ru1–N1	2.155(3)
Ru1–C5	2.169(3)
Ru1–C2	2.176(3)
Ru1–C4	2.178(3)
Ru1–C3	2.186(3)
Ru1–C1	2.194(3)
N1–C39	1.327(5)
N1–N2	1.366(4)
N2–C45	1.370(4)
N2–B1	1.547(5)
N3–C49	1.314(5)
N3–N4	1.376(4)
N4–C55	1.369(4)
N4–B1	1.537(5)
N5–C59	1.329(5)
N5–N6	1.371(4)
N6–C65	1.363(4)
N6–B1	1.522(5)
C1–C2	1.449(5)
C1–C5	1.455(5)
C1–C6	1.492(4)
C2–C3	1.452(4)
C2–C14	1.481(5)
C3–C4	1.435(5)
C3–C20	1.482(5)
C4–C5	1.466(5)
C4–C27	1.475(5)
C5–C33	1.482(5)
C6–C11	1.379(5)
C6–C7	1.404(5)
C7–C8	1.400(5)
C8–C9	1.374(7)
C8–C12	1.479(6)
C9–C10	1.384(7)
C10–C11	1.386(6)
C10–C13	1.514(10)
C13–F6	1.325(10)
C13–F5	1.342(10)
C13–F4	1.353(11)
C12–F1	1.244(9)
C12–F3	1.294(8)
C12–F2	1.361(8)
C14–C19	1.394(5)
C14–C15	1.396(5)
C15–C16	1.383(6)
C16–C17	1.385(8)
C17–C18	1.363(8)
C18–C19	1.387(6)
C20–C21	1.398(5)
C20–C25	1.402(5)
C21–C22	1.381(6)
C22–C23	1.389(6)
C23–O1	1.376(6)
C23–C24	1.378(6)
O1–C26	1.446(9)
C30–C31	1.359(10)
C30–C29	1.411(10)
C24–C25	1.383(5)
C27–C32	1.398(5)
C27–C28	1.400(5)
C28–C29	1.358(6)
C31–C32	1.372(6)
C33–C38	1.386(5)
C33–C34	1.394(5)
C34–C35	1.379(6)
C35–C36	1.371(7)
C36–C37	1.368(7)
C37–C38	1.386(6)
C39–C40	1.399(5)
C40–C45	1.408(5)
C40–C41	1.413(5)
C41–C42	1.376(6)
C42–C43	1.402(7)
C43–C44	1.375(5)
C43–C46	1.516(6)
C46–S1	1.826(6)
S1–C47	1.662(11)
C47–C48	1.491(13)
C44–C45	1.401(5)
C49–C50	1.403(5)
C50–C55	1.408(5)
C50–C51	1.413(5)
C51–C52	1.360(6)
C52–C53	1.405(7)
C53–C54	1.394(5)
C53–C56	1.509(5)
C54–C55	1.402(5)
C56–S2	1.815(4)
S2–C57	1.767(6)
C57–C58	1.458(10)
C59–C60	1.414(5)

C60–C65	1.398(5)
C60–C61	1.410(5)
C61–C62	1.366(6)
C62–C63	1.422(6)
C63–C64	1.375(6)
C63–C66	1.510(6)
C64–C65	1.411(5)
C66–S3	1.813(5)
S3–C67	1.812(10)
C67–C68	1.512(14)
B1–H1	1.03(4)
**Atom–Atom–Atom**	**Angle [°]**
N5–Ru1–N3	85.92(11)
N5–Ru1–N1	84.24(10)
N3–Ru1–N1	79.78(10)
N5–Ru1–C5	159.58(12)
N3–Ru1–C5	101.19(11)
N1–Ru1–C5	115.73(11)
N5–Ru1–C2	103.19(11)
N3–Ru1–C2	163.48(12)
N1–Ru1–C2	114.48(11)
C5–Ru1–C2	65.75(12)
N5–Ru1–C4	121.00(11)
N3–Ru1–C4	98.23(11)
N1–Ru1–C4	154.64(12)
C5–Ru1–C4	39.43(12)
C2–Ru1–C4	65.29(12)
N5–Ru1–C3	95.21(11)
N3–Ru1–C3	127.61(11)
N1–Ru1–C3	152.57(11)
C5–Ru1–C3	65.22(12)
C2–Ru1–C3	38.89(12)
C4–Ru1–C3	38.38(13)
N5–Ru1–C1	139.08(12)
N3–Ru1–C1	134.89(12)
N1–Ru1–C1	98.38(11)
C5–Ru1–C1	38.94(12)
C2–Ru1–C1	38.73(13)
C4–Ru1–C1	64.99(11)
C3–Ru1–C1	64.49(12)
C39–N1–N2	107.3(3)
C39–N1–Ru1	132.7(2)
N2–N1–Ru1	119.9(2)
N1–N2–C45	109.7(3)
N1–N2–B1	120.5(2)
C45–N2–B1	129.6(3)
C49–N3–N4	108.2(3)
C49–N3–Ru1	131.3(2)
N4–N3–Ru1	120.5(2)

C55–N4–N3	108.7(3)
C55–N4–B1	131.3(3)
N3–N4–B1	119.6(3)
C59–N5–N6	107.8(3)
C59–N5–Ru1	131.1(2)
N6–N5–Ru1	121.0(2)
C65–N6–N5	109.2(3)
C65–N6–B1	130.8(3)
N5–N6–B1	119.7(3)
C2–C1–C5	108.6(3)
C2–C1–C6	124.6(3)
C5–C1–C6	125.9(3)
C2–C1–Ru1	69.95(17)
C5–C1–Ru1	69.59(17)
C6–C1–Ru1	134.5(2)
C1–C2–C3	107.3(3)
C1–C2–C14	123.2(3)
C3–C2–C14	128.7(3)
C1–C2–Ru1	71.32(18)
C3–C2–Ru1	70.92(17)
C14–C2–Ru1	130.8(2)
C4–C3–C2	108.9(3)
C4–C3–C20	124.9(3)
C2–C3–C20	125.6(3)
C4–C3–Ru1	70.54(17)
C2–C3–Ru1	70.19(17)
C20–C3–Ru1	131.8(2)
C3–C4–C5	108.0(3)
C3–C4–C27	126.8(3)
C5–C4–C27	124.6(3)
C3–C4–Ru1	71.08(17)
C5–C4–Ru1	69.94(17)
C27–C4–Ru1	131.8(2)
C1–C5–C4	107.1(3)
C1–C5–C33	124.7(3)
C4–C5–C33	127.9(3)
C1–C5–Ru1	71.46(18)
C4–C5–Ru1	70.64(17)
C33–C5–Ru1	128.1(2)
C11–C6–C7	118.1(3)
C11–C6–C1	120.8(3)
C7–C6–C1	120.9(3)
C8–C7–C6	120.2(4)
C9–C8–C7	120.7(4)
C9–C8–C12	119.9(4)
C7–C8–C12	119.3(4)
C8–C9–C10	118.8(4)
C9–C10–C11	121.0(4)
C9–C10–C13	117.0(5)
C11–C10–C13	121.6(5)

F6–C13–F5	103.6(9)
F6–C13–F4	110.5(10)
F5–C13–F4	106.0(9)
F6–C13–C10	111.8(8)
F5–C13–C10	110.1(8)
F4–C13–C10	114.2(8)
C6–C11–C10	121.0(4)
F1–C12–F3	112.0(8)
F1–C12–F2	103.1(7)
F3–C12–F2	104.0(7)
F1–C12–C8	113.7(6)
F3–C12–C8	112.8(5)
F2–C12–C8	110.3(5)
C19–C14–C15	118.6(3)
C19–C14–C2	122.2(3)
C15–C14–C2	119.0(3)
C16–C15–C14	120.7(4)
C15–C16–C17	119.3(4)
C18–C17–C16	121.0(4)
C17–C18–C19	120.0(5)
C18–C19–C14	120.4(4)
C21–C20–C25	117.4(3)
C21–C20–C3	122.8(3)
C25–C20–C3	119.8(3)
C22–C21–C20	121.7(3)
C21–C22–C23	119.9(4)
O1–C23–C24	114.6(5)
O1–C23–C22	125.2(5)
C24–C23–C22	119.2(4)
C23–O1–C26	116.5(5)
C24–C25–C20	120.7(3)
C32–C27–C28	117.1(3)
C32–C27–C4	123.5(3)
C28–C27–C4	119.3(3)
C29–C28–C27	121.5(5)
C28–C29–C30	119.7(5)
C30–C31–C32	120.3(5)
C31–C32–C27	121.6(5)
C38–C33–C34	117.8(4)
C38–C33–C5	121.4(3)
C34–C33–C5	120.7(3)
C35–C34–C33	120.9(4)
C36–C35–C34	120.4(4)
C37–C36–C35	119.7(4)
C36–C37–C38	120.5(4)
C37–C38–C33	120.7(4)
N1–C39–C40	111.0(3)
C39–C40–C45	104.8(3)
C39–C40–C41	135.7(4)
C45–C40–C41	119.5(4)

C42–C41–C40	117.8(4)
C41–C42–C43	121.9(4)
C44–C43–C42	121.7(4)
C44–C43–C46	119.1(4)
C42–C43–C46	119.2(4)
C43–C46–S1	113.9(3)
C47–S1–C46	100.3(4)
C48–C47–S1	123.0(7)
C43–C44–C45	116.9(4)
N2–C45–C44	130.6(3)
N2–C45–C40	107.1(3)
C44–C45–C40	122.3(3)
N3–C49–C50	110.8(3)
C49–C50–C55	104.7(3)
C49–C50–C51	136.3(4)
C55–C50–C51	118.9(3)
C52–C51–C50	118.5(4)
C51–C52–C53	122.6(4)
C54–C53–C52	120.5(3)
C54–C53–C56	119.8(4)
C52–C53–C56	119.7(4)
C53–C54–C55	117.0(4)
N4–C55–C54	129.9(3)
N4–C55–C50	107.6(3)
C54–C55–C50	122.5(3)
C53–C56–S2	114.6(3)
C57–S2–C56	103.3(3)
C58–C57–S2	114.8(5)
N5–C59–C60	110.1(3)
C65–C60–C61	120.1(3)
C65–C60–C59	104.7(3)
C61–C60–C59	135.2(4)
C62–C61–C60	118.1(4)
C61–C62–C63	121.8(4)
C64–C63–C62	120.9(4)
C64–C63–C66	119.2(4)
C62–C63–C66	119.9(4)
C63–C64–C65	117.3(4)
N6–C65–C60	108.2(3)
N6–C65–C64	130.0(3)
C60–C65–C64	121.8(3)
C63–C66–S3	112.4(3)
C67–S3–C66	103.6(4)
C68–C67–S3	108.5(8)
N6–B1–N4	108.9(3)
N6–B1–N2	108.0(3)
N4–B1–N2	107.3(3)
N6–B1–H1	108(2)
N4–B1–H1	111(2)
N2–B1–H1	113(2)

IV. DFT calculations

Orca 5.0.4. was used to optimize the geometry using BP86 functional and def2/J as the basis set. The dispersion correction D3BJ was included in energy calculation corresponding to the **3'-E** and **3'-Z** isomers.

From the calculations given Tables S7 to S10, we found that for the **3'-E** isomer the total energy is E = -2808.016520657799 E_h and for the **3'-Z** isomer the total energy is E = -2808.022042401885 E_h. In consequence, the difference between the *E* and *Z* isomers is 14.5 kJ.mol^{-1} with the Z-isomer the more stable.

1. Optimized coordinates and associated vibrational frequencies of the **1,2-Cp^{Ar5}** anion

Table S7. Optimized coordinates for the calculated geometry of the **1,2-Cp^{Ar5}** anion.

C	2.99446292141330	0.49988251922954	1.37562293909679
O	4.21794041045318	0.01071436722285	0.84676059347805
C	4.14304681728542	-1.06131907706949	-0.02250982583414
C	5.36143337375719	-1.51717677338321	-0.54581387223123
C	5.38551188972834	-2.59561758946168	-1.42477412742669
C	4.20470273281433	-3.26039772228092	-1.82739079032027
C	4.23585240784118	-4.40976824208393	-2.75150866709204
C	3.64313134144798	-5.68136603850248	-2.51361761665905
C	2.86953068885545	-6.06191656332998	-1.32003882797923
C	3.34214305052170	-5.79190917212469	-0.01613814892173
C	2.60739921918695	-6.14878850210392	1.11543998182336
C	1.37393157705068	-6.79670758394504	0.98674707835963
C	0.88807954425631	-7.07742450779657	-0.29495122032215
C	1.62035774006859	-6.71357085903931	-1.42593037164639
C	3.92372926067056	-6.52194437579694	-3.64199644377543
C	3.48219128785274	-7.91645849748254	-3.81133090155264
C	3.65089788071073	-8.87236549366887	-2.78489151592758
C	3.23357113167737	-10.19459301932595	-2.94320083015839
C	2.63675493333026	-10.61301757904099	-4.13749499200873

C	2.46116137013148	-9.68338817151208	-5.16816223960457
C	2.87264423601587	-8.35969271777003	-5.00615214753376
C	4.70045352447265	-5.77225541424618	-4.56943196736361
C	5.18674574013738	-6.25455028594565	-5.87259365211003
C	4.99747995685807	-5.50673450285967	-7.05640994252247
C	5.46275265905225	-5.96302378551495	-8.29078062192968
C	6.12601315936807	-7.19062818596827	-8.38916392806662
C	6.31930257235284	-7.95072192448373	-7.23052470451220
C	5.86239617470150	-7.48924144835284	-5.99562329731255
C	4.89369632953556	-4.45925526376240	-4.02302369034234
C	5.64372594686104	-3.36887352822994	-4.64052673752124
C	5.17287761983551	-2.03416804494210	-4.61228214477720
C	5.89312911133359	-0.99400498250384	-5.20265590603010
C	5.38237354859275	0.41946408766914	-5.12724327536277
F	4.04006689362243	0.49321909847009	-4.91919640120157
F	5.97060816570013	1.12971938219259	-4.11251349138859
F	5.64028625500063	1.12668152206795	-6.27257463653178
C	7.11364281650257	-1.23312101449267	-5.84340567718585
C	7.59410334045318	-2.54820426432835	-5.88028209143994
C	8.89815081436145	-2.80727129705709	-6.58469061271908
F	8.78897480505734	-2.66132712641148	-7.94453843589353
F	9.87693193864176	-1.92645327503003	-6.19775853355668
F	9.39116335618936	-4.05364913071189	-6.36820685644653
C	6.87976083113572	-3.59368719147518	-5.29564392494108
C	2.99781920853021	-2.77752155680244	-1.29007128343980
C	2.95515319589504	-1.69987557391295	-0.39811784314881
H	3.26050681641321	1.34416184695107	2.02390462110182

H	2.47053930548173	-0.26715018505213	1.97332394958814
H	2.31700293058266	0.85272126652084	0.57819103351939
H	6.28311187854851	-1.01534250720562	-0.24607635719864
H	6.34430996291914	-2.94759005868873	-1.80873366959193
H	4.30838358072449	-5.29922068282389	0.09904126796210
H	3.00660022018326	-5.92563953121506	2.10793088364100
H	0.80058497024657	-7.07973582963431	1.87205640759063
H	-0.07450330020153	-7.58009348529066	-0.41691565637281
H	1.22377799338427	-6.92881395802681	-2.41905431513071
H	4.12536749465770	-8.56264957404191	-1.85273206753294
H	3.38252409321044	-10.90678305225125	-2.12790853598271
H	2.31298746890046	-11.64838050924157	-4.26301543612528
H	1.99293589240987	-9.98992829240810	-6.10671534745293
H	2.71858487099615	-7.64253460347696	-5.81357722452763
H	4.46433291517975	-4.55649252264950	-6.99751305484870
H	5.29943320989414	-5.35661831699159	-9.18483401889765
H	6.48968095118905	-7.54829837455159	-9.35452950790877
H	6.84006097222656	-8.90963113308488	-7.28692890724584
H	6.03119704638495	-8.08496571263470	-5.09759172182753
H	4.21590814961318	-1.82289790944865	-4.13834820738279
H	7.67300585944495	-0.41981525492385	-6.30316148299482
H	7.28508447185538	-4.60331455620079	-5.32476978475891
H	2.06325108165003	-3.25988464815546	-1.57996656395198
H	1.99122938484404	-1.36835310957540	-0.01248470169001

Table S8. Associated vibrational frequencies for the optimized geometry of the **1,2-Cp^{Ar5}** anion.

Mode	freq (cm^{-1})	Mode	freq (cm^{-1})	Mode	freq (cm^{-1})
7:	13.32	31:	217.39	55:	493.67
8:	16.86	32:	221.89	56:	498.57
9:	18.08	33:	226.62	57:	504.89
10:	24.42	34:	236.83	58:	509.00
11:	25.93	35:	240.28	59:	510.94
12:	34.18	36:	244.59	60:	524.32
13:	39.01	37:	260.31	61:	536.97
14:	46.32	38:	278.77	62:	546.37
15:	48.85	39:	281.22	63:	578.35
16:	54.21	40:	310.26	64:	599.19
17:	60.59	41:	316.13	65:	609.51
18:	66.85	42:	335.80	66:	611.39
19:	77.65	43:	349.25	67:	614.34
20:	88.16	44:	361.36	68:	614.92
21:	93.77	45:	381.79	69:	629.53
22:	95.35	46:	392.81	70:	641.01
23:	99.26	47:	402.10	71:	644.24
24:	116.83	48:	404.05	72:	655.20
25:	131.84	49:	405.78	73:	667.75
26:	158.39	50:	411.91	74:	672.72
27:	164.75	51:	418.03	75:	679.62
28:	175.98	52:	429.13	76:	680.64
29:	183.64	53:	459.19	77:	683.60
30:	196.27	54:	480.00	78:	694.89

79:	698.10
80:	702.55
81:	705.89
82:	723.65
83:	732.42
84:	743.32
85:	749.47
86:	769.30
87:	778.49
88:	791.43
89:	807.03
90:	813.34
91:	814.23
92:	815.41
93:	817.96
94:	822.06
95:	827.22
96:	863.67
97:	864.84
98:	870.00
99:	886.65
100:	901.78
101:	904.84
102:	916.48
103:	917.44
104:	918.80
105:	920.30
106:	920.66
107:	921.25
108:	922.36
109:	936.10
110:	959.16
111:	978.79
112:	986.10
113:	986.79
114:	987.34
115:	997.53
116:	1018.00
117:	1021.99
118:	1023.48
119:	1035.69
120:	1037.26
121:	1041.49
122:	1050.72
123:	1062.51
124:	1063.28
125:	1064.28
126:	1066.29
127:	1069.91
128:	1091.45
129:	1101.10
130:	1105.58
131:	1128.41
132:	1137.58
133:	1138.56
134:	1139.66
135:	1139.89
136:	1140.57
137:	1156.76
138:	1161.94
139:	1162.60
140:	1163.51
141:	1163.82
142:	1220.22
143:	1223.03
144:	1226.44
145:	1231.84
146:	1261.78
147:	1276.03
148:	1281.63
149:	1284.63
150:	1287.40
151:	1301.85
152:	1314.06
153:	1323.49
154:	1324.07
155:	1325.70
156:	1327.39
157:	1348.15
158:	1353.51
159:	1393.05

160: 1403.14	177: 1554.70	194: 3096.99
161: 1409.10	178: 1555.01	195: 3097.84
162: 1414.66	179: 1566.67	196: 3098.08
163: 1419.96	180: 1575.32	197: 3099.40
164: 1426.05	181: 1582.68	198: 3103.33
165: 1430.09	182: 1583.69	199: 3108.27
166: 1432.47	183: 1588.13	200: 3110.01
167: 1434.73	184: 1594.80	201: 3110.84
168: 1444.62	185: 2913.37	202: 3112.91
169: 1455.55	186: 2963.85	203: 3113.33
170: 1456.86	187: 3043.69	204: 3113.49
171: 1477.57	188: 3071.22	205: 3113.76
172: 1480.46	189: 3071.58	206: 3121.89
173: 1487.26	190: 3073.86	207: 3134.05
174: 1498.77	191: 3080.45	208: 3138.78
175: 1553.33	192: 3080.75	209: 3141.83
176: 1554.14	193: 3082.48	

2. Optimized coordinates and associated vibrational frequencies of the **1,3-Cp^{Ar5}** anion

Table S9. Optimized coordinates for the calculated geometry of the **1,3-Cp^{Ar5}** anion.

C	-8.01537477301033	-4.74923703249494	1.10479076943410
O	-6.83894350869927	-4.46305488968050	1.84524975040439
C	-6.17106070768949	-3.29113631687463	1.53556804734008
C	-6.57540569735953	-2.38203318543542	0.55105339411963
C	-5.81935280818785	-1.22671500013642	0.31994189285663
C	-4.65535462339534	-0.93162303771941	1.05272346255055
C	-3.87007585950370	0.29226262770237	0.80579046530266
C	-2.46262942387826	0.35458904064999	0.60677676999271
C	-1.54083800233304	-0.79299624928590	0.64181810976348
C	-0.36755113428273	-0.77814439034815	1.42910455955701
C	0.50545144168452	-1.86683277744448	1.45894351395449
C	0.23200888317697	-3.01605395311736	0.70993770254054
C	-0.92746939664375	-3.05518980521664	-0.07209524984612
C	-1.79488110795694	-1.96283006122939	-0.10831306713047
C	-2.10347245819447	1.73124288007327	0.41776035492824
C	-0.75516437579977	2.24480495467825	0.19139903011097
C	-0.29017259306734	3.41982111188007	0.83100254802186
C	1.00087357414734	3.90410091184051	0.61678080931765
C	1.46311723392835	5.16702270288707	1.29158685301401
F	1.43958993102132	6.24682032059960	0.44603018615739
F	0.70870104659806	5.50668714687328	2.36991880381720
F	2.75782263724390	5.07298984358247	1.73388660109922
C	1.88614357026573	3.24632667778123	-0.24545265072725
C	1.44163628419888	2.08497906839641	-0.88810391841642

C	2.39831522443099	1.35359438679490	-1.79022513479830
F	3.14449229473050	2.20999188839006	-2.55839139552749
F	3.30559385288518	0.60483066719315	-1.08456086901186
F	1.78095614108634	0.49997630069343	-2.64910345545218
C	0.15353097742466	1.59139611582119	-0.67628462252908
C	-3.30162993862697	2.51302421453763	0.49487937856088
C	-3.40311745651295	3.97068950333223	0.31403021060034
C	-2.80151736930320	4.62166086287842	-0.78629038549389
C	-2.89347687447007	6.00394600305930	-0.95667247146815
C	-3.60018101559890	6.78608657592134	-0.03728067902580
C	-4.20918607757895	6.16166265217536	1.05672992809226
C	-4.10877609693463	4.78086520153765	1.23114942614242
C	-4.39050801733049	1.62496361049593	0.73296551946734
C	-5.80713847391928	2.00596726979771	0.86002071751210
C	-6.60982428302540	1.52164982810742	1.91714752223963
C	-7.95325116027206	1.88016401199860	2.03717131668012
C	-8.54455394668084	2.74237448704388	1.10716841665079
C	-7.76784976827737	3.23590852919197	0.05339686310013
C	-6.42634148646702	2.87128062357842	-0.06949917137902
C	-4.27764149267710	-1.86874240020430	2.04119150348128
C	-5.01278811119007	-3.02567713355537	2.27966185421806
H	-8.39631418153528	-5.70119203908078	1.49574819719094
H	-7.80420769608489	-4.85801805324769	0.02600874834185
H	-8.78391437496984	-3.96718133923489	1.23780465731614
H	-7.46899176920846	-2.55964102950644	-0.04723415653714
H	-6.14298171378201	-0.53258901771213	-0.45684961671899
H	-0.15222044957035	0.10468693033113	2.03307039248775

H	1.40424907010653	-1.81880632845698	2.07817710827375
H	0.91437850738708	-3.86807269591223	0.73343603834082
H	-1.15480064262087	-3.94266019262838	-0.66758367122574
H	-2.68862550476623	-2.00048158172833	-0.73267101037766
H	-0.95213932562434	3.94065366705202	1.52047173224797
H	2.89267279533431	3.62686419150803	-0.41146385536941
H	-0.17165935886400	0.69509260165660	-1.20128859605781
H	-2.26344791354979	4.02027194989943	-1.52076285691402
H	-2.41327305959509	6.47328654378581	-1.81849281338944
H	-3.67260846985345	7.86745360033512	-0.16966563759889
H	-4.76121409088976	6.75716800866925	1.78784551937137
H	-4.57687213830015	4.30807088365933	2.09571518806476
H	-6.15897313175172	0.85786412265652	2.65605960671052
H	-8.54198681315129	1.48776747448895	2.87002201227628
H	-9.59486834162239	3.02575462911601	1.20268534070463
H	-8.21232541514818	3.90759441148818	-0.68511269038807
H	-5.83575662644557	3.25411844161671	-0.90279269058570
H	-3.38530437560806	-1.67343117095848	2.63726926910143
H	-4.70663003384136	-3.73791779454711	3.04812057451244

Table S10. Associated vibrational frequencies for the optimized geometry of the **1,3-Cp^{Ar5}** anion.

Mode	freq (cm^{-1})	Mode	freq (cm^{-1})	Mode	freq (cm^{-1})
7:	14.81	11:	27.92	15:	50.08
8:	17.98	12:	33.53	16:	54.10
9:	19.90	13:	40.89	17:	57.84
10:	25.35	14:	44.32	18:	66.93

19:	74.56	46:	398.55	73:	661.63
20:	86.39	47:	402.59	74:	673.57
21:	92.91	48:	403.47	75:	680.09
22:	96.40	49:	404.92	76:	680.90
23:	99.52	50:	411.89	77:	683.50
24:	121.07	51:	416.93	78:	694.15
25:	130.15	52:	432.34	79:	696.19
26:	157.84	53:	456.52	80:	699.36
27:	160.65	54:	480.63	81:	705.19
28:	176.19	55:	492.18	82:	727.32
29:	189.64	56:	498.69	83:	735.79
30:	198.13	57:	503.49	84:	742.87
31:	212.50	58:	507.99	85:	752.40
32:	225.88	59:	512.49	86:	771.46
33:	227.97	60:	529.69	87:	777.26
34:	233.52	61:	534.54	88:	782.93
35:	239.41	62:	542.65	89:	811.57
36:	246.02	63:	580.22	90:	813.53
37:	257.09	64:	603.16	91:	815.13
38:	280.26	65:	606.33	92:	815.60
39:	286.26	66:	611.67	93:	817.67
40:	307.77	67:	614.57	94:	819.86
41:	316.61	68:	615.39	95:	827.92
42:	331.41	69:	628.24	96:	864.05
43:	349.73	70:	641.17	97:	866.37
44:	363.32	71:	649.34	98:	870.78
45:	377.99	72:	654.92	99:	886.89

100: 902.14
101: 903.69
102: 916.63
103: 918.40
104: 919.04
105: 920.53
106: 921.00
107: 921.79
108: 921.91
109: 936.36
110: 958.65
111: 978.75
112: 986.41
113: 986.87
114: 987.39
115: 997.14
116: 1019.00
117: 1021.77
118: 1023.05
119: 1036.77
120: 1037.75
121: 1040.75
122: 1050.92
123: 1062.57
124: 1062.91
125: 1063.95
126: 1066.59
127: 1069.69
128: 1092.16
129: 1100.95
130: 1104.94
131: 1128.43
132: 1137.70
133: 1138.58
134: 1139.81
135: 1140.41
136: 1140.70
137: 1157.15
138: 1161.66
139: 1162.50
140: 1162.89
141: 1163.64
142: 1218.65
143: 1222.83
144: 1226.68
145: 1231.06
146: 1261.71
147: 1275.76
148: 1281.19
149: 1284.77
150: 1287.28
151: 1305.03
152: 1311.96
153: 1322.68
154: 1324.16
155: 1325.71
156: 1326.79
157: 1347.62
158: 1353.04
159: 1392.97
160: 1403.69
161: 1410.06
162: 1413.73
163: 1420.11
164: 1427.41
165: 1429.29
166: 1433.52
167: 1434.22
168: 1442.46
169: 1455.11
170: 1456.95
171: 1478.07
172: 1481.07
173: 1486.23
174: 1498.70
175: 1553.96
176: 1554.22
177: 1554.87
178: 1554.95
179: 1566.47
180: 1575.25

181: 1582.55

182: 1583.44

183: 1588.37

184: 1594.41

185: 2911.77

186: 2961.11

187: 3042.14

188: 3071.16

189: 3073.93

190: 3074.04

191: 3080.36

192: 3082.66

193: 3082.74

194: 3096.94

195: 3097.91

196: 3099.25

197: 3099.50

198: 3102.99

199: 3108.19

200: 3108.55

201: 3110.72

202: 3112.47

203: 3112.80

204: 3113.56

205: 3115.34

206: 3120.89

207: 3135.48

208: 3139.88

209: 3140.29

3. Optimized coordinates and associated vibrational frequencies of the **3'-E** isomer

Table S11. optimized coordinates for the calculated geometry of the **3'-E** isomer.

C	1.04891743314493	0.45753533056419	-0.97198963685295
C	1.00504334940576	1.80656819992766	-1.07945380153227
C	2.26830364959656	2.64747348710888	-0.97152985903422
O	3.12786050545997	2.54741426699170	-1.84368031534555
C	3.04710316739216	0.14800742388836	0.51871455508866
C	4.16515877716181	-0.58728063421513	0.91628508271802
C	2.24619826007626	-0.29162404700533	-0.54384027485227
C	2.57579229870478	-1.50126709839166	-1.18088869092130
C	3.69480473069690	-2.22620234078987	-0.77987037642838
C	4.50077736556337	-1.77619024001537	0.27149919000856
H	5.37366024056937	-2.34618884650689	0.58380245414702
H	1.96637753018252	-1.85934205638250	-2.00997395480305
C	4.98272531603994	-0.07259773521014	2.07535835668426
C	2.37934743293221	3.58358943152571	0.16501443377878
C	1.42689441414639	3.61874346393071	1.20352011323638
C	1.57797190776408	4.48074374233496	2.27623249356746
C	3.48969805842763	4.43956358686856	0.23822826168670
C	3.65445956912681	5.31229616519004	1.30609425519882
C	2.69366252168574	5.33747057634847	2.33457686477935
H	0.84858315969735	4.51729491880046	3.08513209602251
O	2.75551389621946	6.14830587905953	3.42063331823963
H	4.22603527999492	4.39769609505889	-0.56500093721904
H	4.52471430409780	5.96483349854430	1.34002362265614
H	0.55914130563045	2.95940903901659	1.16563698402221

C	-0.22040857039740	2.56100180396685	-1.40652533735715
C	-0.14815040855210	3.66939714669878	-2.26923875612350
C	-1.45984557348312	2.22847318412781	-0.84327505111941
C	-2.60290227532771	2.96601811340654	-1.15608359935768
C	-2.53242920556037	4.05264947908874	-2.02589365133890
C	-1.29394383426171	4.39808577816888	-2.57742547811657
H	-3.42531040496056	4.62625080334693	-2.26679734888513
C	-1.20976578264946	5.60868610741106	-3.47582354714184
H	0.80576738480225	3.94439258118754	-2.71809176894200
H	-1.53308448782019	1.39841919115867	-0.14112963223800
C	-3.91477734207600	2.55315916823338	-0.53519434845852
C	-0.83335158195503	-1.17495357372824	-0.35010321298509
C	-0.53472003506035	-1.02098190396890	1.01877799763166
C	-1.86478070200573	-2.05162464107380	-0.72199298269754
C	-2.58245553186492	-2.76132083432682	0.23230175807502
C	-2.27258667346598	-2.59709157418115	1.59559772560672
C	-1.24206881938861	-1.71927817780033	1.98236612225027
H	-2.09460852843414	-2.15618997608345	-1.78281585223600
O	-2.91032744950089	-3.23679634644756	2.60786908268059
H	0.26104700265113	-0.34201791033368	1.32689795752962
C	3.86627606493417	7.04561994998045	3.52691892670788
H	4.81815165022522	6.49429830745984	3.57712922847605
H	3.71095603603809	7.59684265457056	4.45979234016802
H	3.88923707004468	7.74971006927882	2.68056372349908
F	-4.90667942371526	3.44857531846413	-0.76873353012861
F	-3.80429038634899	2.41619097840144	0.81780206084259
F	-4.33812005309599	1.34917592925218	-1.01369094780551

F	-0.08492280988468	5.61575505953227	-4.23245296491773
F	-1.20812276113896	6.76353183885006	-2.74596009515344
F	-2.27194728682296	5.68362196944848	-4.32259886890620
C	4.03069183045933	-3.54026687466341	-1.44310243642743
C	-3.97191785141318	-4.13669628875447	2.27098086229261
H	-4.78670463480496	-3.61107518776240	1.74911839304805
H	-4.33874831602240	-4.52918477681732	3.22470770904866
H	-3.60530245780221	-4.96612705026743	1.64626217761558
H	-3.37902971694606	-3.43294425679888	-0.08186875385330
H	-1.02172946083897	-1.60848492870992	3.04388891610746
C	-0.12709241133453	-0.40975791434937	-1.39670732386629
O	-0.42040282905192	-0.47305227508653	-2.58808286871433
H	2.79012366868448	1.06433836280506	1.05023307834523
F	6.05979304698574	-0.85175903832034	2.34300450475234
F	4.23656434303080	-0.01129187978049	3.21763917834309
F	5.44218945000632	1.18882155184509	1.84489349199959
F	3.42790158215102	-3.68187458180167	-2.64905920981725
F	3.63547209332369	-4.59644970226645	-0.67206249344411
F	5.37018190893120	-3.67934776000321	-1.63744940983403

Table S12. Associated vibrational frequencies for the optimized geometry of the **3'-E** isomer.

Mode	freq (cm^{-1})	Mode	freq (cm^{-1})	Mode	freq (cm^{-1})
8:	7.96	13:	15.97	18:	44.54
9:	8.86	14:	19.08	19:	62.18
10:	10.73	15:	23.38	20:	67.63
11:	12.39	16:	24.60	21:	74.63
12:	15.06	17:	41.67	22:	77.36

23: 88.60	50: 305.50	77: 577.24
24: 92.71	51: 307.15	78: 582.93
25: 97.59	52: 333.97	79: 603.82
26: 100.57	53: 335.13	80: 611.83
27: 113.08	54: 356.14	81: 625.04
28: 113.33	55: 358.58	82: 625.28
29: 130.01	56: 371.93	83: 648.37
30: 130.44	57: 390.67	84: 648.77
31: 141.24	58: 392.09	85: 649.92
32: 148.11	59: 397.20	86: 658.42
33: 154.34	60: 409.64	87: 660.38
34: 154.84	61: 410.33	88: 677.33
35: 177.94	62: 433.77	89: 678.52
36: 198.67	63: 446.33	90: 689.81
37: 199.31	64: 475.60	91: 691.16
38: 219.41	65: 477.37	92: 700.05
39: 221.78	66: 482.56	93: 703.49
40: 224.21	67: 482.65	94: 713.56
41: 230.87	68: 495.45	95: 739.43
42: 265.00	69: 500.71	96: 752.58
43: 271.61	70: 505.83	97: 781.41
44: 275.44	71: 507.59	98: 783.69
45: 278.94	72: 527.35	99: 793.37
46: 289.89	73: 543.39	100: 794.49
47: 290.52	74: 546.01	101: 800.89
48: 296.71	75: 552.68	102: 812.38
49: 304.68	76: 556.14	103: 825.26

104: 826.96
105: 831.80
106: 843.48
107: 888.48
108: 889.36
109: 897.85
110: 901.12
111: 911.96
112: 912.40
113: 927.05
114: 932.36
115: 935.41
116: 943.30
117: 947.33
118: 952.90
119: 992.63
120: 993.08
121: 993.73
122: 995.52
123: 1019.55
124: 1020.60
125: 1041.45
126: 1059.44
127: 1065.02
128: 1066.34
129: 1067.26
130: 1069.25
131: 1075.37
132: 1077.14
133: 1087.31
134: 1089.22
135: 1103.96
136: 1104.61
137: 1118.01
138: 1118.79
139: 1128.10
140: 1128.25
141: 1129.17
142: 1133.67
143: 1146.24
144: 1149.38
145: 1164.62
146: 1164.68
147: 1202.33
148: 1207.99
149: 1230.55
150: 1230.68
151: 1240.28
152: 1255.88
153: 1260.34
154: 1262.20
155: 1272.54
156: 1274.12
157: 1286.91
158: 1288.44
159: 1327.06
160: 1335.91
161: 1347.63
162: 1349.07
163: 1351.63
164: 1356.66
165: 1420.30
166: 1420.33
167: 1425.97
168: 1426.09
169: 1430.08
170: 1431.52
171: 1443.94
172: 1444.02
173: 1446.84
174: 1449.35
175: 1453.72
176: 1453.91
177: 1495.45
178: 1495.79
179: 1558.29
180: 1558.60
181: 1586.99
182: 1591.66
183: 1592.18
184: 1594.29

185: 1597.06

186: 1600.21

187: 1604.20

188: 1647.53

189: 1655.34

190: 2946.09

191: 2946.33

192: 3011.71

193: 3011.72

194: 3079.32

195: 3079.66

196: 3108.99

197: 3109.92

198: 3116.91

199: 3118.71

200: 3118.72

201: 3121.19

202: 3128.41

203: 3128.57

204: 3130.07

205: 3130.37

206: 3139.18

207: 3139.48

208: 3145.57

209: 3145.89

4. Optimized coordinates and associated vibrational frequencies of the **3’-Z** isomer

Table S13. optimized coordinates for the calculated geometry of the **3’-Z** isomer.

C	0.31589907157168	0.56354308288649	0.24210077410643
C	0.13019052734002	1.77587108989721	-0.34977133806668
C	1.29053355633265	2.64211075053564	-0.79797228832108
C	1.65570926966733	0.13868101110276	0.80934388696366
O	1.70980707106336	-0.12465249402918	2.01217365404238
O	1.36582918225551	2.91814868216985	-1.99764214635763
C	2.83579716767394	0.08728423343677	-0.06781381002800
C	4.11645167408039	0.10769013139969	0.52383806800647
C	2.73294506385632	0.06666103686184	-1.46580033800470
C	3.86916339941895	0.09384774221189	-2.26894369682551
C	5.13610551128522	0.15436272864300	-1.66626049221872
C	5.25138715032074	0.15167631134046	-0.26059811027564
H	6.24787013296226	0.19548850856618	0.17905645420108
O	6.30761126391372	0.22076274432609	-2.35009554849024
H	1.75030718134171	0.04557505781521	-1.93653642870559
H	3.76161294901394	0.08307503014436	-3.35132552706354
H	4.18382651039533	0.12122947166552	1.61161400107160
C	2.28868806364403	3.09152282880375	0.18484184936845
C	2.05773822600609	3.05216724322092	1.57372505193849
C	3.05323357880566	3.41366489197546	2.46483940625498
C	3.53943005596733	3.52618916618496	-0.28422322500069
C	4.55212660604093	3.87558372953012	0.59750478306004
C	4.31348952019100	3.81322830272116	1.98376533746706
H	2.88984044704509	3.38285644975152	3.54162558905371

O	5.23687803392235	4.11570460586047	2.93211726274585
H	3.70705850924342	3.55021006828989	-1.36101294400176
H	5.52254119577287	4.18002445432763	0.21053230542034
H	1.09135448428762	2.72275392278502	1.95483895587740
C	-1.22779681116055	2.29466346325423	-0.65381091966298
C	-1.57412598943548	2.78940873397308	-1.92025319574948
C	-2.20010759612248	2.28731923337697	0.35804013030204
C	-3.49819288290155	2.72365824068335	0.09825075018465
C	-3.85081280956283	3.19023816054953	-1.16899502448168
C	-2.87669682811833	3.22638583632838	-2.16825398590625
H	-4.86691159597900	3.52031441734364	-1.37366858202395
C	-3.21832595724600	3.77834618108153	-3.53162793374159
H	-0.82205001470636	2.80871650168034	-2.70616034048448
H	-1.93950976439952	1.92846014561227	1.35309820890418
C	-4.52518404533440	2.63341206039981	1.19980692266364
C	-0.80335434151045	-0.39487901949117	0.42826804959320
C	-1.08020429143346	-0.99092674050133	1.66668503088913
C	-1.61590467291102	-0.71192281007126	-0.67233081439737
C	-2.70142899136304	-1.57255203037236	-0.52774758063383
C	-2.99157990072783	-2.14902636295598	0.71128378273954
C	-2.17075454375838	-1.85416716928046	1.79955633927391
H	-1.40458391359250	-0.26571980283929	-1.64313917673390
C	-3.54148275612337	-1.90545775050980	-1.73621280441174
H	-3.84991723232700	-2.80721698700619	0.82736736700605
C	-2.43561464994785	-2.48426851820561	3.14624199962339
H	-0.45064768928516	-0.75521420957933	2.52232256200872
C	6.53756651307642	4.52416410678607	2.49753867347148

H	7.02542987069951	3.73339440449695	1.90616784449267
H	7.11071754094232	4.70495988360041	3.41250961781341
H	6.48611662909848	5.45075574936568	1.90456074959464
C	6.24694408900800	0.23203605241165	-3.78060374508858
H	5.78677062855406	-0.69159672403091	-4.16496826645129
H	7.28588893866127	0.29401474275909	-4.11977296761965
H	5.68404632791599	1.10507502337680	-4.14627458265145
F	-3.68927727835979	-2.99747255508506	3.23976114854821
F	-2.28749860113886	-1.58898562135209	4.15943872367037
F	-1.56638312363155	-3.50625725125346	3.39359326772665
F	-3.67533976085045	-0.83797756573023	-2.56969357408906
F	-4.79000925631434	-2.31509297127744	-1.39756728869943
F	-2.97850898105471	-2.90859776770240	-2.47246824582955
F	-5.65317350048412	3.33152849706594	0.91646249368852
F	-4.03982448895961	3.10603799193616	2.38104466666911
F	-4.89952704851286	1.33939718411043	1.42147109209093
F	-2.57042926027536	3.11612572332856	-4.52604624551391
F	-2.86390611238484	5.09235492635744	-3.63619912683696
F	-4.54896125146251	3.70608381494119	-3.79642650616546

Table S14. Associated vibrational frequencies for the optimized geometry of the **3'-E** isomer.

Mode freq (cm^{-1})

8:	13.05	13:	19.60	18:	55.48
9:	13.80	14:	20.64	19:	63.41
10:	16.20	15:	22.65	20:	77.47
11:	17.19	16:	30.06	21:	83.02
12:	18.83	17:	50.58	22:	84.75

23:	91.88	50:	306.72	77:	579.02
24:	96.37	51:	314.15	78:	586.10
25:	100.75	52:	330.78	79:	611.87
26:	106.86	53:	340.30	80:	613.23
27:	118.59	54:	361.99	81:	625.28
28:	125.92	55:	370.20	82:	629.76
29:	138.26	56:	373.53	83:	649.16
30:	138.99	57:	383.09	84:	649.21
31:	144.94	58:	394.12	85:	650.76
32:	151.51	59:	410.24	86:	655.78
33:	157.81	60:	413.74	87:	658.40
34:	164.78	61:	415.03	88:	660.01
35:	181.76	62:	434.26	89:	666.04
36:	200.22	63:	441.92	90:	691.14
37:	217.96	64:	450.34	91:	698.94
38:	221.61	65:	482.00	92:	700.88
39:	227.89	66:	482.82	93:	706.27
40:	238.63	67:	483.12	94:	713.11
41:	250.90	68:	496.41	95:	727.76
42:	265.44	69:	497.35	96:	768.22
43:	268.26	70:	502.70	97:	777.06
44:	268.43	71:	509.71	98:	780.46
45:	274.20	72:	516.65	99:	789.65
46:	285.09	73:	542.57	100:	793.39
47:	286.91	74:	544.95	101:	798.62
48:	302.66	75:	569.30	102:	819.36
49:	306.35	76:	574.04	103:	825.59

104: 827.90

105: 831.02

106: 848.73

107: 892.99

108: 893.89

109: 896.73

110: 902.53

111: 905.35

112: 921.01

113: 922.36

114: 925.91

115: 933.83

116: 940.82

117: 946.13

118: 952.33

119: 993.19

120: 993.76

121: 994.71

122: 995.97

123: 1018.68

124: 1021.67

125: 1035.39

126: 1063.18

127: 1064.63

128: 1070.07

129: 1071.34

130: 1073.46

131: 1074.65

132: 1081.89

133: 1085.61

134: 1096.80

135: 1103.82

136: 1105.71

137: 1114.27

138: 1117.05

139: 1127.91

140: 1128.06

141: 1130.64

142: 1132.05

143: 1150.27

144: 1153.79

145: 1164.25

146: 1164.68

147: 1179.09

148: 1205.21

149: 1227.49

150: 1230.27

151: 1255.28

152: 1259.44

153: 1261.36

154: 1263.84

155: 1269.45

156: 1271.17

157: 1285.42

158: 1288.78

159: 1329.04

160: 1337.57

161: 1342.86

162: 1347.03

163: 1348.21

164: 1352.66

165: 1420.97

166: 1423.31

167: 1424.23

168: 1425.74

169: 1426.81

170: 1427.61

171: 1443.17

172: 1443.67

173: 1443.71

174: 1444.27

175: 1453.90

176: 1454.80

177: 1493.00

178: 1495.68

179: 1555.28

180: 1556.09

181: 1557.79

182: 1589.76

183: 1591.30

184: 1591.65

185: 1594.17

186: 1596.12

187: 1597.96

188: 1632.99

189: 1636.10

190: 2944.25

191: 2945.77

192: 3008.71

193: 3010.99

194: 3078.30

195: 3079.01

196: 3115.79

197: 3116.88

198: 3119.00

199: 3120.86

200: 3126.05

201: 3128.91

202: 3130.26

203: 3132.43

204: 3139.72

205: 3144.36

206: 3146.28

207: 3148.19

208: 3150.35

209: 3151.21

5. Electron density map of the **1,2-Cp^{Ar5}** and **1,3-Cp^{Ar5}**anions

Electron density map obtained from DFT calculations (Orca 5.0.4. was used to optimize the geometry using BP86 functional and def2/J as the basis set)

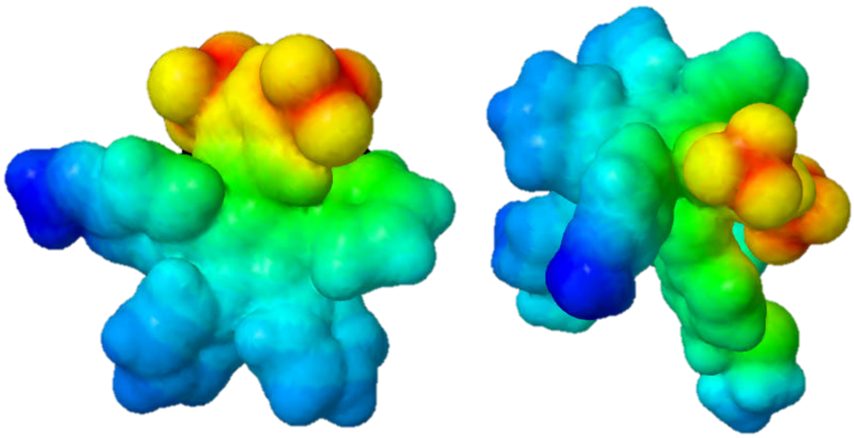

Figure S50. Electron density map of the 1,2-Cp^{Ar5} anion (left) and of the 1,2-Cp^{Ar5}[Ru]Tp complex (right).

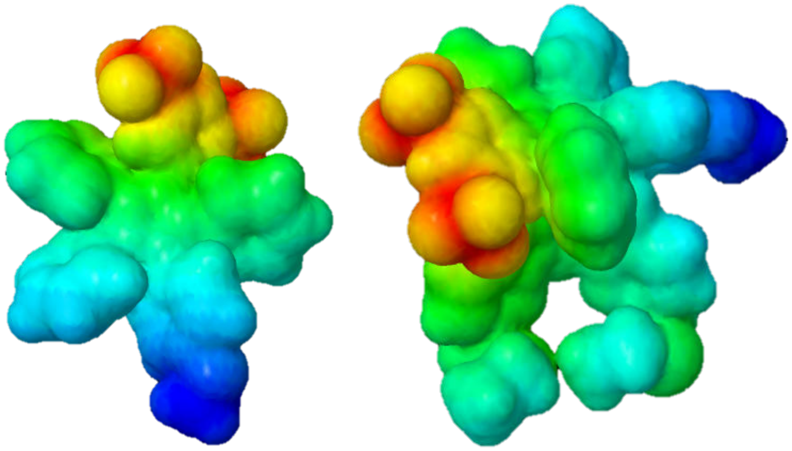

Figure S51. Electron density map of the 1,3-Cp^{Ar5} anion (left) and of the 1,3-Cp^{Ar5}[Ru]Tp complex (right).

V. References

[1] O. V. Dolomanov, L. J. Bourhis, R. J. Gildea, J. A. K. Howard, H. Puschmann, *J. Appl. Crystallogr.* **2009**, *42*, 339-341.

[2] G. Sheldrick, *Acta Crystallogr. Sect. A* **2015**, *71*, 3-8.

[3] G. Sheldrick, *Acta Crystallogr. Sect. C* **2015**, *71*, 3-8.

[4] Bruker, SADABS, Bruker AXS Inc., Madison, Wisconsin, USA, **2008**.

[5] A. L. Spek, *Acta Crystallogr. Sect. C* **2015**, *71*, 9-18.
